# Uncovering the acetylation sites of Dnmt3L that regulate protein stability and differentiation potency in embryonic stem cells

**DOI:** 10.1038/s12276-026-01655-w

**Published:** 2026-03-04

**Authors:** Yun Ji Nam, Hyungu Kwon, Hyun Jun Im, Naimur Rahman, Humaira Lubna, Siwon Lee, Hee-Sung Ahn, Gayoung Jang, YongHwan Kim, Hyein Ju, Seok Woo Ha, Hyun Ji Kim, Dabin Lee, Sang Jin Park, Sang Hoon Song, Juhyun Park, Yongsub Kim, Yoonjoo Choi, Kyunggon Kim, Dong-Myung Shin, Seungun Lee

**Affiliations:** 1 Department of Cell and Genetic Engineering, Brain Korea 21 Project, University of Ulsan College of Medicine, Asan Medical Center, Seoul, Republic of Korea; 2 Department of Microbiology and Immunology, Chonnam National University Medical School, Hwasun-gun, Republic of Korea; 3 Department of Biomedical Sciences, Chonnam National University Medical School, Hwasun-gun, Republic of Korea; 4 Department of Convergence Medicine, Asan Medical Center, University of Ulsan College of Medicine, Seoul, Republic of Korea; 5 Department of Urology, Asan Medical Center, University of Ulsan College of Medicine, Seoul, Republic of Korea; 6 National Immunotherapy Innovation Center, Hwasun-gun, Republic of Korea; 7 Department of Medical Informatics and Biostatistics, Brain Korea 21 Project, University of Ulsan College of Medicine, Asan Medical Center, Seoul, Republic of Korea; 8 Center for Cell Therapy, Asan Medical Center, Seoul, Republic of Korea

**Keywords:** Embryonic stem cells, Pluripotent stem cells, Stem-cell differentiation, Epigenetic memory

## Abstract

The epigenetic status, which regulates the cellular identity and differentiation potential of pluripotent stem (PS) cells, dynamically responds to the culture environment, affecting the safe and effective use of PS cells for basic research and therapeutic applications. However, the key mediator(s) representing the epigenetic signatures of PS cells under distinct culture conditions remains unclear. Here we investigated the role of DNA methyltransferase 3-like (Dnmt3L) in modulation of the DNA methylation and differentiation potential of mouse embryonic stem (ES) cells. Unlike other de novo DNA methyltransferases, Dnmt3L exhibited a uniquely dynamic expression pattern during prolonged 2i-leukemia inhibitory factor culture, which was marked by rapid post-transcriptional upregulation that sensitively reflected changes in the extracellular environment. Mass spectrometry identified that acetylation of lysine residues K238 and K412 controlled Dnmt3L protein stability. This site-specific acetylation critically modulated expression of genes associated with naive pluripotency and lineage differentiation—especially toward germline, neural and cardiac fates—through targeted DNA methylation and thereby orchestrated the lineage-specific developmental potential of mouse ES cells both in vitro and in vivo. Our findings demonstrate that Dnmt3L is a key regulator of epigenetic stability at developmentally critical loci in mouse ES cells and dynamically responds to changes in the extracellular culture environment. Thus, elucidation of the regulatory mechanism of Dnmt3L may provide insight into the onset of epigenetic aberrations and suggest the optimal culture conditions to preserve the epigenetic integrity of ES cells, which has significant implications for regenerative medicine.

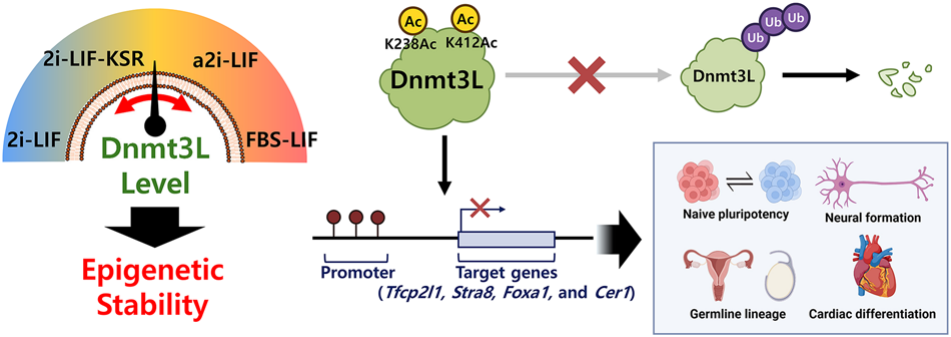

## Dnmt3L regulates epigenetic stability in stem cell cultures

Pluripotent stem (PS) cells can become any cell type, making them valuable for research and therapies. However, they face challenges such as oxidative stress and DNA changes during laboratory growth. This study explores how a protein called Dnmt3L affects PS cells’ stability and development. Researchers grew mouse embryonic stem cells under different conditions to see how Dnmt3L changes. They found that the stability of Dnmt3L is influenced by acetylation at specific sites, which affects the cells’ ability to develop into different tissues. The study used techniques such as mass spectrometry to identify these changes. Results showed that acetylation helps to maintain the stability of Dnmt3L, impacting DNA methylation and the cells’ developmental potential. Researchers conclude that understanding the role of Dnmt3L could improve PS cell use in research and medicine. Future work may explore how these findings apply to human stem cells, enhancing regenerative medicine applications.

This summary was initially drafted using artificial intelligence, then revised and fact-checked by the author.

## Introduction

Pluripotent stem (PS) cells are a valuable resource for developmental research and regenerative therapies because of their ability to differentiate into all cell types in the body. In addition, PS cells such as embryonic stem (ES) cells and induced PS (iPS) cells can be used for disease modeling and organoid generation, which could lead to personalized regenerative medicine^1^. PS cells, which arise briefly during early mammalian embryonic development, transition from a ‘naive/ground’ pluripotent state to a ‘primed’ pluripotent state before committing to a lineage^2^. PS cells in these two states differ in terms of their appearance, metabolism, epigenetic and transcriptional profiles and requirement for growth factors, indicating that PS cells in cultures are heterogeneous and dynamically fluctuate between different pluripotent states^3^. Furthermore, they are exposed to oxidative stress and develop epigenetic abnormalities over the course of ex vivo expansion, which could gradually impair their genetic and epigenetic integrity as well as their molecular and cellular phenotypes^4,5^. Therefore, identification of the key mediator(s) representing the molecular signatures of PS cells under distinct culture conditions would not only advance our understanding of pluripotency at the molecular level but also promote the safe and effective use of PS cells for basic research and therapeutic applications^6,7^.

DNA methylation is a crucial epigenetic mechanism for essential biological processes including embryonic development, gene regulation, suppression of transposable elements, X-chromosome inactivation, genomic imprinting and differentiation of stem cells^8^. DNA methylation occurs predominantly at CpG dinucleotides, forming 5-methylated cytosine (5mC), which is catalyzed by DNA methyltransferases (Dnmts). Dnmt1 is responsible for maintenance of DNA methylation, while Dnmt3A and Dnmt3B mediate de novo DNA methylation. Dnmt3-like (Dnmt3L) is catalytically inactive but regulates de novo methylation by stimulating the catalytic activities of de novo Dnmts. Intriguingly, expression of Dnmts is altered depending on the culture conditions, leading to distinct features of DNA methylation^2,5^. In particular, the regulatory elements of several developmentally crucial genes, including imprinted genes, lineage markers and tumor suppressors, are susceptible to the epigenetic alterations observed in PS cell cultures, which significantly affects the developmental potency and safety of PS cells. For example, 2i-leukemia inhibitory factor (LIF) culture conditions, which include two small molecule inhibitors of Mek1/2 and Gsk3 (PD0325901 and CHIR99021) and LIF, can capture naive pluripotency in vitro by inducing global DNA demethylation^9^. However, prolonged culture of mouse ES cells under 2i-LIF conditions results in significant loss of genomic imprints and impairs the developmental potential of these cells via dysregulation of Dnmts and cofactors^10,11^. Modified 2i-LIF cultures with a low concentration of the Mek1/2 inhibitor or its replacement by an Src inhibitor protect against defects in epigenetic integrity and the developmental potential of mouse ES cells.

Furthermore, metabolites in the culture medium can affect global epigenetic signatures and related pluripotent states. For example, vitamin C (VitC) promotes naive pluripotency by stimulating TET-dependent DNA demethylation^12^ and protects against abnormal DNA methylation of the *Dlk1*–*Dio3* imprinting region in mouse ES cells and iPS cells^13^ as well as in human ES cells^14^. A high VitC:L-proline ratio in the culture medium drives mouse ES cells toward a naive state, while an excess of L-proline captures a fully reversible early primed pluripotent state^15^. Previously, we reported that supplementation of ascorbic acid 2-glucoside, a stable VitC derivative, helps to stably support the naive pluripotency of mouse ES cells by not only inducing TET-mediated DNA demethylation but also activating Sirt1, a nicotinamide adenine dinucleotide^+^-dependent class III histone deacetylase (HDAC)^2^. Importantly, Sirt1 protects against aberrant DNA methylation in a subset of imprinted and germline developmental genes by antagonizing Dnmt3L via protein deacetylation^5^.

Here, we investigated the expression, function and post-translational modification (PTM) profiles of Dnmt3L in relation to protein stability and developmental potency of mouse ES cells. Specifically, long-term culture in 2i-LIF conditions rapidly induced Dnmt3L expression in a post-transcriptional manner, with a remarkable increase after re-exposure to serum-containing medium. Mass spectrometry analysis identified several acetylation and phosphorylation sites of Dnmt3L. In particular, acetylation of K238 (K238Ac) and K412 (K412Ac) of Dnmt3L critically affected protein stability, which contributed to the differentiation potency of mouse ES cells by specifically regulating transcription and DNA methylation of genes related to naive pluripotency and lineage differentiation. Therefore, the present study elucidates the role of specific acetylation sites in modulation of the stability and function of Dnmt3L, which can orchestrate epigenetic stability during ex vivo expansion or in vitro modeling.

## Materials and methods

### Cell culture

R1^5^ and gcOct4 mouse ES cells^16^ were grown in fetal bovine serum (FBS)–LIF medium, which was DMEM-high glucose (HyClone) supplemented with 2 mM L-glutamine, 20 mM HEPES, 1% MEM nonessential amino acid solution, 1% penicillin–streptomycin solution (Gibco), 0.1 mM β-mercaptoethanol (Sigma-Aldrich), 15% heat-inactivated FBS (HyClone) and 1,000 U/ml ESGRO/LIF (Millipore) on a 0.1% gelatin (Sigma-Aldrich)-coated tissue culture dish. To maintain naive pluripotency, mouse ES cells were maintained in 2i-LIF medium containing 3 µM CHIR99021 (BioGems) and 1 µM PD0325901 (BioGems) under the following two conditions on a 0.1% gelatin-coated tissue culture dish: FBS free or supplemented with knockout serum replacement (KSR) (Thermo Fisher Scientific).

To inhibit G9a activity, mouse ES cells were treated with 3 μM BIX01294 (Selleckchem) for 24 h. To block de novo translation and transcription, cells were treated with 200 μg/ml cycloheximide (CHX, Sigma-Aldrich) and 7.5 μg/ml actinomycin D (Sigma-Aldrich), respectively, for the indicated number of hours.

### Teratoma formation

All animal experiments were approved by the Institutional Animal Care and Use Committee of the University of Ulsan College of Medicine (approval no. IACUC 2021-12-271) and were performed in accordance with their guidelines and regulations. We subcutaneously injected 8-week-old male NOD-Prkdc^EM1^ mice (JA BIO) with 5 × 10^6^ of each type of mouse ES cell cultured in FBS–LIF medium. The mice and sites of injection were monitored for 46 days. Teratomas were recovered by dissection to measure tumor size and to perform histological examination or transcriptome analysis at day 21 after injection. Histological analyses and immunostaining were performed as previously described^5^. Mice were randomly allocated to treatment groups (*n* = 7 per group), and the orders of cell transplantation, treatment and evaluation, as well as daily examinations, were randomized. Investigators involved in teratoma size measurements and histological assessments were blinded to the treatment groups.

### LC–ESI–MS/MS analysis

To profile the PTM status of Dnmt3L, mouse ES cells overexpressing Flag-tagged Dnmt3L (Flag–Dnmt3L) were used in immunoprecipitation (IP) experiments. After SDS–polyacrylamide gel electrophoresis and staining with Coomassie Brilliant Blue (Bio-Rad), the lanes around 50 kDa corresponding to Flag–Dnmt3L were excised and the proteins in 25 mM ammonium bicarbonate were reduced with 10 mM dithiothreitol, alkylated with 55 mM indole-3-acetic acid and in-gel digested with trypsin/LysC overnight at 37 °C. Peptides were extracted, desalted with a Sep-Pak C18 1 cc Vac cartridge, 100 mg (Waters) and lyophilized. Peptide samples were stored at −80 °C until further analysis. Peptide samples were separated using a Dionex UltiMate 3000 RSLCnano system (Thermo Fisher Scientific). Tryptic peptides from the bead column were reconstituted in 0.1% formic acid and separated on an Acclaim PepMap 100 C18 column (500 mm × 75 μm inner diameter, 3 μm, 100 Å) equipped with a C18 PepMap trap column (20 mm × 100 μm inner diameter, 5 μm, 100 Å; Thermo Fisher Scientific) over 200 min (250 nl/min) using a 0–48% acetonitrile gradient in 0.1% formic acid and 5% dimethylsulfoxide for 150 min at 50 °C. Liquid chromatography (LC) was coupled to a Q Exactive Plus Hybrid Quadrupole-Orbitrap mass spectrometer (Thermo Fisher Scientific) with a nano-electrospray ionization (ESI) source. Mass spectra were acquired in a data-dependent mode with an automatic switch between a full scan and 20 data-dependent tandem mass spectrometry (MS/MS) scans. The target value for the full scan MS spectra, selected from 350 *m*/*z* to 1800 *m*/*z*, was 3,000,000 with a maximum injection time of 100 ms and a resolution of 70,000 at *m*/*z* 400. The selected ions were fragmented by higher-energy collisional dissociation with the following parameters: 1.7 Da precursor ion isolation window and 27% normalized collision energy. The ion target value for MS/MS was set to 1,000,000 with a maximum injection time of 50 ms and a resolution of 17,500 at *m*/*z* 400. Repeated peptides were dynamically excluded for 20 s to reduce redundancy of the spectrum.

Four LC–MS raw files were acquired (two samples, each sample injected twice). Data analysis was performed using MetaMorpheus version 0.0.320, which is available at https://github.com/smith-chem-wisc/MetaMorpheus. The following search settings were used: protease = trypsin; maximum missed cleavages = 3; minimum peptide length = 7; maximum peptide length = 50; fixed modifications = carbamidomethyl on C; variable modifications = oxidation on M, acetylation on K, acetylation on X and phosphorylation on S, T and Y; maximum modifications per peptide = 7; maximum modification isoforms = 1,024; precursor mass tolerance = ±10 ppm; product mass tolerance = ±0.02 Da; and report peptide–spectrum match ambiguity = true. The combined search database contained two nondecoy protein entries (Dnmt3L-wild type (WT) and Dnmt3L-K8R) including 301 contaminant sequences. Dnmt3L-K8R refers to a protein in which eight lysine residues (K57, K111, K214, K238, K255, K367, K376 and K401) have been substituted with arginine by site-directed mutagenesis.

### IP pulldown assay

Whole-cell extracts were prepared using IP lysis buffer (20 mM HEPES (pH 7.4), 0.5% NP-40, 0.5% Triton X-100, 150 mM NaCl, 1.5 mM MgCl_2_, 1 mM dithiothreitol and 2 mM EDTA) supplemented with protease and phosphatase inhibitor mixtures as well as 2.5 mM NaB and then centrifuged (12,000*g* for 10 min at 4 °C). The extracts were incubated with Anti-Flag M2 Magnetic Beads (Sigma-Aldrich) or Protein G Magnetic Bead (Millipore) mixed with 0.1 μg of a hemagglutinin (HA)-specific antibody for 2 h at 4 °C. The IP products were washed five times with IP lysis buffer, and bound proteins were eluted with buffers containing 3× Flag peptide or HA peptide (Sigma-Aldrich) according to the manufacturer’s instructions.

### Ectopic expression

To overexpress Flag–Dnmt3L, murine *Dnmt3L* cDNA was subcloned into the pCMV_3Tag-1 vector^5^. R1 mouse ES cells stably expressing the plasmid were established by transfection using Lipofectamine 2000 (Invitrogen) followed by selection in the presence of 1 mg/ml G418 Geneticin (Invitrogen) for 2 weeks. Murine *Dnmt3L* K238R and K412R mutant open reading frames were generated by site-directed mutagenesis (iNtRON Biotechnology, Seongnam-si, Gyeonggi-do, Korea). The WT, K238R and K412R open reading frames were subcloned into the pENTR4 plasmid (Invitrogen) and then cloned into the pEZ-Lv235 lentiviral vector containing a CAG promoter (GeneCopoeia plasmid EZ016) using the Gateway Technology reaction in accordance with the manufacturer’s instructions (Invitrogen). Lentiviruses were produced using a three-plasmid transfection system (Invitrogen). A total of 2 days after transfection into the 293 FT packaging cell line, supernatants containing recombinant pseudo-lentiviral particles were collected and concentrated by precipitation using a Lenti-X Concentrator Kit (Clontech). The concentrated virus was infected into R1 mouse ES cells using 6 μg/ml polybrene (Invitrogen), and infected cells were selected by maintaining the cells for ≥3 weeks in the presence of 1 μg/ml puromycin (Invitrogen). The effects of ectopic expression were examined by reverse transcription (RT)–quantitative polymerase chain reaction (qPCR) and western blot analyses.

### RNAi

For the RNA interference (RNAi)-mediated gene knockdown assay, short hairpin (sh)RNAs against murine *Prdm14* were cloned into the pLenti6/Block-iT lentiviral vector (Invitrogen/Thermo Fisher Scientific) as previously described^17,18^. The sequences of the shRNAs are presented in the key resources table in the Supplementary information.

### Statistical analysis

Statistical significance was determined by a one-way or two-way analysis of variance (ANOVA) with the Bonferroni post hoc test using GraphPad Prism 7.0 (GraphPad Software). Significance was assumed for ^*^P < 0.05, ^**^P < 0.01 and ^***^P < 0.001. All quantification of raw data and statistical analyses, including tests used, sample sizes and exact *P* values, are presented in the Source Data, which are provided as a separate Excel file.

Details of other experimental procedures are provided in the Supplementary Materials and Methods.

## Results

### Post-transcriptional induction of Dnmt3L during prolonged 2i-LIF culture

**Fig. 1:**
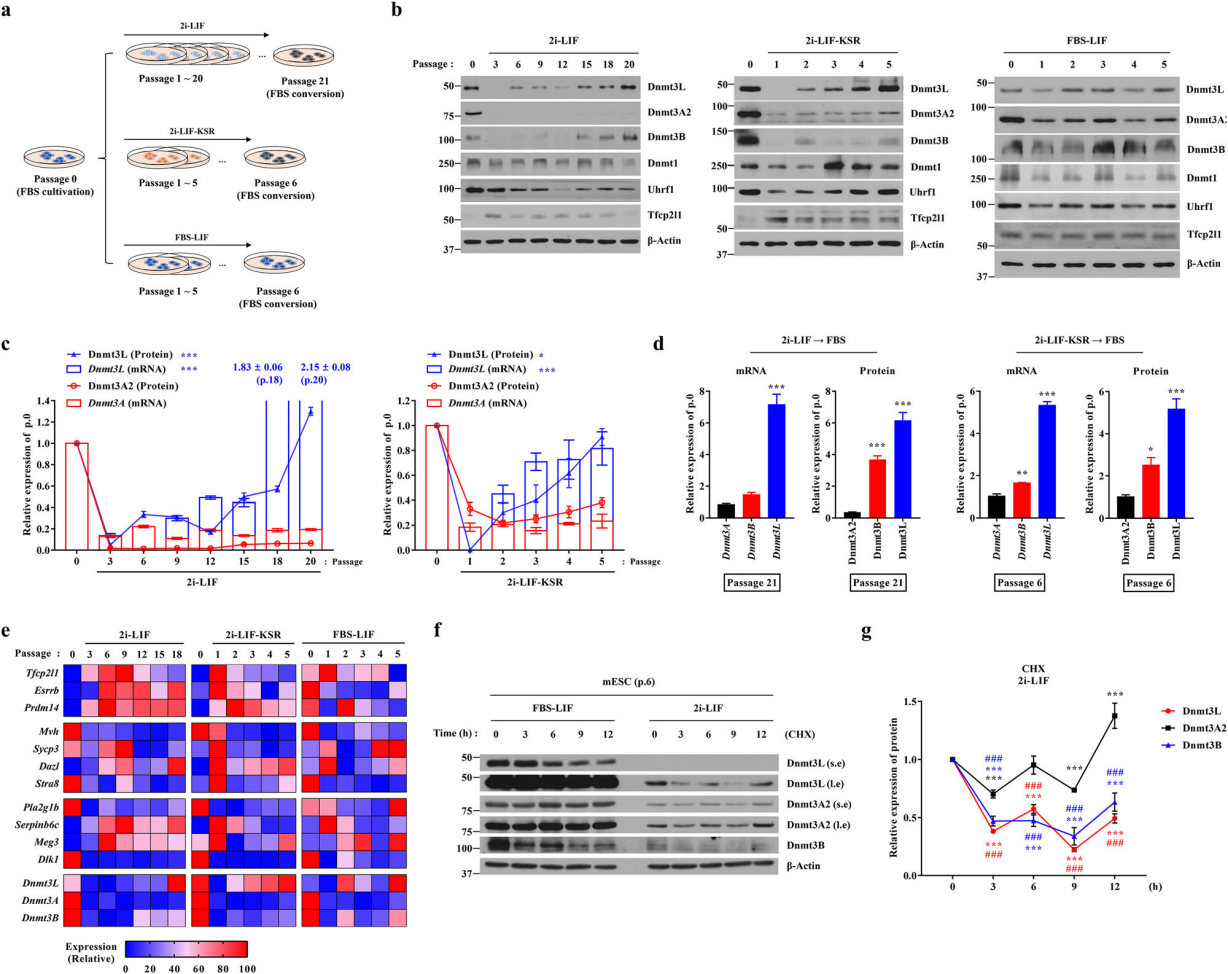
Dynamic post-transcriptional regulation of Dnmt3L expression under prolonged 2i-based culture conditions. **a** Schematic overview of three distinct culture conditions used for mouse ES cell propagation: FBS–LIF, 2i-LIF and 2i-LIF-KSR. **b** Western blot analysis of Dnmt proteins and cofactors in R1 mouse ES cells maintained under the indicated conditions at early and late passages. Molecular weight marker sizes (kDa) are indicated on the left. **c** Temporal dynamics of Dnmt3L and Dnmt3A expression at the transcript (bars) and protein (lines) levels during long-term propagation in 2i-LIF (left) and 2i-LIF-KSR (right) conditions (*n* = 4). Outlier values falling outside the plotted range are indicated as numbers above the corresponding passages. **d** Reactivation of Dnmt3L and other de novo Dnmts at the transcript and protein levels upon re-exposure to FBS–LIF after extended 2i-LIF or 2i-LIF-KSR culture (*n* = 4). **e** Heat map illustrating the expression changes of transcripts associated with pluripotency, germline development, genomic imprinting and DNA methylation under distinct culture conditions. Expression values were calculated from four independent biological replicates. **f** CHX-chase assay to assess the protein stabilities of Dnmt3L and Dnmt3A2 in mouse ES cells at p6 under the 2i-LIF condition. Short (s.e) and long (l.e) exposures are shown. **g** Quantitative analysis of Dnmt3L (red), Dnmt3A2 (black) and Dnmt3B (blue) protein levels from the CHX-chase assay (*n* = 4). Protein expression was normalized to β-actin. Quantitative data are represented as mean ± standard error of the mean (s.e.m.). Statistical analysis was performed using a two-way (**c** and **g**) or one-way (**d**) ANOVA with the Bonferroni post hoc test. ^*^P < 0.05, ^**^P < 0.01, ^***^P < 0.001 compared with control groups. ^###^*P* < 0.001 compared with Dnmt3A2. Exact *P* values and replicate numbers are provided in the Source Data. See also Supplementary Figs. 1 and 2.

To investigate how the composition and length of culture contribute to aberrant DNA methylation, we examined the expression of Dnmts and their cofactors in R1 mouse ES cells that were maintained in FBS-containing medium supplemented with LIF (FBS–LIF) without a feeder layer and then subsequently propagated for an additional 5 or 20 passages (p.5 or p.20) in three culture conditions (Fig. 1a). These included (1) 2i-LIF without FBS, which maintains naive pluripotency but induces global hypomethylation and imprint erosion during prolonged culture^10,11^; (2) 2i-LIF-KSR, in which serum is replaced by KSR to reduce epigenetic instability associated with 2i conditions^19^; and (3) FBS–LIF, which sustains a heterogeneous, partially primed pluripotent state. To assess the reversibility of any observed changes, we also switched mouse ES cells cultured under the p.20 2i-LIF or p.5 2i-LIF-KSR condition back to the FBS–LIF condition for additional passage.

Gene expression analysis revealed that expression of major de novo Dnmts such as Dnmt3A2 and Dnmt3B and their cofactor Dnmt3L was rapidly suppressed at both the RNA and protein levels in mouse ES cells under both 2i-LIF conditions (Fig. 1b, c). Subsequently, the repression of Dnmt3A2 and Dnmt3B persisted during further propagation, except that Dnmt3B protein was detected at later passages (> p.15) under the 2i-LIF conditions (Supplementary Fig. 1a). Unlike de novo Dnmts, the transcript and protein levels of Dnmt3L rapidly increased in early passages and gradually returned to the levels observed in the initial (p.0) passage (Fig. 1c). Notably, these increases in the transcript and protein levels of Dnmt3L became more pronounced upon re-exposure to FBS–LIF medium in both 2i-LIF culture conditions, with an approximately fivefold elevation compared with p.0 cells (Fig. 1d and Supplementary Fig. 1b).

Expression of Dnmt1, which is responsible for maintenance of DNA methylation, was not markedly affected by 2i-LIF culture but was induced at the late passage in 2i-LIF-KSR culture (Fig. 1b). Interestingly, the expression pattern of Uhrf1 protein, a cofactor of Dnmt1^20^, was similar to that of Dnmt3L protein, gradually decreasing and then returning to initial levels under both 2i-LIF conditions, suggesting that a distinct regulatory mechanism governs expression of DNA methylation cofactors in mouse ES cells. Expression of Dnmts and their cofactors stochastically fluctuated during propagation of mouse ES cells in FBS–LIF medium (Fig. 1b).

As reported previously^21^, exposure of mouse ES cells to 2i-LIF conditions upregulated genes associated with naive pluripotency, including *Tfcp2l1*, *Esrrb* and *Prdm14*, as well as germline markers, including *Sycp3* and *Dazl* (Fig. 1e and Supplementary Fig. 1c). Notably, expression of *Tfcp2l1*, *Sycp3* and *Serpinb6c*, which was initially upregulated early during exposure to 2i-LIF, gradually diminished with prolonged culture in both 2i-LIF and 2i-LIF-KSR conditions. This progressive downregulation exhibited a clear inverse relationship with the late-passage upregulation of *Dnmt3L*, suggesting that aberrant Dnmt3L induction compromises the transcriptional maintenance of key pluripotency and germline-associated genes.

Prolonged Mek1/2 suppression impairs the developmental potential of ES cells, partly through downregulation of Dnmts and their cofactors, which is mitigated by substituting the Mek1/2 inhibitor with an Src inhibitor, CGP77675 (a2i-LIF), or a PKC inhibitor, Gö6983 (PKCi-LIF)^10^. Thus, we examined the impact of replacing the Mek1/2 inhibitor with these inhibitors on expression of Dnmts and their cofactors. Mouse ES cells cultured in both the a2i-LIF and PKCi-LIF conditions exhibited elevated protein levels of Dnmt3L, as well as de novo Dnmts and Uhrf1, compared with those cultured in the 2i-LIF condition (Supplementary Fig. 1d,e). Expression of naive pluripotency genes was inversely correlated with the increased levels of Dnmt3L (Supplementary Fig. 1f), suggesting that Dnmt3L is an important indicator of DNA methylation alterations under different culture conditions in mouse ES cells.

Interestingly, the transcription and translation levels of Dnmt3L were unsynchronized during recovery of its expression in long-term cultures under both 2i-LIF conditions (Fig. 1c). For example, in 2i-LIF-KSR culture, the recovery of Dnmt3L protein was followed by induction of *Dnmt3L* transcription. In 2i-LIF culture, Dnmt3L protein was rapidly recovered at p.6, while *Dnmt3L* transcription was suppressed. Consistently, inhibition of de novo transcription with actinomycin D had minimal impact on Dnmt3L protein induction in mouse ES cells at p.6 in 2i-LIF culture (Supplementary Fig. 1g). By contrast, treatment with CHX significantly reduced Dnmt3L protein induction, suggesting that Dnmt3L and Dnmt3B induction is regulated post-transcriptionally in early passages under the 2i-LIF condition (Fig. 1f, g). Collectively, these observations indicate that, unlike other Dnmts, Dnmt3L displays a uniquely dynamic expression pattern during extended 2i-LIF cultures, which is primarily governed by post-transcriptional regulatory mechanisms.

### Acetylation profiles of Dnmt3L protein in mouse ES cells

We previously reported that a substantial amount of Dnmt3L protein is acetylated in mouse ES cells^5^. To further investigate the temporal association between Dnmt3L acetylation and its protein stability under prolonged 2i-LIF culture, we performed in situ proximity ligation assays to visualize acetylated Dnmt3L protein. Compared with FBS–LIF conditions, proximity ligation assay signals indicative of acetylated Dnmt3L were markedly elevated under 2i-LIF conditions, particularly at the intermediate (p.6) passage (Supplementary Fig. 2a–c). To biochemically validate this observation, we established mouse ES cell lines stably expressing cytomegalovirus (CMV) promoter-driven Flag–*Dnmt3L*, thereby minimizing transcriptional variability. While exogenous transcript levels remained stable across passages (Supplementary Fig. 2d,e), acetylation of Flag–Dnmt3L significantly increased under 2i-LIF compared with FBS–LIF conditions, particularly at early (p.3) and intermediate (p.6) stages (Supplementary Fig. 2f,g). Notably, elevated acetylation coincided with the recovery of Dnmt3L protein at p6 despite continued transcriptional repression, suggesting that acetylation enhances protein stability during adaptation to 2i-LIF conditions. Moreover, comparison with a modified culture system (a2i-LIF), in which Mek1/2 inhibition was replaced by an Src inhibitor, revealed a partial reduction of Dnmt3L hyperacetylation (Supplementary Fig. 2h,i), implying that Mek1/2 inhibition contributes to the enhanced acetylation observed under standard 2i-LIF conditions.

**Fig. 2:**
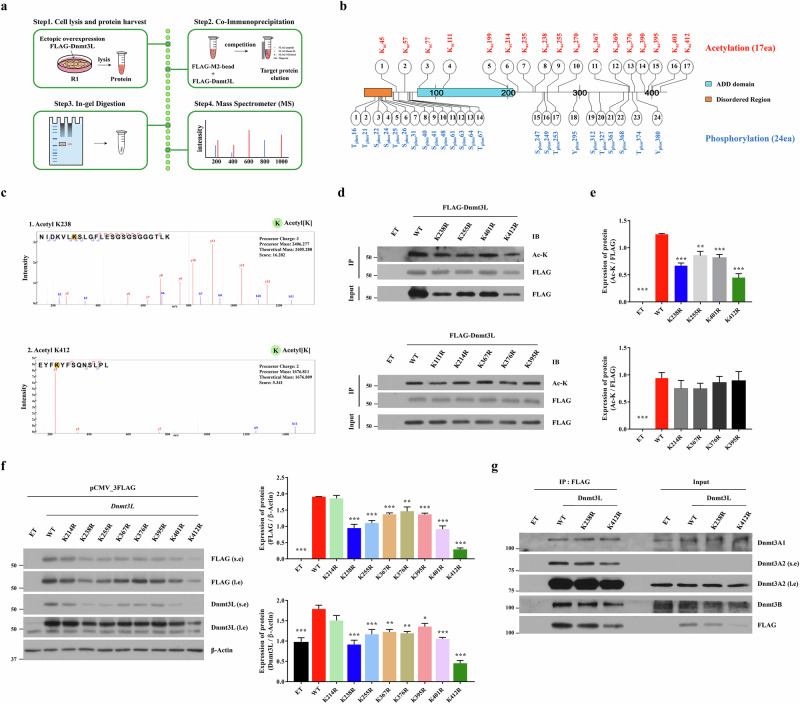
Identification of critical acetylation sites that regulate Dnmt3L protein stability. **a** Schematic overview of the experimental strategy to identify lysine acetylation sites in Dnmt3L protein expressed in mouse ES cells. **b** Summary of acetylated lysine (K) and phosphorylated serine (S), threonine (T) and tyrosine (Y) residues identified by mass spectrometry from Flag-IP Dnmt3L proteins in Flag–*Dnmt3L-*overexpressing R1 mouse ES cells. Detected sites are mapped on a schematic representation of full-length Dnmt3L protein (421 amino acids). ADD, ATRX–Dnmt3–Dnmt3L domain. **c** Representative MS/MS spectra and corresponding peptide sequences of acetylated K238 (top) and K412 (bottom) residues. Red and blue lines indicate *y* and *b* ions, respectively. The positions of lysine residues in a peptide are denoted as orange circles. **d** IP assay to assess lysine acetylation of Flag-tagged WT Dnmt3L and lysine-to-arginine (K → R) mutant proteins in mouse ES cells. **e** Quantification of acetylated levels of the indicated Dnmt3L proteins normalized to the corresponding Flag–Dnmt3L protein levels (*n* = 3). **f** Western blot analysis of Flag–Dnmt3L WT and K → R mutant proteins in mouse ES cells. β-actin was used as a loading control. Among the tested mutants, K238R and K412R displayed a consistent reduction in protein levels (*n* = 3). **g** Co-IP assay to assess physical interactions between Flag–Dnmt3L (WT, K238R and K412R) and de novo Dnmt3A and Dnmt3B. Input lanes contain 20% of the total lysate used for IP. Quantitative values are shown as means ± s.e.m. ^*^P < 0.05, ^**^P < 0.01, ^***^P < 0.001 compared with the WT Dnmt3L group by a one-way ANOVA with the Bonferroni post hoc test. See also Supplementary Fig. 3.

In this regard, to profile the PTM status of Dnmt3L, we ectopically expressed Flag–Dnmt3L in mouse ES cells and analyzed anti-Flag-IP products by mass spectrometry (Fig. 2a and Supplementary Fig. 3a). Including the previously identified acetylated lysine residues (AcK)^5^, 17 AcK sites were detected, in parallel with 24 phosphorylation sites (Fig. 2b, c and Supplementary Fig. 3b). To identify the critical AcK site(s), we mutated each lysine residue to arginine and then ectopically expressed the Flag–*Dnmt3L* mutants in mouse ES cells. Compared with WT Dnmt3L protein, site-directed mutagenesis of lysine residues 238, 255, 401 and 412 to arginine (K238R, K255R, K401R and K412R) led to reduced protein expression and a corresponding decrease in protein acetylation (Fig. 2d, e). By contrast, acetylation levels were similar in cells expressing the other Dnmt3L mutants, suggesting that acetylation of Dnmt3L exhibits functional redundancy in mouse ES cells.

Notably, ectopic expression of the *Dnmt3L* mutant harboring the K238R or K412R substitution led to a pronounced reduction in the protein level (Fig. 2f), despite comparable messenger RNA expression of all constructs (Supplementary Fig. 3c). Similar hypo-expression was observed for the K255R and K401R mutants, whereas the other lysine mutants showed mild reductions in the protein level (Fig. 2f). Overexpression of the *Dnmt3L* mutants tested in this study did not markedly affect the expression levels of other Dnmts, including Dnmt3A2, Dnmt3B and Dnmt1 (Supplementary Fig. 3d). These findings prompted us to focus on K238Ac and K412Ac as critical regulatory sites that influence the protein stability and function of Dnmt3L in mouse ES cells.

We examined whether K238Ac and K412Ac contribute to a defect in the nuclear localization of Dnmt3L, leading to its hypo-expression. Immunofluorescence (IF) staining revealed that all exogenous Flag–Dnmt3L proteins had a similar nuclear distribution (Supplementary Fig. 3e), which excluded this possibility. The phosphorylation status of Dnmts can regulate their ubiquitination and protein stability^22^. In Flag-IP products of the Dnmt3L K238R and K412R mutants, phosphorylation at threonine, tyrosine or serine residues was reduced compared with WT Dnmt3L (Supplementary Fig. 3f). Quantification analysis indicated that given the markedly lower protein expression of these mutants, this decrease might reflect the diminished protein abundance rather than an intrinsic alteration in phosphorylation status, suggesting that the K238R and K412R mutations have minimal direct impact on Dnmt3L phosphorylation (Supplementary Fig. 3g). In addition, the Flag-IP assay revealed that the Dnmt3L K238R and K412R mutants properly interacted with endogenous Dnmt3A and Dnmt3B proteins in mouse ES cells (Fig. 2g). Collectively, these results demonstrate the Dnmt3L K238R and K412R mutants are hypo-expressed, but this does not involve changes in their subcellular localization, phosphorylation status or interactions with de novo Dnmt proteins.

### Structural stability and flexibility of Dnmt3L variants

**Fig. 3:**
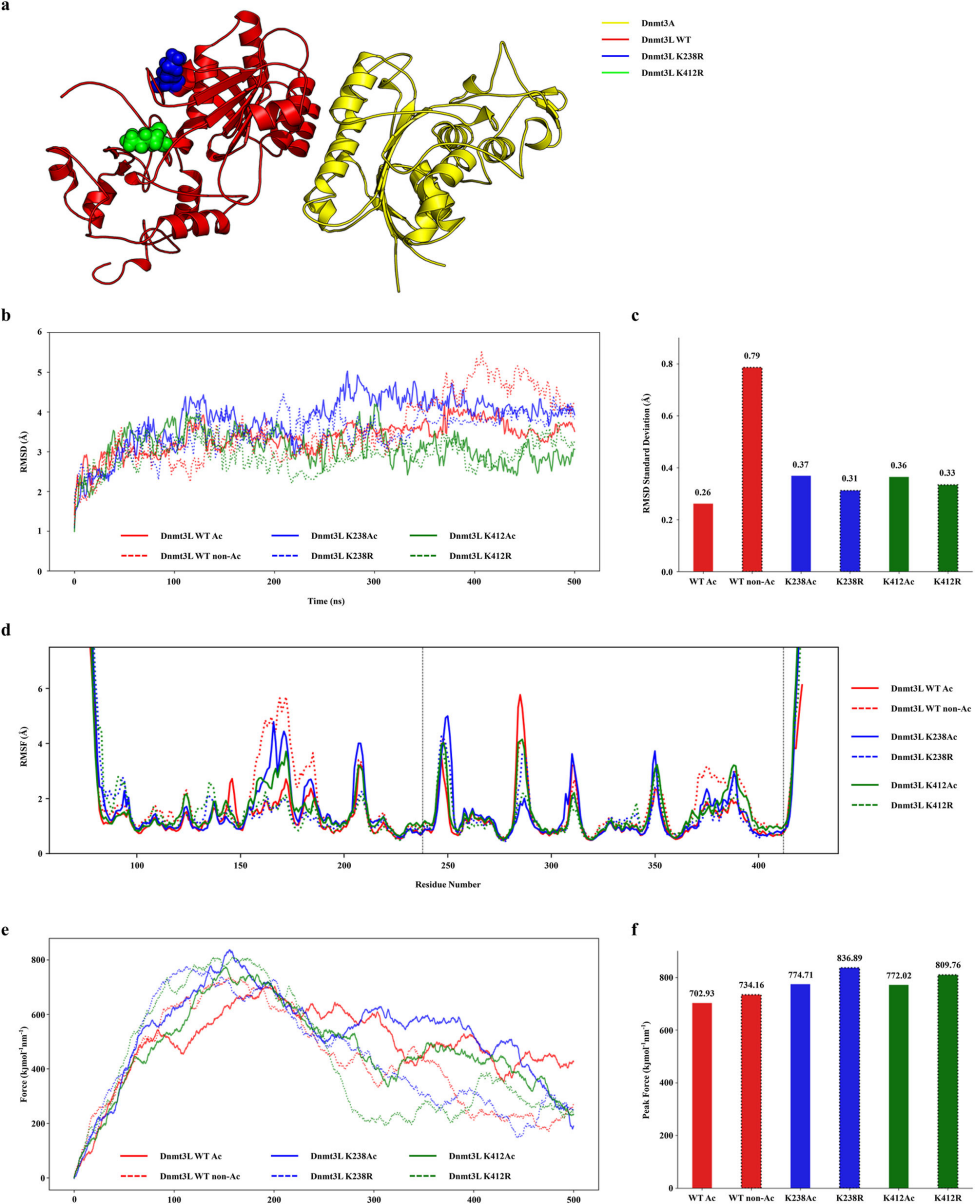
MD reveals altered structural flexibility of Dnmt3L variants. **a** Structure of the Dnmt3A–Dnmt3L complex highlighting the Dnmt3L mutation sites K238 and K412. **b** RMSD profiles over 500-ns simulations, illustrating structural fluctuations of WT Ac Dnmt3L and the other mutants. **c** Bar plot showing the standard deviation values of RMSD (after 100 ns), indicating that overall flexibility was higher in both mutants than in WT Ac Dnmt3L. **d** RMSF per residue, indicating local flexibility differences across the protein sequence. **e**, **f** SMD pulling simulations. Pulling force profiles comparing WT Ac Dnmt3L with each mutant, showing the time-dependent force experienced during pulling simulations (**e**) and peak pulling forces extracted from SMD simulations, indicating that mechanical resistance was comparable among WT Ac Dnmt3L and mutant complexes (**f**). See also Supplementary Fig. 4.

To gain structural insight into hypo-expression of the Dnmt3L mutants, we performed extensive molecular dynamic (MD) simulations of fully acetylated (WT Ac), nonacetylated (WT non-Ac), K238Ac and K412Ac Dnmt3L, as well as the K238R and K412R mutants (Fig. 3a). Root mean square deviation (RMSD) analysis over 500 ns revealed clear differences in stability among the six proteins. WT Ac Dnmt3L equilibrated to a relatively stable conformation with RMSD fluctuating within ~3–4 Å after an initial settling period (Fig. 3b). By contrast, partially acetylated (K238Ac and K412Ac) and the arginine mutants (K238R and K412R) exhibited relatively larger structural deviations, especially at later simulation times (RMSD ranged ~2.5–5 Å beyond 200 ns), indicative of enhanced conformational flexibility and reduced stability. According to the average RMSD amplitude differences for each variant (Fig. 3c), WT Ac Dnmt3L showed the smallest RMSD deviation (~0.26 Å), whereas those of partial acetylated and arginine mutants were slightly elevated (from 0.31 to 0.37 Å, respectively). This higher RMSD amplitude of both mutants reflects greater overall structural variability and lower global stability compared with WT Ac Dnmt3L. Notably, the most unstable variant was the nonacetylated one (WT non-Ac), whose RMSD deviation value is tripled (0.79 Å) compared with the WT Ac, indicating that acetylation contributes to stability.

We next examined root mean square fluctuation (RMSF) per residue to pinpoint regions of altered flexibility (Fig. 3d and Supplementary Fig. 4). Consistent with the RMSD results, the partially acetylated and nonacetylated (WT non-Ac) variants had generally elevated RMSF values across multiple regions relative to WT Ac Dnmt3L, implying that these mutations increase local mobility throughout the protein. Notably, all six proteins maintained low fluctuation at the C-terminal helix responsible for Dnmt3A binding, indicating that the core interface remains relatively stable.

### Dnmt3L–Dnmt3A binding mechanics from MD simulations

We next probed whether the mutations affect the interaction of Dnmt3L with its binding partner Dnmt3A^23^. Steered MD (SMD) pulling simulations were performed of the Dnmt3A–Dnmt3L complex to evaluate the mechanical stability of the interface. In these simulations, Dnmt3L WTs and variants were pulled away from Dnmt3A with a constant velocity, and the required force was recorded as a function of extension. All six Dnmt3L proteins exhibited a similar force–extension profile, indicative of a comparable interaction mechanism. The force on the complex initially increased steadily with extension, rose to a single rupture peak as key contacts broke and then decreased once Dnmt3L dissociated (Fig. 3e). The overall shapes of the force curves were highly similar, suggesting that the mutations do not grossly alter the Dnmt3L–Dnmt3A binding interface under mechanical stress. There were slight differences in the peak forces required to separate the complex. These peak rupture forces were quantified for each variant (Fig. 3f). The K238R required the highest force (~836.89 kJ/mol/nm) to disrupt the complex, followed by the K412R mutant (~809.76 kJ/mol/nm), while WT Ac Dnmt3L had the lowest peak force (~702.93 kJ/mol/nm). However, such marginal variations indicate that binding strength is largely preserved across all variants, indicating that the mutations and acetylation do not drastically alter the mechanical stability of the complex.

### K238Ac or K412Ac is essential for protein stability of Dnmt3L

**Fig. 4:**
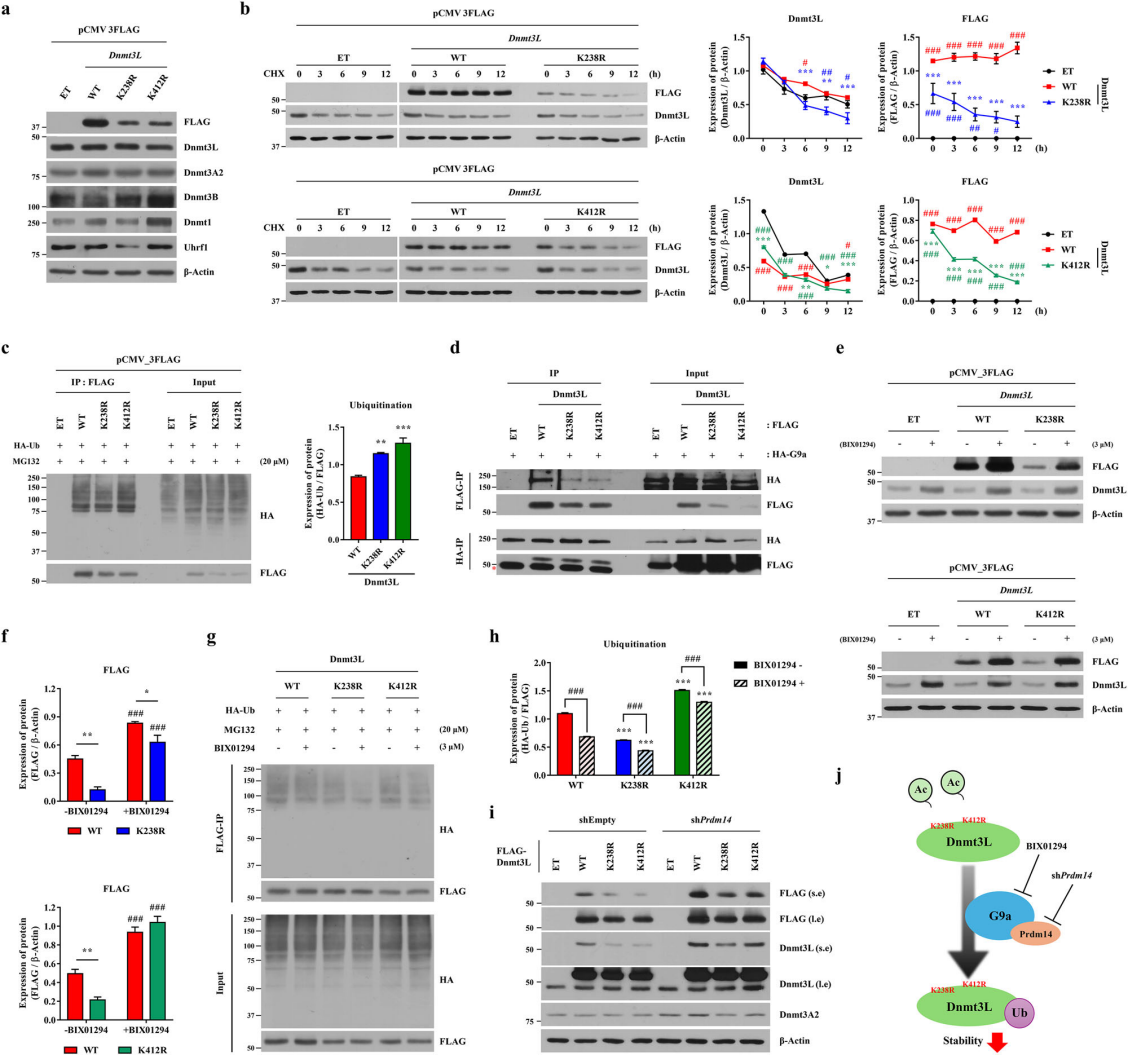
G9a and Prdm14 cooperatively regulate Dnmt3L protein stability. **a** Western blot analysis of mouse ES cells stably expressing Flag-WT Dnmt3L and acetylation-deficient mutants (K238R and K412R). **b** CHX-chase assay assessing the stabilities of Flag–Dnmt3L WT and mutant proteins. Left: time-course immunoblotting following treatment with 200 µg/ml CHX. Right: quantification of protein levels normalized to β-actin (*n* = 3). Both mutants exhibited shorter half-lives than WT Dnmt3L. **c** Flag-IP assay showing the ubiquitination status of WT and mutant Dnmt3L proteins after cotransfection with HA-Ub. Quantification of the indicated HA-Ub tagged protein levels normalized to Flag (*n* = 3). Input lanes contain 20% of total lysates. **d** Co-IP assays demonstrating physical interactions between HA-G9a and Flag–Dnmt3L variants. Flag-based IP (top) and reciprocal HA-based IP (bottom), confirming binding irrespective of the mutation status. Asterisks indicate nonspecific immunoglobulin G heavy chain bands. **e** Immunoblot analysis of exogenous and endogenous Dnmt3L protein levels after pharmacological inhibition of G9a using 3 µM BIX01294 for 24 h. **f** Quantification of the indicated Flag–Dnmt3L protein levels normalized to β-actin (*n* = 3). **g**, **h** Flag-IP assay of WT Dnmt3L and mutant proteins following treatment with 3 μM BIX01294 for 12 h to assess changes in polyubiquitination levels (**g**). Quantitative analysis of Dnmt3L ubiquitination normalized to IP recovery of each Flag–Dnmt3L variant (*n* = 3) (**h**). **i** Western blot analysis of WT Dnmt3L and mutant protein expression in mouse ES cells upon *Prdm14* knockdown using shRNA. **j** Schematic model showing how G9a- and Prdm14-dependent post-translational mechanisms coordinately control the stability of Dnmt3L protein. All quantitative data represent means ± s.e.m. Statistical significance was assessed using a two-way ANOVA with the Bonferroni post hoc test. ^*^P < 0.05, ^**^P < 0.01, ^***^P < 0.001 compared with WT Dnmt3L group. ^#^*P* < 0.05, ^##^*P* < 0.01, ^###^*P* < 0.001 compared with empty (**b**) or nontreated control (**f** and **h**) groups. See also Supplementary Figs. 5–7.

To obtain mechanistic insights into hypo-expression of the K238R and K412R mutants, we established cell lines stably expressing WT and mutant Flag–Dnmt3L proteins. Cells overexpressing Flag–*Dnmt3L* WT exhibited comparable alkaline phosphatase (AP) staining and proliferation as control and Flag–*Dnmt3L* K238R- and K412R-overexpressing cells, except for a flattened morphology (Supplementary Fig. 5a,b). Consistent with the results obtained upon transient ectopic expression (Fig. 2f), expression of both mutant proteins was remarkably reduced in the stable cell lines (Fig. 4a), despite only slight reductions in their transcription levels (Supplementary Fig. 5c). The hypo-expression of the K238R and K412R mutants persisted during long-term culture and was independent of their mRNA expression levels, further supporting post-transcriptional regulation of Dnmt3L protein stability (Supplementary Fig. 5d,e). Overexpression of these constructs had minimal impact on expression of endogenous *Dnmt3L* and other Dnmts, except that overexpression of Flag–*Dnmt3L* K238R increased the level of the *Dnmt3B* transcript.

The CHX-chase assay revealed that the half-lives of the two mutant proteins were significantly shorter than that of WT Dnmt3L (Fig. 4b). To further validate the physiological relevance of Dnmt3L acetylation, we generated two independent murine ES cell lines carrying endogenous point mutations at the K238 and K412 residues using prime editing and single-stranded oligodeoxynucleotide-mediated homology-directed repair, respectively (Supplementary Fig. 6a–d). For both lines, no off-target sites were identified for at candidate loci identified through in silico prediction permitting up two mismatches. In the case of the K412R line, which was generated via a double-strand break, an extended search permitting up to three mismatches identified eight potential off-target sites, which were confirmed to be unaltered by sequencing (Supplementary Fig. 6e). Using these endogenous mutant systems, CHX-chase assays demonstrated that the half-lives of the endogenous Dnmt3L K238R and K412R proteins were significantly shorter than that of WT Dnmt3L (Supplementary Fig. 6f,g), thereby recapitulating the instability observed in ectopic overexpression models.

These findings prompted us to examine whether the altered protein stabilities of the Dnmt3L mutants were associated with changes in ubiquitination. In mouse ES cells co-expressing Flag–*Dnmt3L* and HA-tagged ubiquitin (HA-Ub), the Flag-IP assay revealed that the K238R and K412R mutants exhibited ubiquitination levels comparable to or slightly higher than that of WT Dnmt3L (Fig. 4c). Quantification analysis indicated that given the reduced expression of these mutants, this finding suggests that the two mutant proteins exhibit higher levels of ubiquitination than WT Dnmt3L, with a more pronounced effect in the K412R mutant. Further analysis showed that K48‑linked polyubiquitin chains, rather than K63‑linked chains, were predominantly detected in the Dnmt3L ubiquitination IP products (Supplementary Fig. 7a,b). Consistent with this, substitution of K48 in HA-tagged ubiquitin markedly reduced Dnmt3L ubiquitination, confirming K48 linkage as the major degradation-associated modification (Supplementary Fig. 7c). These results indicate that acetylation at K238 and K412 protects Dnmt3L from K48-linked ubiquitin-mediated proteasomal degradation. Collectively, these findings indicate that K238Ac or K412Ac plays a critical role in regulating the protein stability of Dnmt3L in mouse ES cells.

Mouse ES cells under 2i-based culture reportedly maintain a naive ground state through G9a- and Prdm14-mediated degradation of de novo Dnmt proteins^24^. On the basis of this previous report, we next explored the potential involvement of G9a and Prdm14 in regulation of Dnmt3L stability. Co-IP assays following cotransfection of mouse ES cells with Flag-tagged *Dnmt3L* (WT, K238R and K412R) and HA-tagged *G9a* (HA-*G9a*) demonstrated robust physical interactions between G9a and all tested Dnmt3L variants (Fig. 4d). These interactions were further validated through reciprocal Co-IP using an anti-HA antibody, indicating that G9a and Dnmt3L directly associate irrespective of the acetylation statuses of K238 and K412.

Importantly, treatment with BIX01294, a G9a-specific inhibitor, significantly increased expression of both exogenous Flag–Dnmt3L and endogenous Dnmt3L proteins (Fig. 4e and Supplementary Fig. 7d), with minimal differences in their transcription levels (Supplementary Fig. 7e). In line with the results observed in long-term 2i-LIF cultures (Fig. 1), induction of Dnmt3L protein by BIX01294 was greater than induction of *Dnmt3L* mRNA, and enhanced protein induction by BIX01294 was notable for the K238R and K412R mutants (Fig. 4f). Accordingly, BIX01294 treatment significantly reduced ubiquitination of both mutant proteins (Fig. 4g, h). In addition, silencing of *Prdm14* increased expression of Dnmt3L (WT, K238R and K412R) proteins (Fig. 4i and Supplementary Fig. 7f), independent of transcriptional induction (Supplementary Fig. 7g). Taken together, these results suggest that the protein stability of Dnmt3L is regulated by a G9a- and Prdm14-dependent degradation pathway, similar to Dnmt3A and Dnmt3B, and that this regulation is critically dependent on K238Ac and K412Ac (Fig. 4j).

### Regulation of naive pluripotency and lineage differentiation genes by Dnmt3L acetylation

**Fig. 5:**
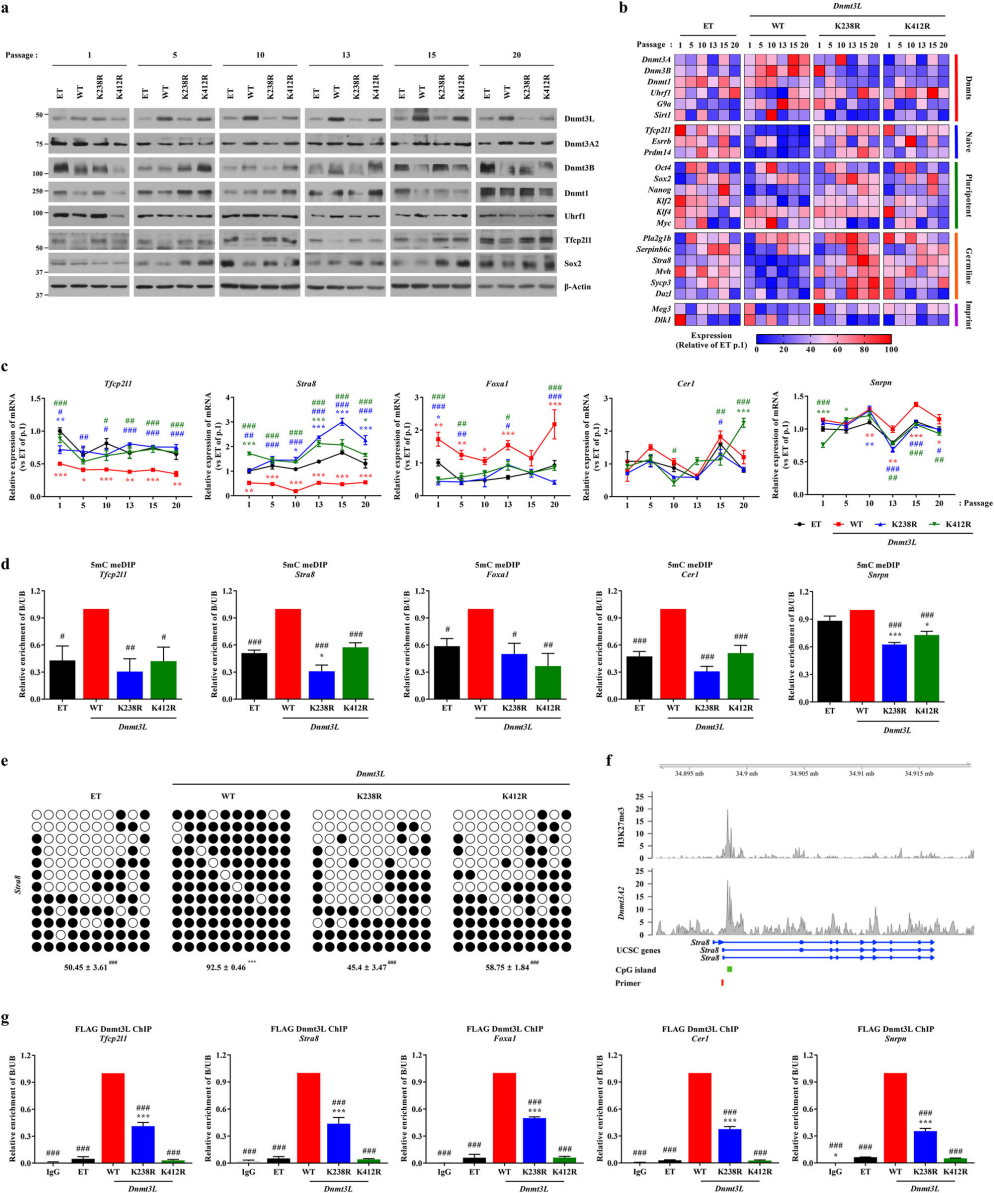
Dnmt3L acetylation regulates naive pluripotency and germline gene expression via DNA methylation. **a** Western blot analysis of DNA methylation-related enzymes and pluripotency factors in long-term cultured mouse ES cells stably expressing nontagged Dnmt3L WT, K238R or K412R proteins. β-actin was used as a loading control. **b** Heat map depicting gene expression profiles of DNA methylation regulators, naive pluripotency genes, germline markers and imprinted genes in the indicated stable mouse ES cell lines. Expression values represent the mean of three independent biological replicates. **c** RT–qPCR analysis of representative genes for naive pluripotency (*Tfcp2l1*), germline (*Stra8*), neural (*Foxa1*) and cardiac (*Cer1*) lineages, and genomic imprinting (*Snrpn*) in cells at the indicated passages (*n* = 3). Expression data for additional genes are shown in Supplementary Fig. 8d. **d** MeDIP–qPCR analysis of 5mC enrichment at the selected genomic loci in stable mouse ES cell lines expressing nontagged WT *Dnmt3L* or mutants (*n* = 4). **e** Bisulfite sequencing analysis of the indicated target regions. Methylated and unmethylated CpG sites are shown as filled and open circles, respectively. The percentage of methylated CpG is indicated below each bisulfite sequencing profile (*n* = 3). **f** Schematic representation of the murine *Stra8* locus, showing the H3K27me3 histone modification mark and Dnmt3A2-binding sites based on published ChIP–seq datasets^26,39^. Genomic features include CpG islands (green bar) and locations of primers used for MeDIP– and ChIP–qPCR assays in this study (red bar). Additional locus maps are shown in Supplementary Fig. 9. **g** ChIP–qPCR analysis of Flag–Dnmt3L WT, K238R and K412R proteins at the regulatory regions of the target genes affected by Dnmt3L K238Ac or K412Ac (*n* = 4). All quantitative data are presented as mean ± s.e.m. Statistical significance was assessed by a one-way ANOVA with the Bonferroni post hoc test; ^*^P < 0.05, ^**^P < 0.01, ^***^P < 0.001 compared with control groups. ^#^*P* < 0.05, ^##^*P* < 0.01, ^###^*P* < 0.001 compared with WT Dnmt3L. The exact *P* values and replicate numbers are provided in the Source Data. See also Supplementary Figs. 8 and 9. ET empty vector, B Bound, UB Unbound.

To investigate the biological role of K238Ac and K412Ac in Dnmt3L, we established another R1 mouse ES cell line that stably expressed nontagged WT *Dntm3L* or the mutants (K238R and K412R) under the control of an exogenous CMV early enhancer/chicken β-actin (CAG) promoter because the CMV promoter used to express Flag–*Dnmt3L* is frequently repressed in mouse ES cells^25^. To minimize irreversible DNA demethylation with massive erasure of genomic imprints upon prolonged 2i-LIF culture^10,11^, the established mouse ES cell lines were propagated in FBS–LIF medium for 20 passages. Mouse ES cells harboring WT *Dnmt3L* formed distinctive flattened colonies displaying weak AP staining (Supplementary Fig. 8a). All tested cells exhibited minimal differences in proliferation capacity, except for a slight increase in growth of *Dnmt3L*-overexpressing cells (Supplementary Fig. 8b). Notably, hypo-expression of the K238R and K412R mutant proteins was recapitulated in this independent stable cell line model and was stably sustained over 20 passages in FBS–LIF culture (Fig. 5a).

To identify the target genes affected by K238Ac and K412Ac of Dnmt3L, we examined changes in expression of genes related to DNA methylation or the developmental process. During long-term culture, overexpression of WT *Dnmt3L* suppressed mRNA expression of naive pluripotency proteins, including *Tfcp2l1* and *Prdm14*; however, this repression was abrogated by the K238 and K412 mutations (Fig. 5b, c). Importantly, repression of *Tfcp2l1* upon *Dnmt3L* overexpression, as well as its restoration observed with the K238R or K412R mutant, was verified at the protein level (Fig. 5a). Although expression of *Oct4*, *Klf2* and *Klf4* was not markedly affected (Supplementary Fig. 8c,d), Sox2 protein expression was suppressed by ectopic expression of WT *Dnmt3L* but not of the K238R or K412R mutant (Fig. 5a).

Previously, we reported that activation of Dnmt3L upon *Sirt1* deficiency represses expression of a subset of imprinted and germline genes concomitant with increased DNA methylation of regulatory elements^5^. In line with this finding, germline developmental genes such as *Stra8*, *Mvh* and *Sycp3* were repressed in ES cells expressing WT *Dnmt3L* during long-term culture (Fig. 5b, c and Supplementary Fig. 8d). Repression of these genes was observed from an early passage (p.5) and sustained until p.20. Importantly, the K238R and K412R mutants protected against the repression of these germline and naive pluripotency genes in mouse ES cells during the entire culture period (Fig. 5b, c), indicating that Dnmt3L K238Ac and K412Ac have long-lasting effects on the expression of these target genes.

To gain mechanistic insight, we assessed the global DNA methylation and demethylation statuses. Dot blot assays revealed minimal changes in the global levels of 5mC, which is a DNA methylation marker, and 5hmC, which is a DNA demethylation marker, regardless of overexpression of WT *Dnmt3L* and the mutants (Supplementary Fig. 8e). Thus, we examined the local DNA methylation statuses of Dnmt3L target genes by performing methylated DNA IP followed by qPCR (meDIP–qPCR). The levels of 5mC in naive pluripotency (*Tfcp2l1*), germline (*Stra8*), neural (*Foxa1* and *Folr1*), cardiac (*Cer1* and *Pkdl1l*) and imprinted (*Snrpn*) genes were increased in WT *Dnmt3L-*expressing mouse ES cells compared with control cells but not in mouse ES cells expressing the K238R or K412R mutant (Fig. 5d and Supplementary Fig. 8f). This finding was validated by a bisulfite conversion-based assay (Fig. 5e).

On the basis of a previous chromatin IP followed by sequencing (ChIP–seq) result^26^ showing that Dnmt3A2 binds at loci sensitive to Dnmt3L K238Ac or K412Ac (Fig. 5f and Supplementary Fig. 9), we examined the occupancy of WT Dnmt3L and mutant proteins at these regions using ChIP–qPCR. Consistent with the gene expression patterns, WT Dnmt3L was enriched at regulatory elements of the previously described target genes, whereas the K238R and K412R mutants showed markedly reduced binding at these loci (Fig. 5g and Supplementary Fig. 8g). Taken together, these results indicate that transcriptional repression of a subset of genes related to naive pluripotency and germline and somatic (neural and cardiac lineages) development by Dnmt3L is dependent on K238Ac and K412Ac.

### Role of Dnmt3L K238Ac and K412Ac in the developmental potential of mouse ES cells

**Fig. 6:**
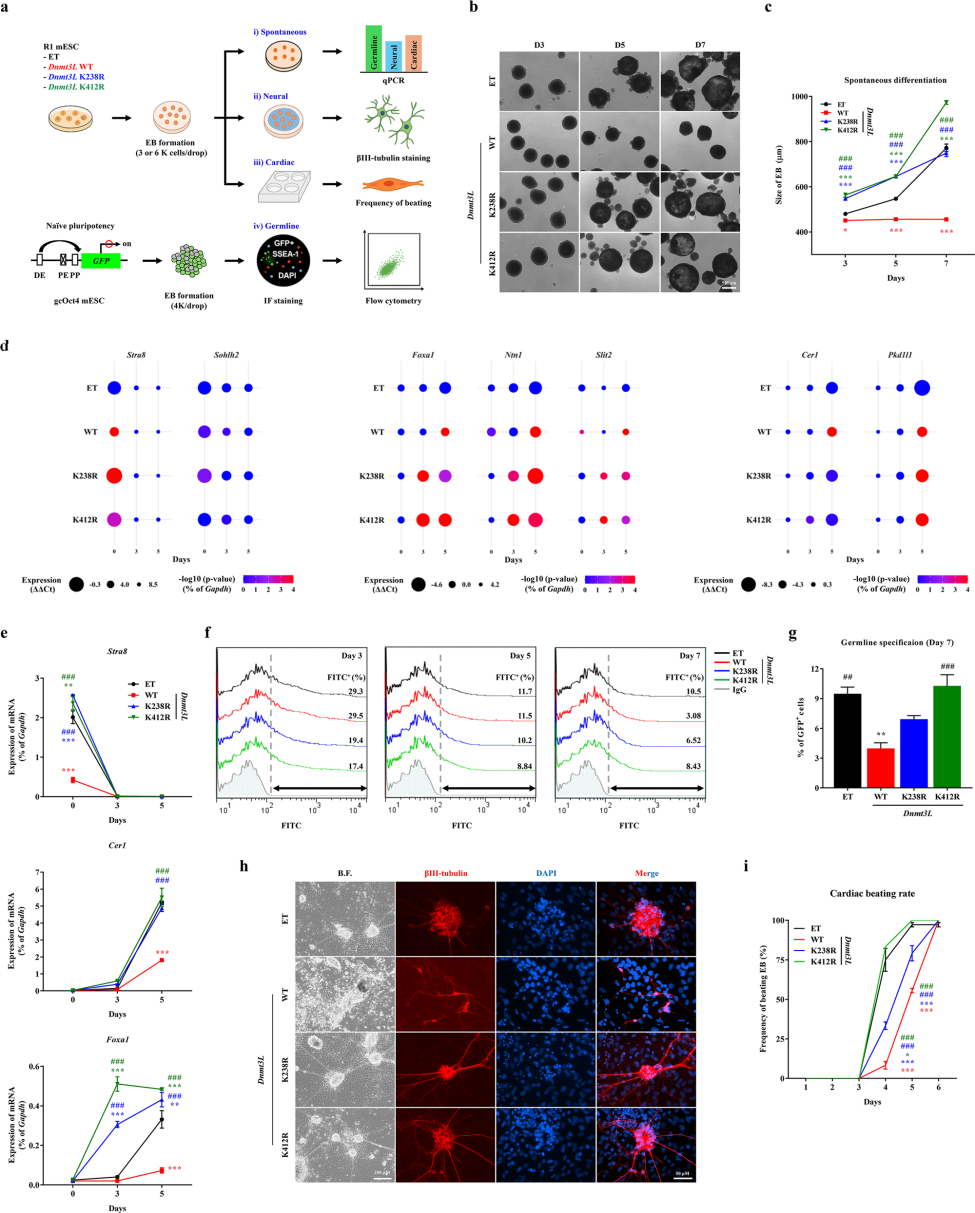
Dnmt3L K238Ac and K412Ac regulate the in vitro differentiation capacity of mouse ES cells across multiple lineages. **a** Schematic overview of in vitro differentiation assays to assess lineage-specific outcomes in spontaneous, neural, cardiac and germline contexts. **b** Representative phase-contrast images of EBs generated from mouse ES cells stably expressing nontagged *Dnmt3L* WT, K238R and K412R at the indicated time points. Images were captured at 40× magnification. Scale bar, 500 µm. **c** Quantification of EB size during spontaneous differentiation. WT *Dnmt3L* EBs exhibited significantly reduced growth compared with EBs derived from control and mutant lines (*n* = 10). **d** Bubble plot summarizing qPCR analysis of differentiation marker genes associated with germline, neural and cardiac lineages in EBs. Relative expression and statistical significance (*Gapdh*-normalized) were calculated from three biologically independent replicates. **e** RT–qPCR analysis of the representative markers *Stra8* (germline), *Cer1* (cardiac) and *Foxa1* (neural) during EB differentiation (*n* = 3). Additional expression profiles are shown in Supplementary Fig. 10b. **f**, **g** Flow cytometric analysis of GFP^+^ germ cells among gcOct4 ES cells at the indicated time points after EB formation (**f**). The proportion of GFP^+^ cells is displayed in the corresponding histograms (**g**). Quantification of GFP^+^ germ cells among gcOct4-derived EBs at day 7 after induction based on flow cytometric analysis (*n* = 3). **h** IF staining images showing βIII-tubulin^+^ neuronal cells (red) following directed neural differentiation. Nuclei were counterstained with 4′,6-diamino-2-phenylindole (DAPI) (blue). Images were captured at 400× magnification. Scale bar, 50 µm. **i** Time-course quantification of beating EBs from day 1 to 6 during cardiac differentiation. The onset of beating was delayed for WT *Dnmt3L*-expressing cells compared with control and acetylation-deficient mutants (*n* = 3). All quantitative data are presented as mean ± s.e.m. Statistical significance was determined using a two-way (**c**, **e** and **i**) or one-way (**g**) ANOVA with the Bonferroni post hoc test. ^*^P < 0.05, ^**^P < 0.01, ^***^P < 0.001 compared with control groups. ^#^*P* < 0.05, ^##^*P* < 0.01, ^###^*P* < 0.001 compared with WT Dnmt3L. See also Supplementary Fig. 10. BF Bright field.

To evaluate the functional significance of Dnmt3L K238Ac and K412Ac in lineage commitment, we induced spontaneous differentiation of mouse ES cells through embryoid body (EB) formation, which recapitulates key aspects of early embryonic development (Fig. 6a and Supplementary Fig. 10a). EBs derived from WT *Dnmt3L*-expressing cells (WT *Dnmt3L* EBs) displayed impaired morphogenesis, which was characterized by defective formation of an endoderm-like hollow structure (Fig. 6b) and reduced size (Fig. 6c). Gene expression analysis revealed that induction of lineage-specific genes associated with germline (for example, *Stra8* and *Sohlh2*), neural (for example, *Foxa1*, *Ntn1* and *Slit2*) and cardiac (for example, *Cer1* and *Pkd1l1*) differentiation was markedly delayed in WT *Dnmt3L* EBs (Fig. 6d, e and Supplementary Fig. 10b). Notably, these defects were absent in EBs derived from cells expressing the control vector or the K238R or K412R mutant, highlighting the critical role of site-specific acetylation of Dnmt3L in proper lineage differentiation of mouse ES cells.

Among Dnmt3L K238Ac- and K412Ac-dependent target genes, *Tfcp2l1* stimulates naive and somatic reprogramming^3^. Therefore, to investigate the role of induction of Dnmt3L and its K238Ac or K412Ac in the generation of iPS cells, we used mouse embryonic fibroblasts obtained from a mouse model harboring four reprogramming factors (OSKM) under the control of the tet-on promoter (iOSKM) mouse embryonic fibroblasts^27^. Expression of WT Dnmt3L or mutant proteins had a minimal effect on the number of AP^+^ iPS cell colonies (Supplementary Fig. 10c), suggesting that Dnmt3L K238Ac and K412Ac have little influence on the somatic cell reprogramming process.

Given the repressive effect of WT *Dnmt3L* on germline gene expression, we next assessed the role of K238Ac and K412Ac in germ cell differentiation using gcOct4 mouse ES cells (Fig. 6a), which express green fluorescent protein (GFP) under the control of an Oct4 promoter lacking the proximal enhancer, thereby restricting GFP expression to germ cells during development^28^. Following EB formation, GFP^+^ primordial germ cells were detected in all groups at early stages (days 3 and 5). However, at day 7, induction of primordial germ cell colonies was markedly reduced in WT *Dnmt3L* EBs compared with those expressing the control or mutant (K238R or K412R) constructs, as demonstrated by both flow cytometry (Fig. 6f, g) and IF analysis (Supplementary Fig. 10d).

Next, we compared the in vitro neuronal and cardiac differentiation potentials of mouse ES cells expressing WT *Dnmt3L* or the K238R or K412R mutant. In line with the gene expression results (Fig. 6d), WT *Dnmt3L*-expressing ES cells exhibited severe defects in neuronal differentiation in vitro, as evidenced by a lack of βIII-tubulin^+^ neurons (Fig. 6h) and induction of neuronal markers (Supplementary Fig. 10e). However, ES cells expressing either mutant gave rise to βIII-tubulin^+^ neurons, similar to control cells, and displayed similar gene expression patterns.

During in vitro cardiac differentiation, the time to initiation of beating was delayed in WT *Dnmt3L*-expressing ES cells compared with the other groups (Fig. 6i). Morphologically, the cell mass generated following cardiac differentiation of WT *Dnmt3L*-expressing ES cells had a flattened structure, unlike other cells with a round morphology and a hollowed compartment (Supplementary Movie 1). These retarded cardiac differentiation phenotypes were not observed with mouse ES cells expressing the control vector or the K238R or K412R mutant. Collectively, these results demonstrated that Dnmt3L K238Ac or K412Ac plays a crucial role in regulation of differentiation processes, including differentiation into germline, neural and cardiac lineages.

### The in vivo differentiation potential of mouse ES cells is regulated by Dnmt3L K238Ac and K412Ac

**Fig. 7:**
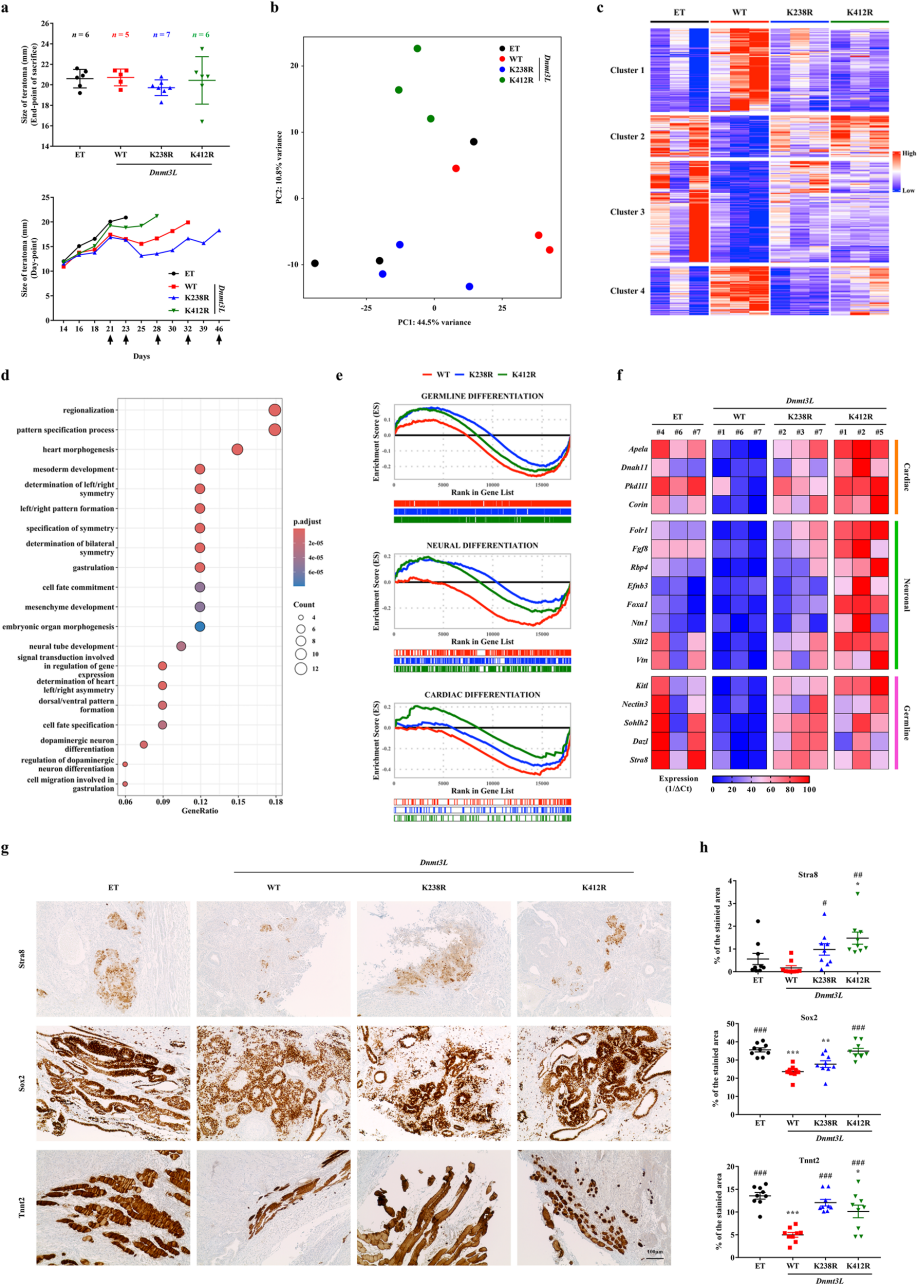
In vivo relevance of Dnmt3L K238Ac and K412Ac in regulation of lineage-specific differentiation of mouse ES cells. **a** Time-course changes in teratoma size (top) and final teratoma size quantification (bottom) for 46 days after subcutaneous injection of mouse ES cells stably expressing the ET or the *Dnmt3L* WT, K238R or K412R construct into NOD-SCID mice (*n* = 7 per group). Arrows indicate the day of death. **b** Principal component analysis of global transcriptomes in collected teratomas. Samples clustered distinctly on the basis of Dnmt3L acetylation status, with high intragroup consistency. **c** Clustering of DEGs across all groups identified four expression modules. Clusters 2 and 3 were selectively downregulated in WT *Dnmt3L* teratomas but their expression was restored in teratomas derived from mouse ES cells expressing the K238R and K412R mutants. **d** GO enrichment analysis of cluster 2 revealed substantial enrichment of pathways related to neural and cardiac development. **e** GSEA demonstrated notable recovery of lineage-specific gene programs, including cardiac, neural and germline differentiation signatures, in teratomas derived from mouse ES cells expressing the K238R and K412R mutants relative to WT *Dnmt3L* teratomas. **f** Heat map summarizing qPCR analysis of selected DEGs involved in lineage commitment, showing transcriptional repression in WT *Dnmt3L* teratomas and restoration of expression in teratomas derived from mouse ES cells expressing the acetylation-deficient mutants (*n* = 4). **g** IHC staining of lineage markers in teratoma sections at day 21 after injection. WT *Dnmt3L* teratomas exhibited reduced numbers of Stra8^+^ (germ cells), Sox2^+^ (neurons) and Tnnt2^+^ (cardiac) cells compared with the control and mutant groups. Images were captured at 200× magnification. Scale bar, 100 µm. **h** Quantification of positive cell frequencies from IHC staining of the indicated teratoma sections (*n* = 9), confirming that the in vivo differentiation potential of WT *Dnmt3L*-expressing mouse ES cells was impaired. All quantitative data are presented as mean ± s.e.m. Statistical comparisons were performed using a two-way ANOVA with the Bonferroni post hoc test. ^*^P < 0.05, ^**^P < 0.01, ^***^P < 0.001 versus ET control group; ^#^*P* < 0.05, ^##^*P* < 0.01, ^###^*P* < 0.001 versus WT *Dnmt3L*. See also Supplementary Figs. 11–13.

To validate these findings in vivo, we assessed the developmental potential of mouse ES cells expressing WT *Dnmt3L* or the acetylation-deficient mutants (K238R and K412R) through subcutaneous teratoma formation in immunodeficient NOD-SCID mice. All mouse ES cell lines successfully generated teratomas containing derivatives of all three germ layers, irrespective of the Dnmt3L status (Supplementary Fig. 11a,b). Although teratomas derived from WT *Dnmt3L*- and K238R-expressing mouse ES cells exhibited slightly delayed growth kinetics, final tumor sizes were comparable in all groups (Fig. 7a).

Histopathological analysis revealed that mesodermal and endodermal lineage differentiation was markedly reduced in teratomas derived from WT *Dnmt3L*-expressing mouse ES cells (WT *Dnmt3L* teratomas) compared with those derived from mouse ES cells expressing the control vector (Supplementary Fig. 11c). To further investigate this phenotype, we performed transcriptomic profiling of the teratomas. With the exception of one outlier in the control group, each group showed high intragroup consistency and clear intergroup differences (Fig. 7b). To investigate the overall differences among groups, we conducted Gene Ontology (GO) analysis comparing the WT *Dnmt3L*-overexpressing group with the control and mutant groups using the differentially expressed genes (DEGs) that showed at least a twofold change and a *P* value <0.05 (Supplementary Fig. 12a). The top 20 GO terms from each comparison were visualized as a Venn diagram to identify overlapping biological processes. Notably, little common enriched GO terms were observed between the WT versus control and WT versus mutant comparisons (Supplementary Fig. 12b). This finding suggests that the gene expression changes induced by *Dnmt3L* overexpression are diminished when the acetylation sites of Dnmt3L are mutated. Furthermore, GO terms exclusively represented in the WT group (versus control) were associated with meiosis, a process essential for germline differentiation.

Furthermore, DEG analysis identified four expression clusters, among which clusters 2 and 3 were substantially downregulated in WT *Dnmt3L* teratomas but restored in those expressing the K238R or K412R mutant (Fig. 7c). GO analysis of these clusters revealed enrichment of terms related to neural, cardiac and germline development (Fig. 7d and Supplementary Fig. 13a), recapitulating the lineage-specific defects observed in in vitro differentiation assays (Fig. 6). Gene set enrichment analysis (GSEA) further demonstrated that expression of gene sets downregulated by WT *Dnmt3L* overexpression, specifically those involved in germline, neural and cardiac differentiation, was notably restored in teratomas expressing the K238R or K412R mutant (Fig. 7e). These findings were supported by direct gene expression analysis. Genes involved in these developmental processes were repressed in WT *Dnmt3L* teratomas but not in those expressing the K238R or K412R mutant (Fig. 7f and Supplementary Fig. 13b). Immunohistochemistry (IHC) staining at day 21 after injection confirmed that the levels of lineage-specific markers—including Sox2^+^, NeuN^+^ or βIII-tubulin^+^ neural cells; Tnnt2^+^, Tnni3^+^ or Bmp4^+^ cardiac cells; and Stra8^+^ germ cells—were lower in WT *Dnmt3L* teratomas than in those expressing the control vector and the mutants (Fig. 7g, h and Supplementary Fig. 13c,d). Collectively, these findings establish that Dnmt3L K238Ac and K412Ac are critical for modulating the differentiation of mouse ES cells in vivo, particularly toward neural, cardiac and germline lineages.

## Discussion

The appropriate epigenetic state is crucial for preserving pluripotency and guiding the differentiation of PS cells. This study demonstrates that culture conditions, such as 2i-LIF, dynamically modify Dnmt3L expression through post-transcriptional mechanisms. Acetylation at critical lysine residues, such as K238 and K412, impacts the stability and function of Dnmt3L and thereby affects DNA methylation patterns and the differentiation potential of mouse ES cells, particularly toward germline, neural and cardiac lineages.

Long-term culture of mouse ES cells under 2i-LIF conditions, which are characterized by inhibition of the Mek1/2 and Gsk3 pathways, has been extensively used to maintain a naive pluripotent state. However, these conditions pose significant challenges, particularly in sustaining DNA methylation stability over prolonged periods. Extended 2i-LIF culture can lead to considerable loss of genomic imprints and alterations in DNA methylation patterns, impairing the developmental potential of ES cells^10,11,19^. This DNA hypomethylation is particularly problematic because it can cause irreversible changes of the epigenetic landscape, particularly at developmentally crucial genes such as genomic imprints, necessitating the identification of biomarkers that represent the state of DNA methylation stability and help optimize culture conditions to maintain epigenetic integrity. In this study, we observed that under long-term 2i-LIF culture, in contrast to the stable suppression of maintenance (Dnmt1) and de novo (Dnmt3A and Dnmt3B) Dnmts, Dnmt3L was dynamically induced at the post-transcriptional level from early passages. A rebound of *Dnmt3L* transcript expression was observed in several mouse ES cell lines during their adaptation from serum-containing to 2i-LIF cultures^29^. Notably, when cells were re-exposed to serum-containing medium, Dnmt3L was remarkably upregulated compared with other Dnmts. These findings suggest that Dnmt3L plays a critical role in fine-tuning DNA methylation under different culture conditions.

Notably, although Dnmt3L functions as an obligate cofactor for Dnmt3A and Dnmt3B, our findings reveal that it displays uniquely dynamic regulation in response to changing the culture environments. Unlike Dnmt3A, whose transcription is repressed under prolonged 2i-LIF culture, Dnmt3L protein levels exhibit rapid post-transcriptional induction and reactivation, particularly upon re-exposure to FBS-based conditions. This dynamic pattern of Dnmt3L expression is reminiscent of Uhrf1, another epigenetic cofactor whose protein stability modulates maintenance methylation in response to developmental signals^30^. Previous studies have highlighted the essential roles of Dnmt3A–Dnmt3L complexes in the epigenetic adaptation of ES cells^31^. However, our data suggest that Dnmt3L is not merely a passive partner but may act as a dynamic sensor of extracellular environment, whose PTM such as acetylation regulate its protein stability and chromatin binding independently of de novo Dnmt levels. This provides new insight into how cofactors such as Dnmt3L serve as regulatory nodes connecting environmental signals to epigenetic remodeling in PS cells.

In 2i-LIF culture, global DNA hypomethylation is driven by multiple mechanisms, including regulation of Dnmts and Tet-dependent DNA demethylation as well as modulation of histone modifications. In mouse ES cells under serum-containing culture conditions, Uhrf1 binds to hemi-methylated DNA and H3K9me2 to target replication foci; however, in 2i-LIF culture, DNA methylation is lost owing to reduced Uhrf1 levels and loss of H3K9me2, which impair its recruitment^30^. Notably, regions with increased DNA methylation in 2i-LIF culture are closely associated with the presence of H3K9me3 on endogenous retroviruses and imprinted loci^29^. In addition, hypomethylation may be mediated by suppression of Dnmt3A or Dnmt3L under 2i-LIF conditions^31^. In this context, Dnmt3L may play a pivotal role as a modulator of de novo DNA methylation by stabilizing Dnmt3A^32^, indicating that its proper regulation is essential to counteract the DNA methylation erosion observed in 2i-LIF cultures. Therefore, a further study is required to investigate the interplay between Dnmt3L and other epigenetic factors for orchestrating the epigenetic stability of mouse ES cells under diverse culture conditions.

The activities and stabilities of many epigenetic factors are regulated by PTM-dependent mechanisms. For instance, the stability of DNMT1 is controlled by acetylation of its KG linker, which disrupts the association between DNMT1 and USP7, leading to proteasomal degradation of DNMT1^33^. Conversely, HAUSP and HDAC1 protect Dnmt1 from degradation by promoting deubiquitination and deacetylation, respectively^34^. In maintenance of the naive ground state of mouse ES cells, Prdm14-mediated formation of the G9a–Dnmt3a complex facilitates Dnmt3A protein degradation via methylation of the PWWP domain in Dnmt3A and Dnmt3B by G9a^24^. In our previous study, we reported that acetylation of Dnmt3L is increased in *Sirt1*-deficient mouse ES cells, which exhibit abnormal DNA methylation in a subset of imprinted and germline developmental genes, ultimately leading to impaired developmental potency^5^.

In this context, our identification of 17 acetylation sites and 24 phosphorylation sites could provide the basis of the PTM profile for regulation of Dnmt3L. Although we did not pinpoint a single acetylation site responsible for the marked decrease in acetylation levels, we identified K238 and K412 as key acetylation sites influencing protein stability. Acetylation at these sites regulated Dnmt3L protein stability, which in turn affected expression of genes involved in germline, cardiac and neural lineages, thereby influencing the developmental potency of mouse ES cells. Consequently, future research should focus on identifying the histone methyltransferases or HDACs that specifically regulate K238Ac and K412Ac and further elucidating their functional roles. As a mechanistic basis for the hypo-expression of Dnmt3L K238R and K412R mutants, our data demonstrate that Dnmt3L protein stability is regulated through a G9a- and Prdm14-dependent ubiquitin-mediated degradation pathway, consistent with previously reported mechanisms for Dnmt3A and Dnmt3B regulation^24^. In silico analysis using BioGRID^35^ and UbiBrowser 3.0^36^ identified several potential Dnmt3L-interacting E3 ligases, including components of Polycomb repressive complexes such as Eed, Bmi1 and Ring1. These findings suggest that Dnmt3L degradation may be modulated through Polycomb repressive complex-associated ubiquitination, highlighting an unexplored regulatory axis connecting epigenetic silencing complexes and Dnmt3L protein stability. Further investigation into the functional relevance of these interactions will be critical for elucidating the upstream control of Dnmt3L turnover.

It should be noted that Dnmt3L has a dual role in mouse ES cells: it acts not only as a positive regulator of DNA methylation at gene bodies but also as a negative regulator at bivalent promoters, which are key regions that remain poised for activation during differentiation^32,37^. This dual function highlights the intricate role of Dnmt3L to precisely modulate the DNA methylation landscape, balancing the maintenance of pluripotency with the commitment to differentiation pathways. Consistent with our previous finding regarding Dnmt3L activation due to *Sirt1* deficiency^5^, *Dnmt*3L overexpression repressed genes related to naive pluripotency, the germline fate and developmental processes, which significantly impaired cardiac and neural differentiation, as demonstrated in both in vitro and in vivo differentiation models. In particular, we observed distinct alterations of DNA methylation and expression of genes related to these affected lineages using mouse ES cells expressing WT *Dnmt3L* and the K238R and K412R mutants and an in vitro EB differentiation model. These findings were further validated through transcriptome analysis of teratomas generated by these mouse ES cells.

Interestingly, Dnmt3L appears to play a crucial role in neural differentiation by promoting phosphorylation of STAT1 and STAT3, independent of its role in DNA methylation, which potentially contributes to the atypical phenotypes observed in the cortex of individuals with Down syndrome^38^. This suggests that Dnmt3L participates in a broader range of epigenetic and signaling pathways than previously appreciated and influences cell fate decisions through both DNA methylation-dependent and -independent mechanisms. Therefore, future studies should focus on identifying the specific targets of Dnmt3L in the regulation of neural and cardiac development and elucidating the precise mode of action as well as investigating diseases linked to these pathways.

In conclusion, our study offers a detailed insight into the role of Dnmt3L as a crucial regulator of the epigenetic landscape in mouse ES cells in various culture conditions, where it regulates both the maintenance of pluripotency and progression toward differentiation. We demonstrated that acetylation of K238 and K412 is a key mechanism regulating Dnmt3L protein stability. It is essential to investigate whether these findings in mouse ES cells are applicable to human ES cells and iPS cells to increase the significance of Dnmt3L in stem cell biology and regenerative medicine, especially for refining culture conditions to preserve the epigenetic stability of PS cells.

## Supplementary information

Supplementary Information

Supplementary Movie 1

## Source data

Source data

## Data Availability

The mass spectrometry proteomics data generated in this study have been deposited in the PRIDE Archive under accession no. PXD032953 (https://www.ebi.ac.uk/pride/archive/projects/PXD032953). ChIP–seq datasets for Dnmt3L^26^ and H3K27me3^39^ in mouse ES cells are publicly available from the NCBI Gene Expression Omnibus under accession nos. https://www.ncbi.nlm.nih.gov/geo/query/acc.cgi?acc=GSE57413 and https://www.ncbi.nlm.nih.gov/geo/query/acc.cgi?acc=GSE40065, respectively. The bulk RNA-seq transcriptome data generated in this study are available in GEO under accession no. https://www.ncbi.nlm.nih.gov/geo/query/acc.cgi?acc=GSE298928. The software and their versions we used for transcriptome analysis are provided in the Source Data. R scripts to analyze the transcriptome and generate plots are freely available upon request. The ‘TIF to grayscale image converter’ used to generate quantitative data from the IHC staining images in this study is available via GitHub at https://github.com/chfhrqlc/GrayScale_Converter.git.
