# Supplementary material for: Uncovering the acetylation sites of Dnmt3L that regulate protein stability and differentiation potency in embryonic stem cells
Source: Exp Mol Med. 2026 Mar 4;58(3):709–24. doi: 10.1038/s12276-026-01655-w (PMC13049025; doi:10.1038/s12276-026-01655-w)
Supplement: Supplementary file 1 — Supplementary Information [file 12276_2026_1655_MOESM1_ESM.pdf]

# SUPPLEMENTARY INFORMATION

**Uncovering the Acetylation Sites of Dnmt3L That Regulate Protein Stability and Differentiation Potency in Embryonic Stem Cells**

Corresponding authors: naiad0226@ulsan.ac.kr, d0shin03@amc.seoul.kr, and kimkyunggon@gmail.com

**This PDF file includes:**

Supplementary Fig. 1 to 13 and figure legends

Supplementary Movie 1 legend

Supplementary Materials and Methods

Key resource table

Uncropped WB

Supplementary References

**Other Supplementary Information includes two separate files including**

**Videoclip for Supplementary Movie 1**

**Source datasets.** Source data for quantification analyses

# SUPPLEMENTARY FIGURE LEGENDS

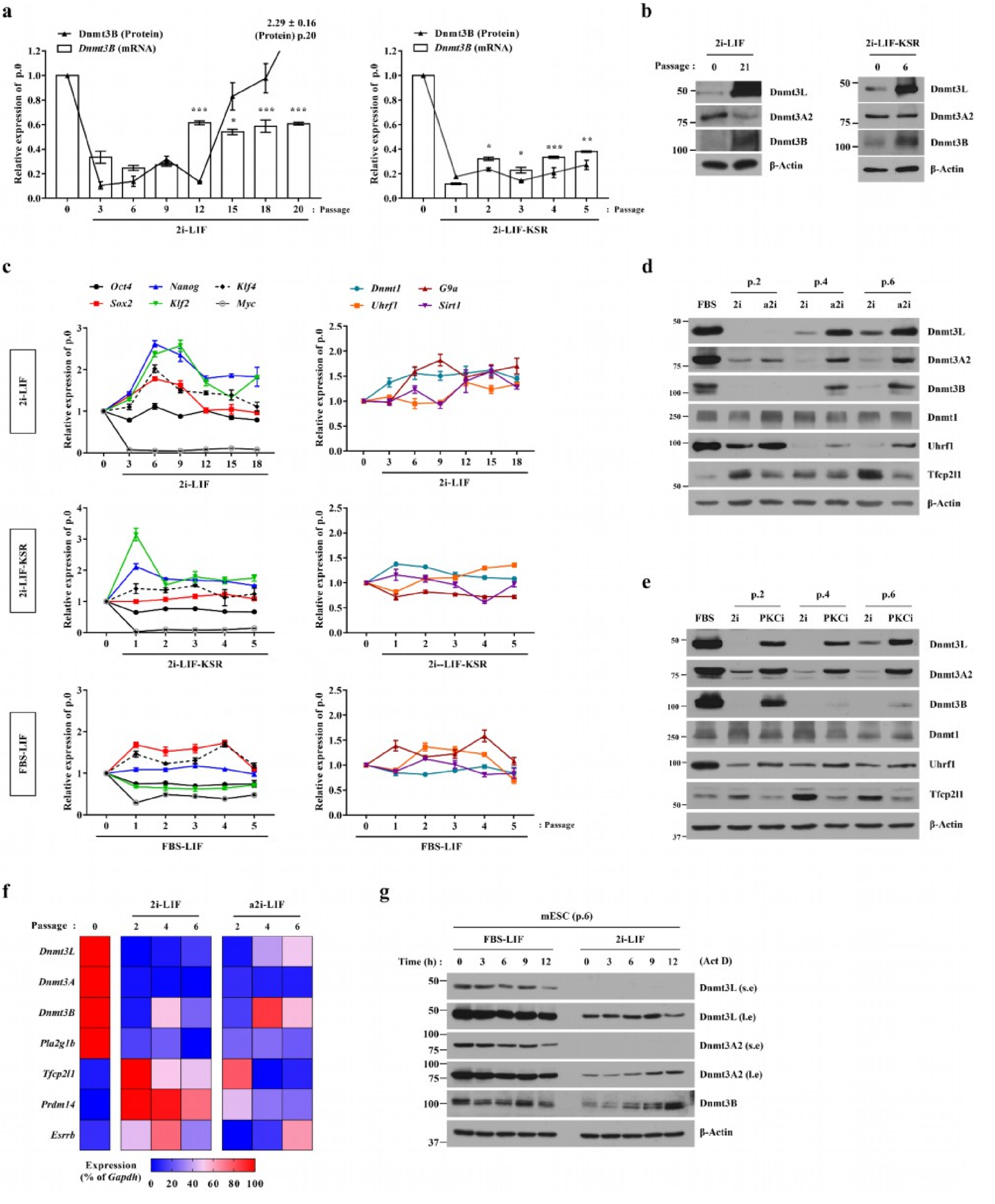

**Supplementary Fig. 1. Culture condition-specific modulation of Dnmt3L expression in mESCs**

**(a)** Temporal expression profiles of Dnmt3B at the mRNA (bar) and protein (solid line) levels during extended propagation under 2i-LIF (left panel) and 2i-LIF-KSR (right panel) culture conditions ($n$ = 4). Outlier values falling outside the plotted range are indicated as numbers above the corresponding passages. **(b)** Western blot analysis of Dnmt3L and other *de novo* Dnmts in mESCs at p.0 and after re-exposure to FBS-LIF medium following prolonged 2i-LIF or 2i-LIF-KSR culture. Molecular weight markers (kDa) are indicated on the left. β-Actin served as a loading control. **(c)** RT-qPCR analysis of genes associated with pluripotency (left panel) and DNA methylation (right panel) under three distinct propagation conditions: 2i-LIF, 2i-LIF-KSR, and FBS-LIF ($n$ = 4). **(d and e)** Western blot analyses showing expression of Tfcp2l1 and DNA methylation-related proteins in mESCs cultured under modified 2i conditions, such as a2i-LIF and PKCi-LIF, compared with conventional 2i-LIF culture. **(f)** Heatmap illustrating differential expression of genes associated with DNA methylation and naïve pluripotency between 2i-LIF and a2i-LIF conditions. Data represent mean values from three independent biological replicates. **(g)** Western blot analysis of Dnmt3L and Dnmt3A2 proteins following actinomycin D (ActD) treatment (7.5 μg/mL) to block *de novo* transcription in p.6 mESCs maintained under 2i-LIF culture. Short (s.e) and long (l.e) exposures are shown. Quantitative data are represented as mean ± SEM. Statistical analysis was performed using a two-way ANOVA with the Bonferroni post hoc test. *$p < 0.05$, **$p < 0.01$, ***$p < 0.001$ compared with control groups. Exact p-values and replicate numbers are provided in Source datasets.

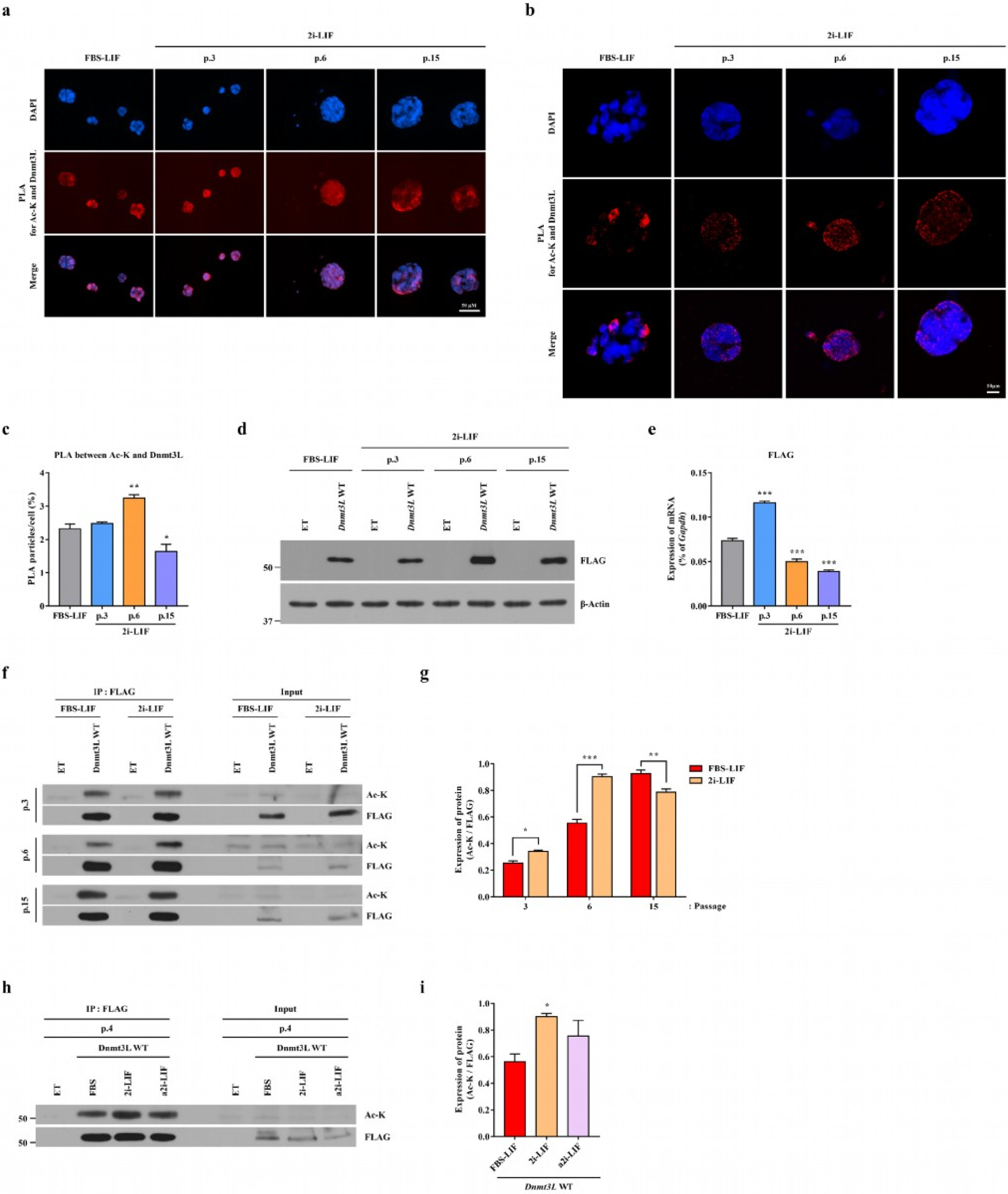
a
2i-LIF
FBS-LIF
p.3
p.6
p.15
DAPI
PLA
for Ac-K and Dnmt3L
Merge
50 μM
b
2i-LIF
FBS-LIF
p.3
p.6
p.15
DAPI
PLA
for Ac-K and Dnmt3L
Merge
c
PLA between Ac-K and Dnmt3L
PLA particles/cell (%)
FBS-LIF
p.3
p.6
p.15
2i-LIF
d
2i-LIF
FBS-LIF
p.3
p.6
p.15
ET
Dnmt3L WT
FLAG
β-Actin
50
37
e
FLAG
Expression of mRNA
(% of Gapdh)
FBS-LIF
p.3
p.6
p.15
2i-LIF
f
IP : FLAG
Input
FBS-LIF
2i-LIF
ET
Dnmt3L WT
p.3
p.6
p.15
Ac-K
FLAG
g
FBS-LIF
2i-LIF
Expression of protein
(Ac-K / FLAG)
: Passage
h
IP : FLAG
Input
p.4
Dnmt3L WT
ET
FBS
2i-LIF
a2i-LIF
Ac-K
FLAG
i
Expression of protein
(Ac-K / FLAG)
FBS-LIF
2i-LIF
a2i-LIF
Dnmt3L WT

**Supplementary Fig. 2. Culture condition–dependent modulation of Dnmt3L acetylation in mESCs**

**(a and b)** Representative fluorescence and confocal images of proximity ligation assay (PLA) signals detecting acetylated Dnmt3L in R1 mESCs cultured under prolonged 2i-LIF or FBS-LIF conditions. Images were acquired at ´400 **(a)** and ´1000 **(b)** magnification. Scale bar, 50 µm **(a)** and 10 µm **(b)**. **(c)** Quantification of PLA signals representing acetylated Dnmt3L ($n$ = 3). **(d and e)** Immunoblotting **(d)** and RT-qPCR **(e)** analyses of FLAG-tagged exogenous *Dnmt3L* expression in mESCs cultured under 2i-LIF and FBS-LIF conditions. Transcript levels were normalized to *Gapdh* ($n$ = 4). **(f)** FLAG-immunoprecipitation (IP) followed by immunoblotting for acetyl-lysine (Ac-K) in mESCs stably expressing FLAG-*Dnmt3L*, showing temporal changes in acetylation at early (p.3), intermediate (p.6), and late (p.15) passages under 2i-LIF or FBS-LIF. **(g)** Quantification of acetylated Dnmt3L levels normalized to FLAG-Dnmt3L in the IP fractions ($n$ = 3). **(h)** FLAG-IP followed by immunoblot analysis of FLAG-Dnmt3L acetylation in mESCs cultured for six passages under FBS-LIF, conventional 2i-LIF, and modified 2i conditions (a2i-LIF). **(i)** Quantification of acetylated FLAG-Dnmt3L levels, normalized to total FLAG-Dnmt3L in the IP fractions ($n$ = 4). Quantitative values are shown as means ± SEM. *$p < 0.05$, **$p < 0.01$, ***$p < 0.001$ compared with the FBS-LIF group by a one-way ANOVA **(c, e and i)** and two-way ANOVA **(g)** with the Bonferroni post hoc test.

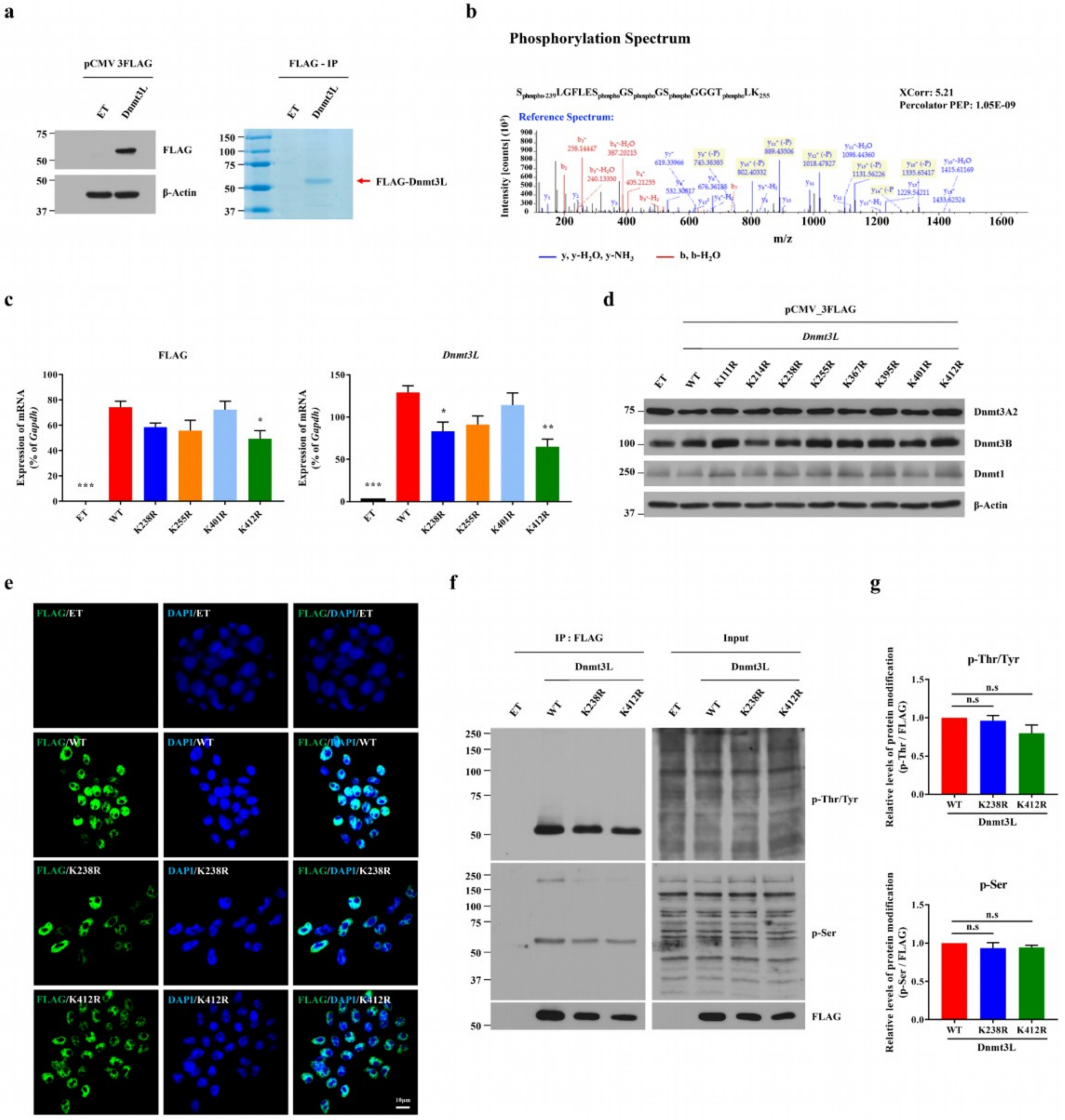


**Supplementary Fig. 3. Biochemical characterization of Dnmt3L lysine mutants**

**(a)** Western blot (left panel) and Coomassie blue staining (right panel) of FLAG-IP products

from R1 mESCs ectopically expressing FLAG-*Dnmt3L*. β-Actin was used as a loading control. **(b)** Representative MS/MS spectra showing phosphorylated peptides of Dnmt3L. Red and blue lines denote b and y ions, respectively. **(c)** RT-qPCR analysis of exogenous (left panel) and endogenous (right panel) *Dnmt3L* transcripts normalized to *Gapdh* in mESCs expressing WT *Dnmt3L* or mutant constructs ($n$ = 3). **(d)** Western blot analysis of endogenous Dnmt3A2, Dnmt3B, and Dnmt1 proteins in mESCs expressing FLAG-tagged *Dnmt3L* WT or K→R mutants. **(e)** Immunocytochemistry staining of FLAG-Dnmt3L WT, K238R, and K412R proteins in mESCs. Nuclei were counterstained with DAPI (blue). Images were acquired at ×1,000 magnification. Scale bar = 10 μm. **(f)** Western blot analysis of phosphorylation at threonine/tyrosine (p-Thr/Tyr; upper panel) and serine (p-Ser; middle panel) residues in FLAG-IP samples of Dnmt3L WT, K238R, and K412R mutant proteins. Input lanes represent 20% of total cell lysates used for the IP assay. **(g)** Quantification of p-Thr/Tyr and p-Ser levels in IP fractions, normalized to total FLAG-Dnmt3L protein ($n$ = 3). Reduced phosphorylation levels of the mutants likely reflect their decreased protein abundance (lower panel) rather than site-specific effects. All quantitative data are presented as mean ± SEM. Statistical significance was evaluated using a one-way ANOVA with the Bonferroni post hoc test. *$p < 0.05$, **$p < 0.01$, ***$p < 0.001$ compared with the WT Dnmt3L group. n.s., non-significant.

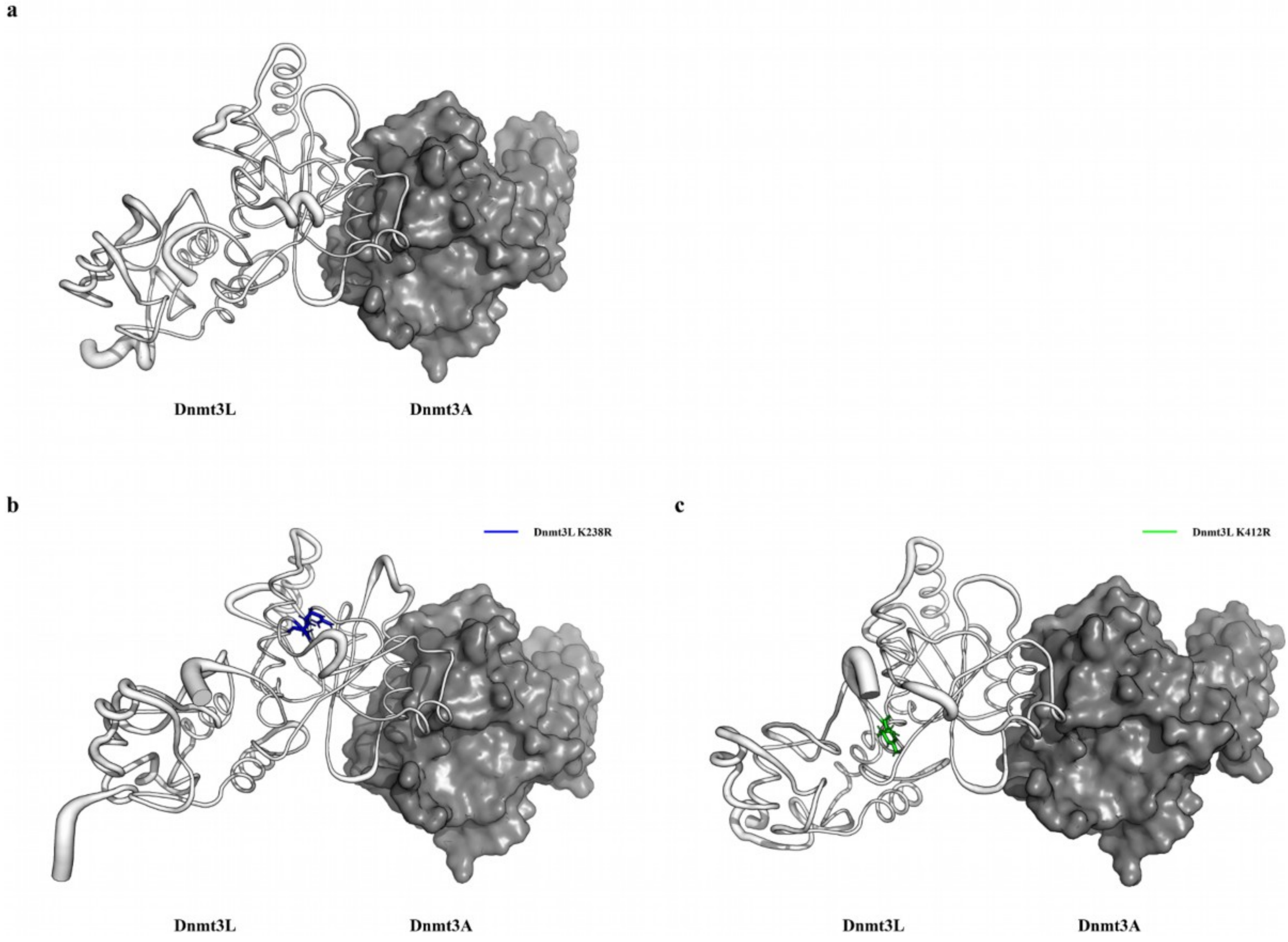


**Supplementary Fig. 4. Structural flexibility profiles of WT Dnmt3L and acetylation-deficient mutants**

**(a–c)** Visualization of residue-specific structural flexibility in Dnmt3L WT **(a)**, K238R **(b)**, and K412R **(c)** proteins based on RMSF values derived from MD simulations. The thickness of the protein backbone representation reflects local flexibility, with thicker segments indicating regions of higher RMSF. Dnmt3A, the known binding partner, is shown as a gray surface to mark the interaction interface. Acetylation-deficient mutation sites are indicated in blue (K238R, **b**) and green (K412R, **c**).

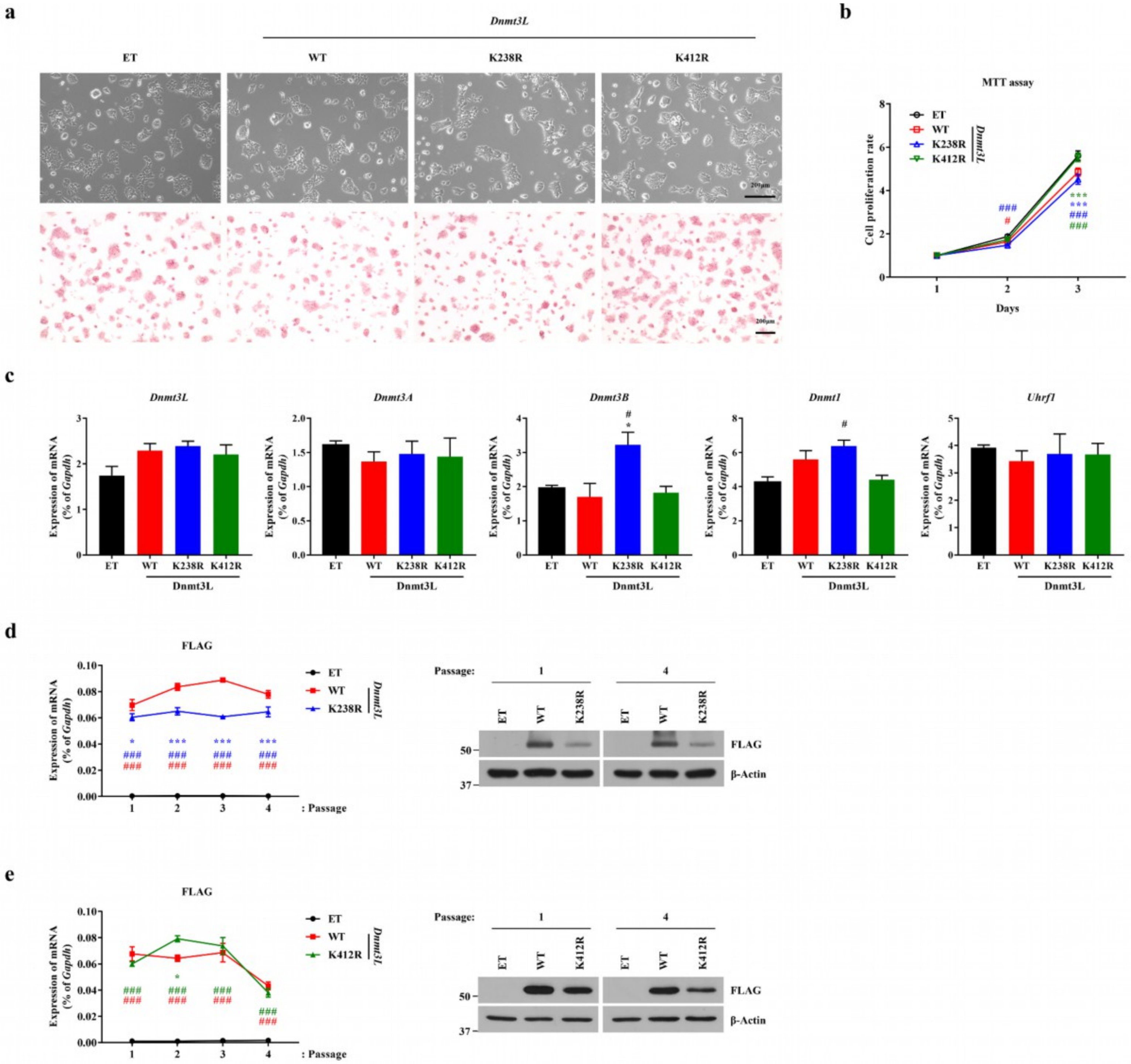


**Supplementary Fig. 5. Post-transcriptional regulation of Dnmt3L protein stability in mESCs**

**(a)** Representative images showing colony morphology (upper) and AP staining (lower) of mESCs stably expressing FLAG-tagged *Dnmt3L* WT, K238R, or K412R. Images were

acquired at ´100 magnification. Scale bar, 200 µm. **(b)** Proliferation of the indicated cell lines measured by the MTT assay over 3 days (*n* = 5). **(c)** RT-qPCR analysis of transcripts encoding total *Dnmt3L* (endogenous) and other DNA methylation regulators in the indicated stable mESC lines (*n* = 3). Expression levels were normalized to *Gapdh*. **(d and e)** Time-course analysis of transcript and protein levels of FLAG-tagged Dnmt3L K283R **(d)** or K412R **(e)** across four passages of mESCs cultured in FBS-LIF condition. mRNA expression was quantified by RT-qPCR and normalized to *Gapdh* (*n* = 3), while protein levels were assessed by immunoblotting, with β-actin used as a loading control. All data are presented as mean ± SEM. Statistical significance was determined using one-way **(c)** or two-way **(b, d, and e)** ANOVA followed by Bonferroni post hoc tests. *p < 0.05, ***p < 0.001 vs. WT Dnmt3L; #p < 0.05, ###p < 0.001 vs. empty (ET) vector control.

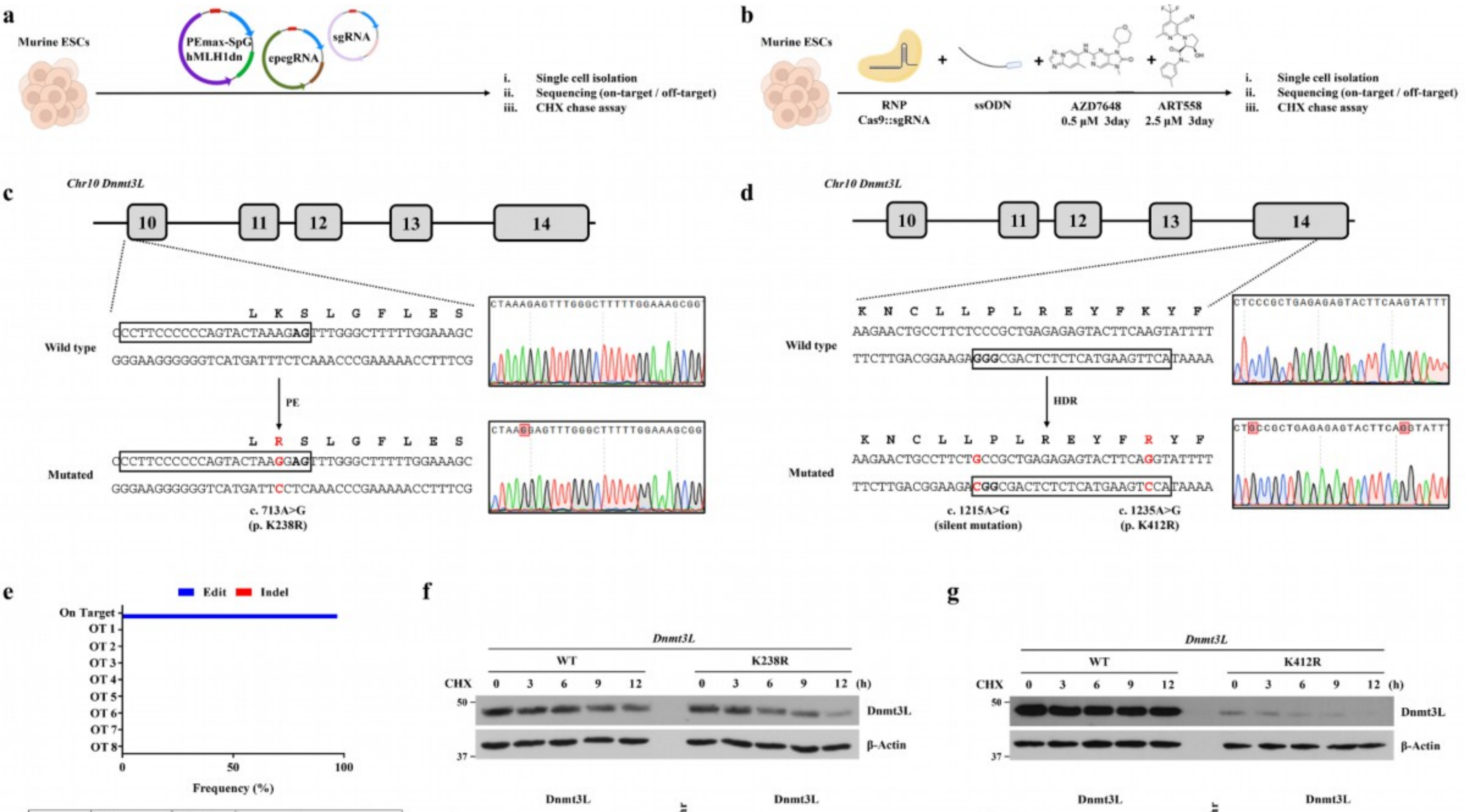


| | Chromosome | Position | Sequence |
|---|---|---|---|
| On Target | Chr10 | 77899131 | ACTTGAAGTACTCTCTCAGC |
| OT1 | Chr1 | 103647915 | ACTTGAAGTACTTtaCTCAGg |
| OT2 | Chr1 | 189962994 | ACTTGAAtcACTCTCTaAGC |
| OT3 | Chr6 | 112472084 | ACTTGAAtTAtTCTCcCAGC |
| OT4 | Chr7 | 118177687 | ACTTGAgGTACTCTCcCAGa |
| OT5 | Chr8 | 25604386 | tCTTGAAGTACTCgCTCAGg |
| OT6 | Chr14 | 26054716 | ACTTGAAGaACTCTCTCAtt |
| OT7 | Chr16 | 16518596 | ACTTaAAGTACTCaCaCAGC |
| OT8 | Chr17 | 26972171 | ACTTGAtGTtCTgTCTCAGC |

**Supplementary Fig. 6. Endogenous K238R and K412R mutations destabilize the Dnmt3L protein.**

**(a and b)** Schematic diagrams depicting the generating of endogenous *Dnmt3L* K238R **(a)** and K412R **(b)** point mutations in R1 mESCs *via* prime editing **(a)** and ssODN-mediated homology-directed repair (HDR) **(b)**, respectively. Following electroporation for ssODN-mediated HDR, cells were cultured for 72 hours with 0.5 mM AZD-7648 (a selective DNA-PK inhibitor) and 2.5 mM ART558 (a selective DNA polymerase theta inhibitor). These inhibitors were used to enhance HDR efficiency and suppress large deletions and rearrangements, respectively. **(c and d)** Representative Sanger sequencing chromatograms

confirming the successful introduction of the K238R **(c)** and K412R **(d)** substitutions at the endogenous *Dnmt3L* locus. **(e)** Off-target (OT) analysis for the K412 HDR-edited cell lines by Sanger sequencing of the top eight candidate loci (allowing up to three mismatches) predicted by *in silico* analysis revealed no detectable off-target mutations. **(f and g)** Cycloheximide (CHX) chase assays demonstrating reduced half-lives of the endogenous K238R **(f)** and K412R **(g)** mutants, compared with WT Dnmt3L protein (*n* =3). All quantitative data are shown as mean ± SEM. Statistical analysis was performed using two-way ANOVA with the Bonferroni post hoc test. $^{**}p < 0.01$, $^{***}p < 0.001$ compared with the WT Dnmt3L group. ssODN, single-stranded oligodeoxynucleotide (ssODN); HDR, homology-directed repair.

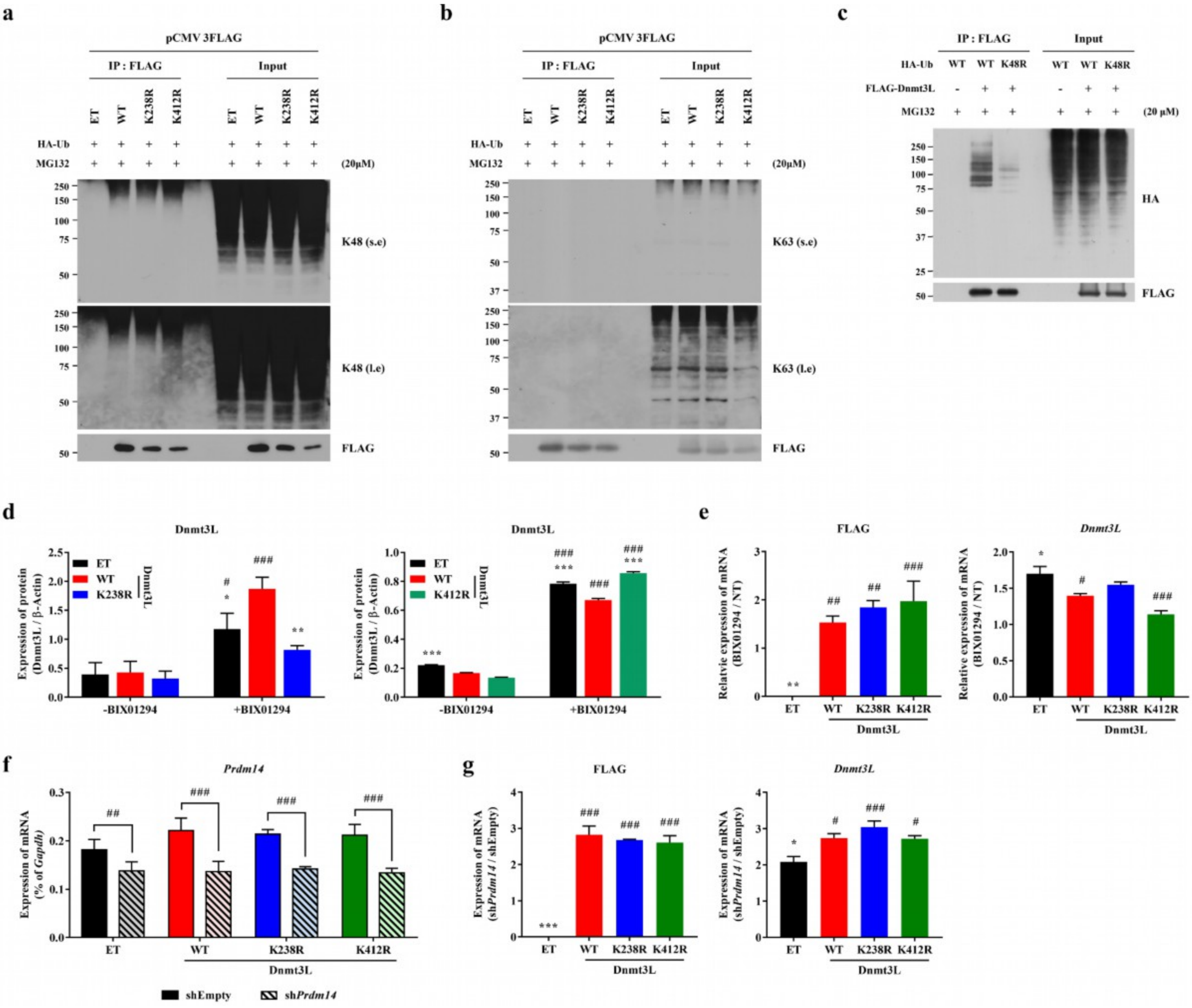


**Supplementary Fig. 7. Role of G9a and Prdm14 in regulation of Dnmt3L protein stability in mESCs**

**(a and b)** Western blot analysis of K48-linked **(a)** and K63-linked **(b)** polyubiquitination of Dnmt3L. HA-tagged ubiquitin (HA-Ub) and FLAG-tagged *Dnmt3L* wild-type (WT) or mutants (K238R and K412R) were co-transfected into cells, followed by FLAG immunoprecipitation (IP). Immunoprecipitated samples were probed with antibodies specific

to K48 or K63 polyubiquitin chains. **(c)** FLAG-Dnmt3L ubiquitination levels assessed by FLAG-IP after co-transfection with FLAG-*Dnmt3L* and either HA-Ub-WT or HA-Ub-K48R. Input lanes represent 20% of the total cell lysates used for IP assays. **(d)** Quantification of endogenous Dnmt3L protein levels by densitometric analysis following treatment for 24 h with 3 μM BIX01294 (BIX), a G9a inhibitor, in mESCs expressing Dnmt3L WT, K238R, or K412R ($n$ = 3). **(e)** RT-qPCR quantification of total (FLAG-tagged exogenous and endogenous) *Dnmt3L* transcripts following BIX01294 treatment ($n$ = 3). Data are presented as fold change relative to the untreated (NT) group. **(f)** Efficiency of *Prdm14* knockdown in mESCs stably expressing WT *Dnmt3L* or mutants assessed by RT-qPCR ($n$ = 4). **(g)** Expression levels of FLAG-tagged and endogenous *Dnmt3L* transcripts in cells following *Prdm14* silencing ($n$ = 4). All data are shown as mean ± SEM. Statistical analysis was performed using a one-way **(e and g)** or two-way **(d and f)** ANOVA with the Bonferroni post hoc test. *p < 0.05, **p < 0.01, ***p < 0.001 compared with the WT Dnmt3L group. #p < 0.05, ##p < 0.01, ###p < 0.001 compared with the empty (ET) **(e and g)**, BIX01294 non-treated **(d)**, or *Prdm14* knock-down **(f)** groups.

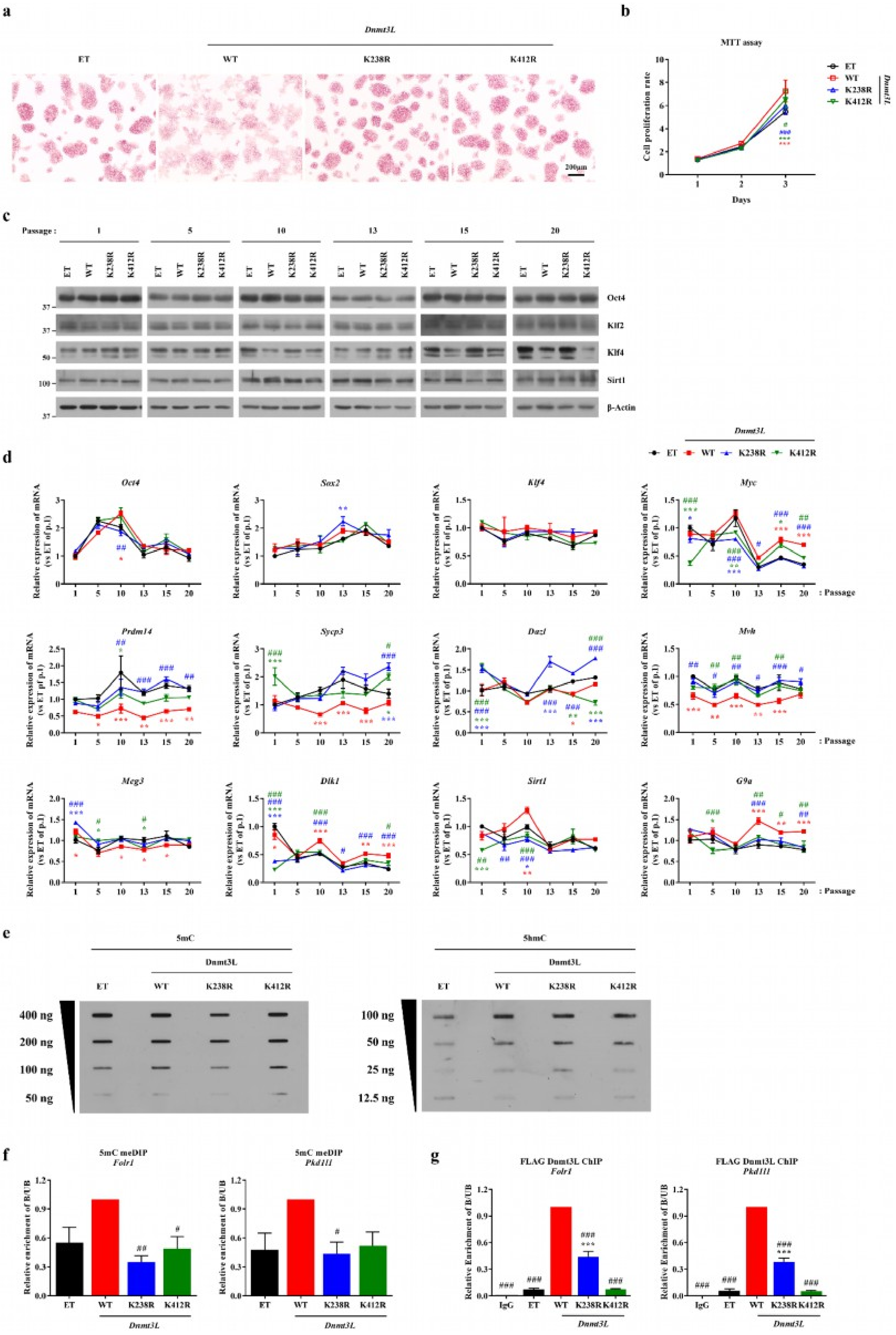
a
Dnmt3L
ET
WT
K238R
K412R
200μm
b
MTT assay
Cell proliferation rate
Days
ET
WT
K238R
K412R
Dnmt3L
c
Passage :
1
5
10
13
15
20
Oct4
Klf2
Klf4
Sirt1
β-Actin
d
Dnmt3L
ET
WT
K238R
K412R
Oct4
Sox2
Klf4
Myc
Prdm14
Sycp3
Dazl
Mvh
Meg3
Dlk1
Sirt1
G9a
Relative expression of mRNA (vs ET of p.1)
: Passage
e
5mC
5hmC
Dnmt3L
ET
WT
K238R
K412R
400 ng
200 ng
100 ng
50 ng
25 ng
12.5 ng
f
5mC meDIP
Folr1
Pkd1l1
Relative enrichment of B/UB
ET
WT
K238R
K412R
Dnmt3L
g
FLAG Dnmt3L ChIP
Folr1
Pkd1l1
Relative Enrichment of B/UB
IgG
ET
WT
K238R
K412R
Dnmt3L

**Supplementary Fig. 8. Dnmt3L acetylation controls pluripotency and expression of lineage differentiation genes through locus-specific DNA methylation**

**(a)** Representative images of AP staining in mESCs stably expressing non-tagged *Dnmt3L* WT, K238R, or K412R after long-term culture. Images were acquired at ×100 magnification. Scale bar, 200 μm. **(b)** Proliferation of the indicated stable mESC lines measured by the MTT assay over 3 days (*n* = 5). **(c)** Western blot analysis of proteins related to pluripotency and DNA methylation regulation in mESCs stably expressing WT *Dnmt3L* or acetylation-deficient mutants following prolonged culture. **(d)** RT-qPCR analysis of transcripts related to naïve or cardinal pluripotency, germline differentiation, and epigenetic regulation at the indicated passages. Expression values are shown as fold changes relative to p.1 of the empty vector control (*n* = 3). **(e)** Dot blot analysis of global 5mC (left panel) and 5hmC (right panel) levels in the indicated stable mESC lines. **(f)** MeDIP-qPCR analysis of 5mC enrichment at the regulatory regions of *Folr1* (left panel) and *Pkd1l1* (right panel) loci (*n* = 4). **(g)** ChIP-qPCR analysis of FLAG-tagged Dnmt3L WT, K238R, and K412R at the regulatory regions of *Folr1* (left panel) and *Pkd1l1* (right panel) (*n* = 4), demonstrating the reduced binding of acetylation-deficient mutants. All quantitative data are shown as mean ± SEM. Statistical significance was determined using a two-way **(b and d)** or one-way **(f and g)** ANOVA with the Bonferroni post hoc test. *p < 0.05, **p < 0.01, ***p < 0.001 compared with empty (ET) control groups. #p < 0.05, ##p < 0.01, ###p < 0.001 compared with WT Dnmt3L.

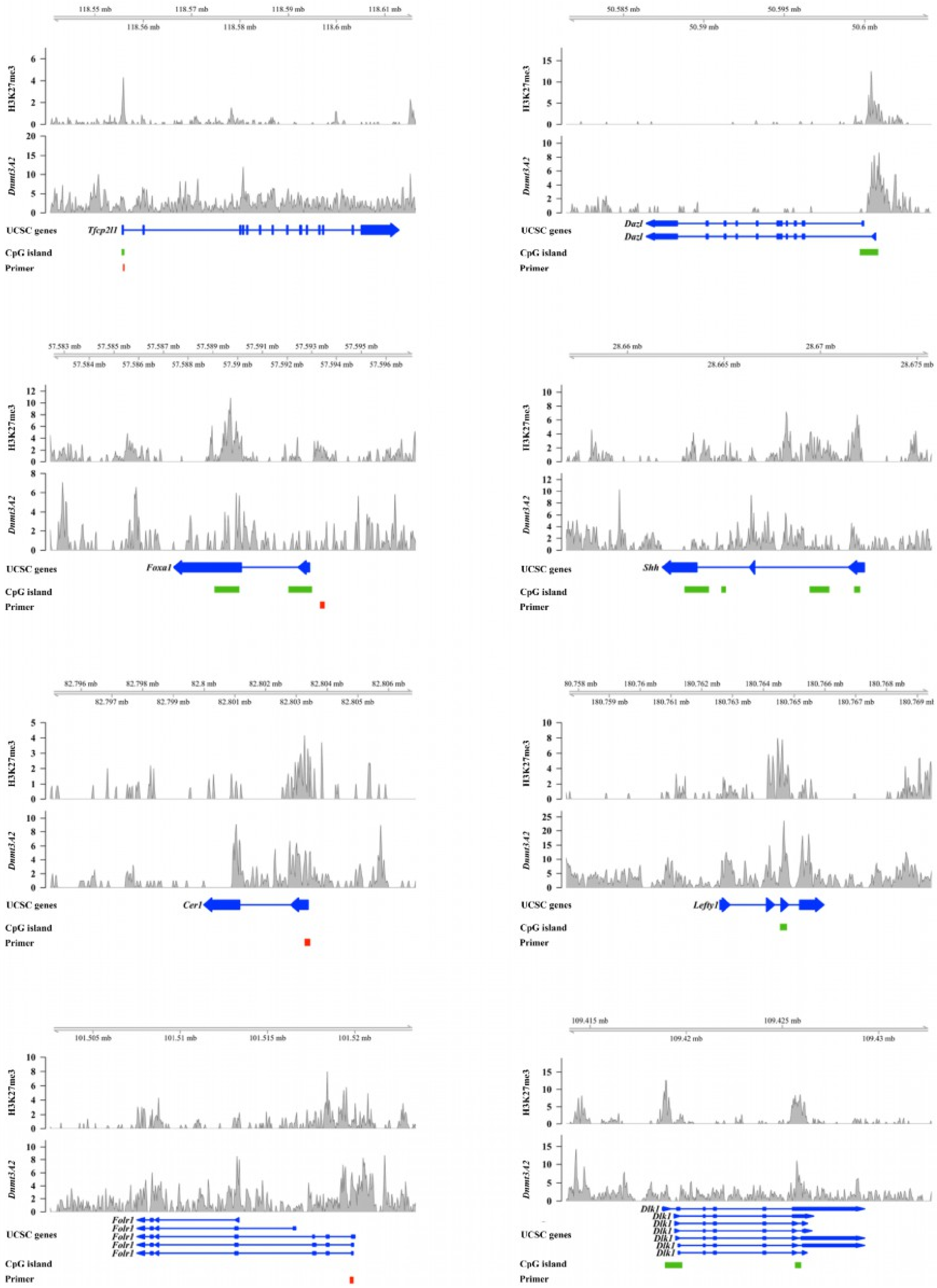
118.55 mb
118.56 mb
118.57 mb
118.58 mb
118.59 mb
118.6 mb
118.61 mb
H3K27me3
Dnmt3A2
UCSC genes
Tfcp2l1
CpG island
Primer
50.585 mb
50.59 mb
50.595 mb
50.6 mb
H3K27me3
Dnmt3A2
UCSC genes
Dazl
Dazl
CpG island
Primer
57.583 mb
57.584 mb
57.585 mb
57.586 mb
57.587 mb
57.588 mb
57.589 mb
57.59 mb
57.591 mb
57.592 mb
57.593 mb
57.594 mb
57.595 mb
57.596 mb
H3K27me3
Dnmt3A2
UCSC genes
Foxa1
CpG island
Primer
28.66 mb
28.665 mb
28.67 mb
28.675 mb
H3K27me3
Dnmt3A2
UCSC genes
Shh
CpG island
Primer
82.796 mb
82.797 mb
82.798 mb
82.799 mb
82.8 mb
82.801 mb
82.802 mb
82.803 mb
82.804 mb
82.805 mb
82.806 mb
H3K27me3
Dnmt3A2
UCSC genes
Cer1
CpG island
Primer
180.758 mb
180.759 mb
180.76 mb
180.761 mb
180.762 mb
180.763 mb
180.764 mb
180.765 mb
180.766 mb
180.767 mb
180.768 mb
180.769 mb
H3K27me3
Dnmt3A2
UCSC genes
Lefty1
CpG island
Primer
101.505 mb
101.51 mb
101.515 mb
101.52 mb
H3K27me3
Dnmt3A2
Folr1
Folr1
Folr1
Folr1
Folr1
UCSC genes
CpG island
Primer
109.415 mb
109.42 mb
109.425 mb
109.43 mb
H3K27me3
Dnmt3A2
Dlk1
Dlk1
Dlk1
Dlk1
Dlk1
Dlk1
Dlk1
UCSC genes
CpG island
Primer

**Supplementary Fig. 9. Epigenetic profiles of Dnmt3L acetylation-sensitive target genes in mESCs**

Schematic representation of representative gene loci (*Tfcp2l1*, *Dazl*, *Foxa1*, *Shh*, *Cer1*, *Lefty1, Folr1*, and *Dlk1*) affected by Dnmt3L K238Ac or K412Ac. UCSC Genome Browser tracks are shown for H3K27me3 histone modification and Dnmt3A2 binding based on previously published ChIP-seq datasets [1,2]. Genomic features include CpG islands (green bars) and locations of primers used for MeDIP- and ChIP-qPCR assays in this study (red bars). These loci represent epigenetically regulated targets exhibiting differential transcriptional responses depending on the Dnmt3L acetylation status.

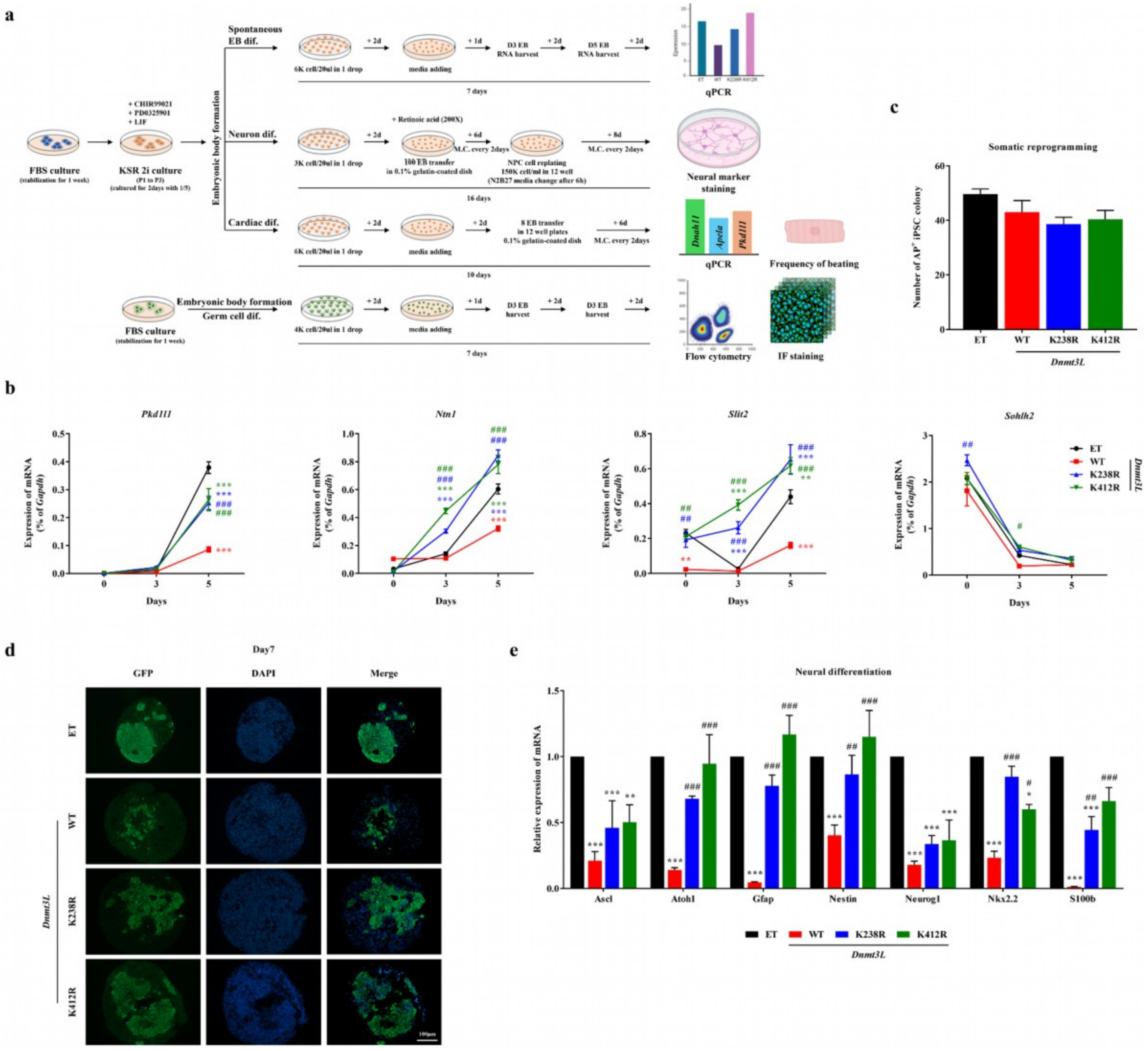


**Supplementary Fig. 10. Characterization of *in vitro* differentiation phenotypes regulated by Dnmt3L acetylation in mESCs**

**(a)** Schematic summary of the detailed experimental conditions for assessment of germline, neural, and cardiac lineage markers in EB-based differentiation assays. **(b)** RT-qPCR analysis of additional cardiac (*Pkd1l1*), neural (*Ntn1* and *Slit2*) and germline (*Sohlh2*) markers during

spontaneous differentiation of mESCs stably expressing *Dnmt3L* WT, K238R, or K412R. Expression values were normalized to *Gapdh* ($n$ = 3). **(c)** Quantification of $AP^+$ iPSC colonies at day 14 post-reprogramming of DOX-inducible iOSKM MEFs. Reprogramming efficiency was unaffected by *Dnmt3L* mutations ($n$ = 5). **(d)** IF analysis of $GFP^+$ PGCs in EB sections derived from gcOct4 mESCs at day 7 post-differentiation. Numbers of $GFP^+$ cells were reduced in WT *Dnmt3L* EBs compared with the control and mutant groups. Images were acquired at ×200 magnification. Scale bar, 100 μm. **(e)** RT-qPCR analysis of the indicated neural differentiation markers following directed neuronal differentiation of mESCs ($n$ = 3). All quantitative data are shown as mean ± SEM. Statistical comparisons were performed using a two-way ANOVA with the Bonferroni post hoc test. *$p < 0.05$, **$p < 0.01$, ***$p < 0.001$ compared with empty (ET) control groups. #$p < 0.05$, ##$p < 0.01$, ###$p < 0.001$ compared with WT Dnmt3L.

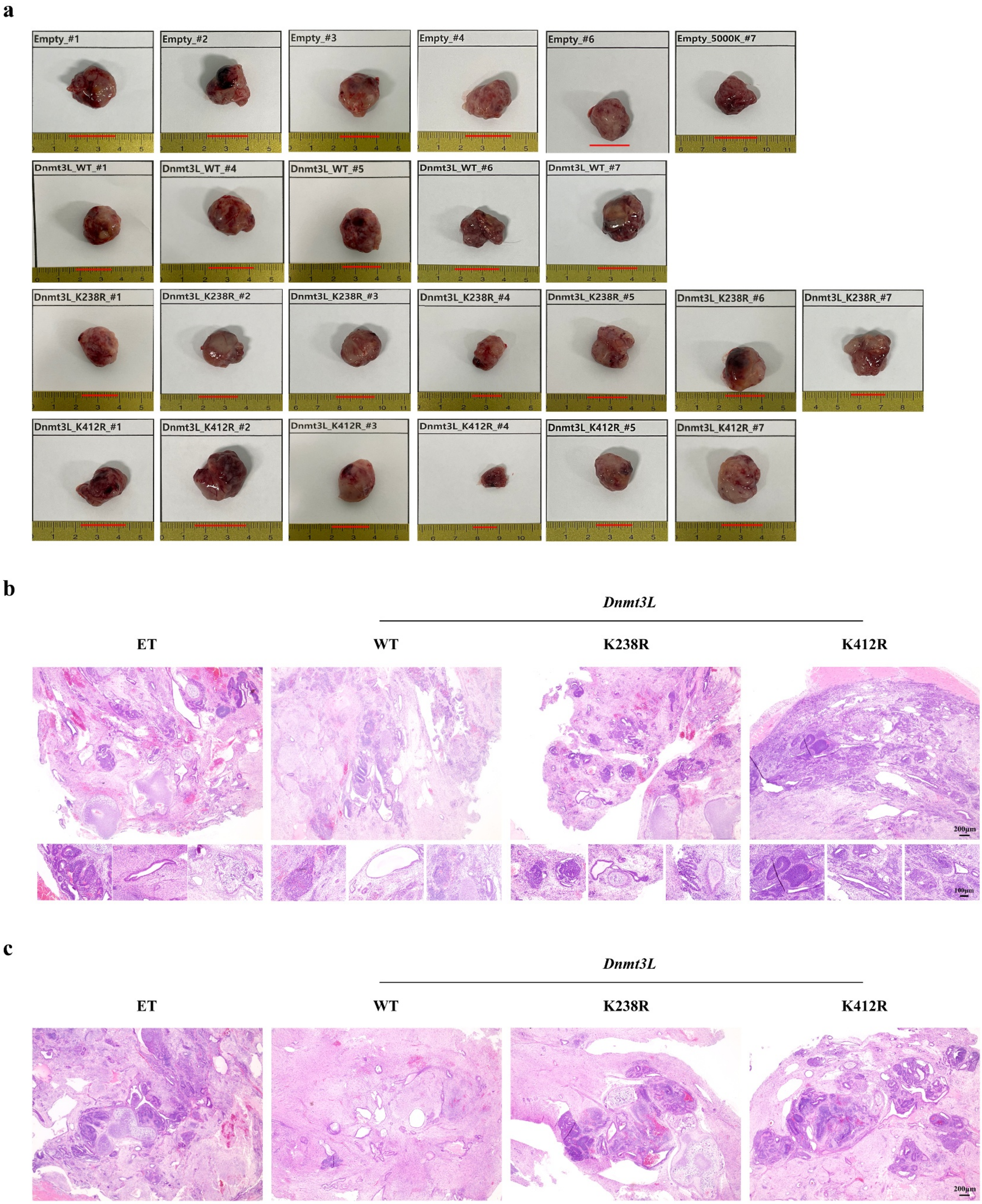
a
Empty_#1
Empty_#2
Empty_#3
Empty_#4
Empty_#6
Empty_5000K_#7
Dnmt3L_WT_#1
Dnmt3L_WT_#4
Dnmt3L_WT_#5
Dnmt3L_WT_#6
Dnmt3L_WT_#7
Dnmt3L_K238R_#1
Dnmt3L_K238R_#2
Dnmt3L_K238R_#3
Dnmt3L_K238R_#4
Dnmt3L_K238R_#5
Dnmt3L_K238R_#6
Dnmt3L_K238R_#7
Dnmt3L_K412R_#1
Dnmt3L_K412R_#2
Dnmt3L_K412R_#3
Dnmt3L_K412R_#4
Dnmt3L_K412R_#5
Dnmt3L_K412R_#7
b
Dnmt3L
ET
WT
K238R
K412R
200μm
100μm
c
Dnmt3L
ET
WT
K238R
K412R
200μm

**Supplementary Fig. 11. *In vivo* differentiation outcomes modulated by Dnmt3L acetylation**

**(a)** Representative gross images of teratomas collected at day 46 following subcutaneous injection of mESCs stably expressing *Dnmt3L* WT, K238R, or K412R. Tumor growth was monitored longitudinally over time. **(b)** Representative histological sections of teratomas harvested at day 21 post-injection and stained with hematoxylin and eosin to evaluate contributions from all three germ layers. Distinct ectodermal, mesodermal, and endodermal derivatives are highlighted in the corresponding inset panels (Top panel: ×40 magnification; scale bar, 200 µm, inset panel: ×200 magnification; scale bar, 100 µm). **(c)** WT *Dnmt3L* teratomas exhibited a marked reduction in mesodermal and endodermal tissue organization compared with the mutant and control groups (×40 magnification; scale bar, 200 µm).

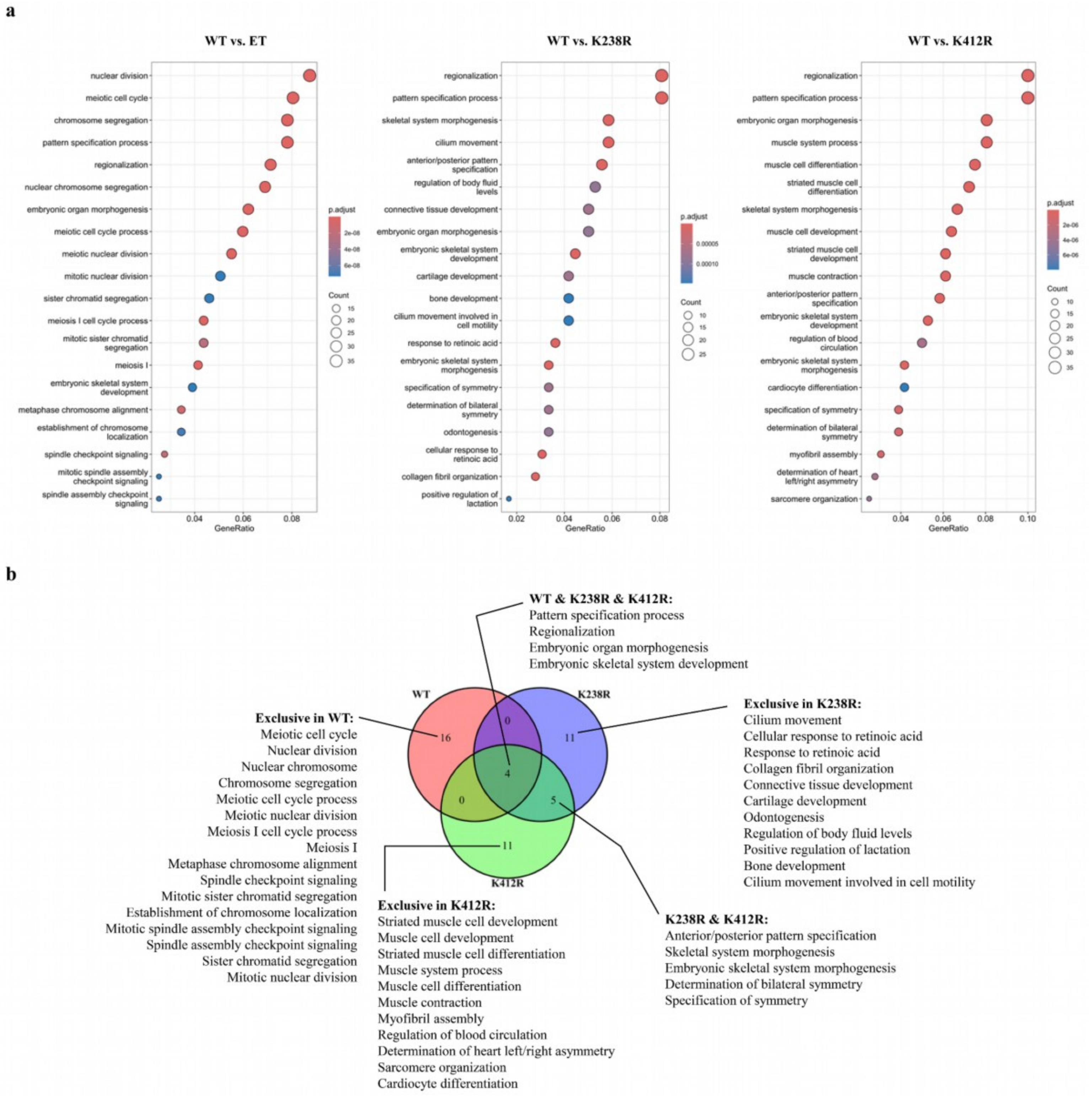


**Supplementary Fig. 12. Global transcriptomic effects of Dnmt3L acetylation deficiency in *in vivo* teratoma**

**(a)** Gene Ontology (GO) enrichment analysis of differentially expressed genes (DEGs; ³ 2-fold

change, $p < 0.05$) comparing teratomas derived from mESCs overexpressing wild-type (WT) *Dnmt3L* versus the empty vector (control) or acetylation-deficient mutants (K238R or K412R). The top 20 GO terms for each comparison are shown. Terms related to with meiosis, a hallmark of germline specification, were enriched in the WT versus control comparison. **(b)** Venn diagram displaying the overlap of the top 20 enriched GO terms across each group comparison. Notably, minimal overlap was observed between the WT versus control and WT versus mutant comparisons, indicating distinct GO enrichment patterns across each group comparison.

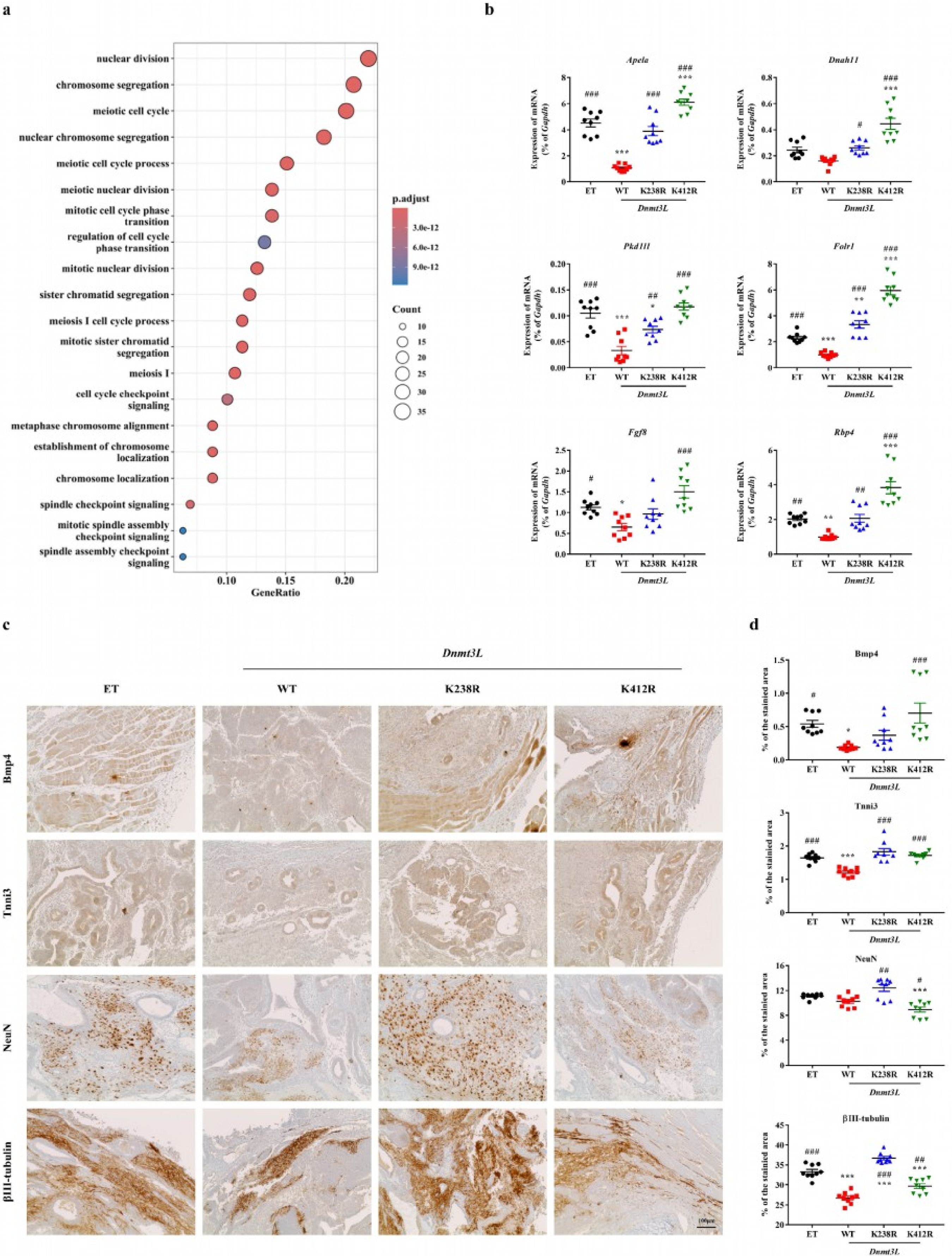
a
nuclear division
chromosome segregation
meiotic cell cycle
nuclear chromosome segregation
meiotic cell cycle process
meiotic nuclear division
mitotic cell cycle phase transition
regulation of cell cycle phase transition
mitotic nuclear division
sister chromatid segregation
meiosis I cell cycle process
mitotic sister chromatid segregation
meiosis I
cell cycle checkpoint signaling
metaphase chromosome alignment
establishment of chromosome localization
chromosome localization
spindle checkpoint signaling
mitotic spindle assembly checkpoint signaling
spindle assembly checkpoint signaling
0.10
0.15
0.20
GeneRatio
p.adjust
3.0e-12
6.0e-12
9.0e-12
Count
10
15
20
25
30
35
b
Apela
Dnah11
Pkd1l1
Folr1
Fgf8
Rbp4
Expression of mRNA (% of Gapdh)
ET
WT
K238R
K412R
Dnmt3L
c
Dnmt3L
ET
WT
K238R
K412R
Bmp4
Tnni3
NeuN
βIII-tubulin
d
Bmp4
Tnni3
NeuN
βIII-tubulin
% of the stainied area
ET
WT
K238R
K412R
Dnmt3L

**Supplementary Fig. 13. Transcriptomic and histological evidence of impaired lineage differentiation in WT *Dnmt3L* teratomas**

**(a)** GO enrichment analysis of the downregulated gene cluster (cluster 3 in **Fig. 7c**) in WT *Dnmt3L* teratomas, which was restored in the mutant groups. Functional annotation revealed strong enrichment of pathways involved in germline development. **(b)** RT-qPCR validation of representative lineage markers across teratoma tissues. Cardiac regulators (*Apela, Dnah11* and *Pkd1l1*), neural markers (*Folr1*, *Fgf8*, and *Rbp4*) were significantly repressed in WT *Dnmt3L* samples and recovered in the mutant groups. Expression levels were normalized to *Gapdh* (*n* = 9). **(c)** Immunohistochemistry staining of cardiac ($Tnni3^+$ and $Bmp4^+$) and neural ($NeuN^+$ and βIII -$tubulin^+$) and markers in teratoma sections collected at day 21 post-injection. Images were captured at ×200 magnification. Scale bar, 100 µm. **(d)** Quantification of lineage marker expressing cells from immunostained sections (*n* = 9). Results confirmed that the neural and cardiac differentiation capacities were significantly impaired in teratomas expressing WT *Dnmt3L*. All quantitative data are presented as mean ± SEM. Statistical comparisons were performed using a one-way ANOVA with the Bonferroni post hoc test. *p < 0.05, **p < 0.01, ***p < 0.001 vs. empty (ET) control group; ##p < 0.01, ###p < 0.001 vs. WT *Dnmt3L*.

## SUPPLEMENTARY FIGURE LEGENDS

**Supplementary Movie 1. Defects of *in vitro* cardiac differentiation induced by Dnmt3L acetylation mutations**

Phase-contrast videoclips showing the morphology of beating EBs during cardiac differentiation. EBs expressing WT *Dnmt3L* exhibited flattened structures lacking organized cavity formation, in contrast to the round and contractile EBs in the control and mutant groups.

## SUPPLEMENTARY MATERIALS AND METHODS

***Structure preparation for molecular dynamics (MD) simulation***

The full-length three-dimensional structure of mouse DNA methyltransferase 3-like (Dnmt3L) was obtained from the AlphaFold Protein Structure Database (UniProt ID: Q9CWR8). This high-confidence model provided a complete starting structure for simulations. Single-point m utation of lysine residues 238 and 412 to arginine (K238R and K412R) mutants were generated *in silico* using AlphaFold2 [3]. For protein–protein complex simulations, the crystal structure of the DNA methyltransferase 3A (Dnmt3A)–Dnmt3L C-terminal domain complex (PDB ID: 2QRV) was used as a template. Missing residues in the PDB complex (primarily in loops or termini) were filled using the AlphaFold full-length Dnmt3L structure. CHARMM-GUI, a web-based interface for biomolecular simulations, was used for acetylated variants [4]. The Solution Builder module was employed to set up the systems for acetylation studies, which included various acetylation states such as fully acetylated (WT Ac) and non-acetylated (WT non-Ac) Dnmt3L, as well as single and double-acetylated variants. For simulations, the acetylation patch was applied to the lysine residues, and additional patches were auto-applied for cysteine and aspartate residues. The structures were placed in a periodic simulation box, with CHARMM-GUI automatically generating the boxes for each system. For the single-protein systems, CHARMM-GUI generated a cubic simulation box with side lengths of approximately 9.8 × 9.8 × 9.8 nm along the X, Y, and Z axes. All box angles were set to 90° ($\alpha = \beta = \gamma$), corresponding to a perfect cubic geometry, and the protein was positioned with a minimum distance of 1.0 nm from the box boundaries. For the larger Dnmt3A–Dnmt3L complex, an orthorhombic box measuring approximately 15.5 × 15.5 × 30 nm was chosen. The elongated z-dimension provided sufficient space along the pulling axis for steered MD (SMD). The systems were solvated using a Monte Carlo method to place $Na^+$ and $Cl^-$ ions at

a concentration of ~0.15 M. For standard MD simulations, the setups prepared in CHARMM-GUI used the AMBER-FF19SB force field with the OPC water model, at 310 K and neutral pH (pH 7.0) [5]. CHARMM-GUI prepared the systems in a GROMACS-compatible format, and these files were subsequently used for energy minimization, equilibration, and production phases [6]. For SMD simulations, the complex structures generated by CHARMM-GUI were exported and the systems were rebuilt in GROMACS using the AMBER99SB-ILDN force field with the TIP3P water model [7].

***Standard MD simulation***

All MD simulations were performed with GROMACS 2023.3 [8]. After solvation and ion addition, each system was energy-minimized using the steepest descent algorithm (up to 5,000 steps) until the maximum force fell below 1000 kJ/mol·nm. Equilibration was carried out in two phases using a single CHARMM-GUI-generated protocol, in which positional restraints were applied to backbone (440 kJ/mol·nm$^2$) and side-chain atoms (40 kJ/mol·nm$^2$) to maintain the folded protein structure while the solvent equilibrated. The system was equilibrated at 310 K and 1 bar for 0.5 ns with a time step of 0.001 ps, using the velocity-rescaling (V-rescale) thermostat for temperature control and pressure coupling to adjust density. Periodic boundary conditions were applied in all directions, and long-range electrostatics were computed with the Particle-Mesh Ewald method [9]. All covalent bonds involving hydrogen were constrained using the LINCS algorithm [10]. Temperature and pressure coupling were also kept applied during the production runs (NPT ensemble at 310 K and 1 bar) to mimic physiological conditions. Production MD simulations were then executed for 500 ns for each Dnmt3L variant [WT Ac, acetylation at K238 (K238Ac) or K412 (K412Ac), WT non-Ac, K238R, and K412R]. A single run per variant was performed, each starting from the same equilibrated structure but with different initial velocity seeds at 310 K. Trajectory coordinates were saved

every 100 ps for analysis. Structural stability and flexibility were evaluated using standard metrics. The backbone root mean square deviation (RMSD) of each protein was calculated as a function of time to monitor global conformational drift. Likewise, per-residue root mean square fluctuation (RMSF) of Cα atoms was computed over the equilibrated portion of each trajectory to assess local flexibility. To compare overall conformational variability between variants, the RMSD amplitude across the simulation was calculated, defined as the range of RMSD values (max-min) observed within each nanosecond window. The average RMSD amplitude was quantified by averaging the RMSD amplitudes (range of values) across the entire 500-ns simulation. A lower average amplitude indicates more consistent stable behavior across the simulation for a given variant.

***SMD simulation***

To probe the effect of the mutations on the interaction of Dnmt3L with Dnmt3A, SMD pulling simulations were performed using the Dnmt3A–Dnmt3L complex. The equilibrated complex structure (WT Dnmt3L or its mutants) served as the starting point for each SMD run. In these simulations, the Dnmt3A portion was defined as the receptor and kept fixed in space to mimic a rigid binding partner. All heavy atoms of Dnmt3A were restrained with a very high force constant (1000 kJ/mol·nm$^2$), which effectively immobilized Dnmt3A during the pulling. Dnmt3L (WT or mutants, designated as the ligand) was subjected to an external pulling force in order to induce unbinding from Dnmt3A. The complex was aligned such that the pulling vector was approximately along the z-axis of the simulation box (coinciding with the longest box dimension). A harmonic spring potential was attached between the center-of-mass of Dnmt3L and a moving reference point that was withdrawn from the complex at a constant velocity. Specifically, a constant-velocity pulling scheme was used: the reference point was moved away from the receptor at a speed of 0.005 nm·ps$^{-1}$ (5 nm·ns$^{-1}$) along +z, with a spring

constant of 1000 kJ/mol·nm$^2$ governing the tether. This setup ensured that as Dnmt3L was pulled, a restoring force was generated that increased until key contacts at the interface ruptured and Dnmt3L dissociated. All SMD simulations employed the same force field (AMBER99SB) and solvent model as the standard MD at 310 K. Each SMD simulation was run for 500 ps, which was sufficient to disrupt the Dnmt3A–Dnmt3L interface and separate the proteins by several nanometers. A single pulling simulation was performed for each Dnmt3L variant (WT Ac, K238Ac, K412Ac, WT non-Ac, K238R, and K412R) to obtain results for each variant. Throughout the pulling simulations, force-extension data were recorded at 1-ps intervals.

***Establishment of endogenous Dnmt3L K238R and K412 mutant cell lines***

Two independent mouse embryonic stem cell (mESC) lines harboring endogenous point mutations at the *Dnmt3L* K238 and K412 residues was generated. The *Dnmt3L* K238R point mutant cell line was established using a prime editing strategy [11,12]. Briefly, R1 mESCs were co-transfected with a prime editing component set consisting of PE5max-SpG-P2A-hMLH1dn (Addgene, # 211550), a nicking guide RNA (ngRNA) expression vector (pRG2, Addgene #104174), and a engineered prime editing guide RNA (epegRNA) expressing plasmid (Addgene, # 174038) using Lipofectamine 2000.

For generation of the *Dnmt3L* K412R mutant, homology-directed repair was employed via a Cas9 ribonucleoprotein (RNP)-based genome editing [13,14]. *In vitro* transcribed single guide RNA (sgRNA) targeting the K412 locus was synthesized using T7 RNA polymerase (New England Biolabs, Ipswich, MA, USA) and purified with the RNeasy Mini Kit (Qiagen, Hilden, Germany). RNP complexes were assembled by incubating recombinant *Streptococcus pyogenes* Cas9 protein (Enzynomics, Deajeon, South Korea) with the purified sgRNA at a 1:3 molar ratio for 30 minutes at room temperature. Subsequently, 200 pmol of a single-stranded oligodeoxynucleotide (ssODN) donor template was added to the pre-assembled

RNP complex and then electroporated into 1 x $10^5$ R1 ESCs using the Neon NxT Electroporation System (Thermo Fisher Scientific) under the following conditions: 1200 V, 20 ms, and 2 pulses. Following electroporation, cells were incubated 0.5 mM AZD7648 (MedChemExpress, Monmouth Junction, NJ, USA) and 2.5 mM ART558 (MedChemExpress) for 72 hours [15,16]. All spacer sequences for the sgRNA, epegRNA, ngRNA used to generate the K238R and K412R lines were designed with the Cas-Designer [17] and the sequences are listed in the key resources table. The *in silico* off-target analysis for the K412R cell line was performed with Cas-OFFinder [18] allowing for up to 3 mismatches.

***Reverse transcription (RT)-polymerase chain reaction (PCR) for gene expression analysis***

The mRNA levels of target genes were quantitatively assessed as described previously [19,20]. Total RNA (800 ng) was reverse-transcribed using Taqman Reverse Transcription Reagents (Applied Biosystems, Foster City, CA, USA), and the threshold cycle (Ct) was subsequently fdetermined using RT-quantitative PCR (qPCR). The relative expression levels of the target genes were determined using the $2^{-DDCt}$ method, and *Gapdh* was used as the endogenous control gene. All primers used in the qPCR assay are listed in the key resources table.

***Western blot analysis***

Cell extracts (30 mg) were prepared in immunoprecipitation (IP) lysis buffer and separated on 10% SDS-PAGE gels. The expression levels of the indicated proteins were assessed by probing with specific antibodies, which are listed in the key resources table. To quantify the densities of the indicated protein bands, quantitative digital image analysis was performed using ImageJ software (version 1.54g; National Institute of Health, Bethesda, MD, USA). Relative protein expression was calculated by normalization to b-Actin.

***Immunostaining assays***

mESCs or cells differentiated from them were fixed with 4% paraformaldehyde (Biosesang, Seongnam-si, Gyeonggi-do, Korea) for 30 min, permeabilized, and then stained using specific antibodies, which are listed in the key resources table. An Alexa Fluor 488- or Alexa Fluor 546-conjugated anti-mouse or anti-rabbit secondary antibody was used (Molecular Probes/Thermo Fisher Scientific, Waltham, MA, USA). Nuclei were counterstained with 4',6-diamino-2-phenylindole (DAPI). The stained samples were imaged using an inverted fluorescence microscope (EVOS® FL Color Imaging System, Thermo Fisher Scientific) or a confocal microscope (LSM 710; Carl Zeiss, Oberkochen, Germany).

***Proximity ligation assay***

To detect *in situ* acetylation of Dnmt3L, proximity ligation assay (PLA) was performed using the NaveniFlex™ Cell Kit (Navinci Diagnostics AB, Uppsala, Sweden). R1 mESCs were fixed with 4% paraformaldehyde for 1h at room temperature and permeabilized with 0.1% Triton X-100 for 5 min. After blocking with Naveni Block for 60 min at 37 °C, cells were incubated overnight at 4 °C with primary antibodies against acetyl-lysine and Dnmt3L. Following washes, the Navenibody M1 and R2 probes (1:40 dilution) were applied for 60 min at 37 °C, followed by a 30 min ligation reaction. Fluorescent signal amplification was performed using NaveniFlex Cell Buffer 2 Red for 90 min at 37 °C in the dark. Nuclei were counterstained with DAPI, and samples were mounted with antifade medium. PLA signals corresponding to acetylated Dnmt3L were visualized using a Zeiss LSM710 confocal microscope. Negative controls omitting primary antibodies were included to assess nonspecific background fluorescence.

***DNA methylation analysis***

The DNA methylation statuses of individual loci were investigated using bisulfite sequencing or quantified using qPCR of 5-methylcytosine (5mC)-enriched DNA obtained through methyl ated DNA IP (meDIP), as previously described [21,22]. For bisulfite sequencing, 1μg of genomi c DNA was bisulfite-converted using the EZ DNA Methylation-Gold™ Kit (Zymo Research, Irvine, CA, USA) according to the manufacturer's protocol. For meDIP analysis, 1μg of geno mic DNA was digested with the *Mse*I restriction enzyme (New England BioLabs, Ipswich, M A, USA) to generate DNA fragments containing 5mC or 5-hydroxymethylcytosine (5hmC) m odifications. All primers used in the DNA methylation assay are listed in the key resources table.

***Chromatin immunoprecipitation (ChIP) assay***

ChIP analysis was performed using a Magna ChIP G Kit (Millipore, Billerica, MA, USA) according to the manufacturer's instructions. Cross-linked chromatin isolated from cell extracts (from $1\times10^7$ cells) was sheared by sonication at 40% amplitude (six 20-s pulses separated by 30-s rest intervals on ice) in 500 mL of Nuclear Lysis Buffer and then immunoprecipitated using Protein G magnetic beads conjugated with 1 mg of ChIP-grade target-specific or immunoglobulin (Ig)G control antibodies, which are listed in the key resources table. Enrichment of each histone modification was calculated as the ratio of bound to unbound amplicon fractions. All primers used in the ChIP assay are listed in the key resources table.

***In vitro characterization of ESCs***

Cell viability was determined by the MTT assay (DoGENBio, Seoul, Korea). Alkaline phosphatase (AP) activity in undifferentiated ESCs and established induced pluripotent stem cells (iPSCs) was assessed using an AP Staining Kit II (Reprocell, Yokohama, Japan). The frequency and size distribution of AP-stained PSC colonies were analyzed using a GelCount

colony counter (Oxford Optronix, Abingdon, UK) with default settings.

***Somatic cell reprogramming assay***

Somatic reprogramming was performed using inducible four reprogramming factors (iOSKM) mouse embryonic fibroblasts (MEFs), which harbor a doxycycline (DOX)-inducible polycistronic cassette encoding Oct4, Sox2, Klf4, and Myc (OSKM), as previously described [23]. Briefly, iOSKM MEFs were maintained in high-glucose Dulbecco's Modified Eagle Medium (DMEM) high glucose (HyClone, Pittsburgh, PA, USA) supplemented with 10% fetal bovine serum (FBS) (HyClone) and 0.1% gentamicin (Gibco, Waltham, Waltham, USA). For ectopic expression, MEFs were infected with lentiviruses encoding *Dnmt3L* WT and the mutants in the presence of 6 μg/mL polybrene (Sigma-Aldrich, St. Louis, MO, USA). To initiate reprogramming, DOX (Sigma-Aldrich) was added at a final concentration of 2 μg/mL for 3 days to induce OSKM expression. Cells were subsequently cultured for 18 days in two small molecule inhibitors of Mek1/2 and Gsk3 (PD0325901 and CHIR99021) (2i)- leukemia inhibitory factor (LIF)+FBS reprogramming medium, which was DMEM-high glucose supplemented with 15% FBS, GlutaMAX (Gibco), 1× MEM non-essential amino acids (ME M NEAA) (Gibco), 0.1% gentamicin, 0.1 mM β-mercaptoethanol (Sigma-Aldrich), 3 μM CHIR99021 (BioGems, Westlake Village, CA, USA), and 1 μM PD0325901 (BioGems), in the presence of mouse LIF. At day 21, iPSC colonies were fixed and stained for AP activity using an AP Staining Kit II (Reprocell), and visualized by brightfield microscopy (AMEX1000, Thermo Fisher Scientific).

***Embryoid body (EB) formation***

mESCs used in all lineage differentiation assays were adapted with 2i-LIF- Knockout Serum Replacement (KSR) differentiation medium (85% Knockout DMEM/F-12, 15% KSR, 2 mM

GlutaMAX, 1% MEM non-essential amino acids, 1% penicillin/streptomycin, 1000 U/mL LIF, 3 μM CHIR99021, and 1 μM PD0325901) for three passages before differentiation induction. For the spontaneous differentiation assay, EB spheres were formed using the hanging drop method and Petri dishes, as previously described [24]. For neuronal differentiation, EB spheres were formed with 3000 cells/drop using basal differentiation medium (DMEM/F-12 supplemented with 15% FBS, 0.1 mM b-mercaptoethanol, 1% MEM non-essential amino acid solution, and 1% penicillin/streptomycin solution. For cardiac differentiation, EB spheres were formed with 6000 cells/drop using EB formation medium (FBS-LIF medium without LIF).

### *In vitro neuronal differentiation*

*In vitro* neuronal differentiation was performed as previously reported [25], with slight modifications. Briefly, 2-day-old EB spheres in basal differentiation medium (DMEM/F-12 supplemented with 15% FBS, 0.1 mM b-mercaptoethanol, 1% MEM non-essential amino acid solution, and 1% penicillin/streptomycin solution) were transferred to a gelatin-coated dish with neural inducer medium containing 1 μM all-trans retinoic acid (Sigma-Aldrich) and further cultured for 8 days with medium replacement every 2 days. EBs were then dissociated into single cells, and neural progenitor cells were seeded at a density of 250,000 cells/mL onto 12-well chamber slides containing microscope coverglasses (Marienfeld, Lauda-Königshofen, Germany) pre-coated with 0.1% gelatin. After 6 h, the medium was replaced with N2B27 medium Ⅱ (49% DMEM/F-12 supplemented with 48% Neurobasal medium, 1% N2 supplement, 2% B27 supplement, 2 mM GlutaMAX, and 0.1 mM b-mercaptoethanol), which was refreshed every 2 days for a total of 16 days. Neuron-like cells and their processes were stained with a mouse anti-bIII-tubulin antibody (Millipore) and nuclei were counterstained with DAPI. The neuronal differentiation potency was quantified as bIII-tubulin$^{+}$ cells/DAPI$^{+}$

cells in 24 randomly chosen representative areas from each slide.

### *In vitro cardiac differentiation*

*In vitro* cardiac differentiation was performed as previously reported [25], with slight modifications. Briefly, 2-day-old EB spheres in EB formation medium were collected by inverting the dish lid and gently adding 8 mL of EB formation medium to allow them to settle. The plates were incubated for an additional 2 days with gentle daily tapping to prevent EB aggregation. Eight EBs were transferred to each well of a gelatin-coated 12-well plate containing 1 mL of EB formation medium. Three replicate wells were prepared per condition. The medium was replaced every 2 days starting from the day of EB seeding. For 6 days, beating cardiomyocyte-like cell clusters were identified and recorded using a phase-contrast microscope (AMEX1000) and EP Viewer (Olympus, Tokyo, Japan). After imaging, cells were immediately harvested for downstream RNA extraction.

### *In vitro germline differentiation*

*In vitro* germ cell differentiation was performed as previously reported [26,27]. To detect germline-committed cells, EB spheres that formed from gcOct4-ESCs on the indicated days were fixed with 4% paraformaldehyde (Sigma-Aldrich) for 24 h, embedded in paraffin blocks, and cut into 3 mm-thick sections using a microtome. Green fluorescent protein $(GFP)^+$ (green) germ cells were further analyzed by immunofluorescence (IF) staining using an anti-GFP rabbit IgG polyclonal antibody (Abcam, Cambridge, UK) and an Alexa Fluor 488-conjugated anti-rabbit IgG antibody (Thermo Fisher Scientific). Nuclei were counterstained with DAPI (Sigma-Aldrich). The stained samples were imaged using an inverted fluorescence microscope (EVOSÒ FL Color Imaging System).

To quantify germline-committed $GFP^+$ cells, EB spheres generated from gcOct4-

ESCs on the indicated days were dissociated using StemPro Accutase (Life Technologies, La Jolla, CA) for 10 min at room temperature and resuspended in ESC medium lacking ESGRO/LIF. GFP-expressing cells were analyzed using a BD FACSCanto II flow cytometer (BD Biosciences, San Jose, CA, USA), as previously reported [19,28]. Data were analyzed with FlowJo software 7.6.5 (FlowJo, Ashland, OR, USA).

***Transcriptome analysis***

Total RNA was isolated from teratomas in each group using an RNeasy Mini Kit (Qiagen), including treatment with DNase I (Qiagen). RNA quality was assessed by an Agilent 4200 TapeStation System (Agilent Technologies, Amstelveen, The Netherlands), and RNA was quantified using Invitrogen Qubit 4 (Thermo Fisher Scientific).

Libraries were prepared from total RNA using a CORALL RNA-Seq V2 Library Prep Kit (LEXOGEN, Vienna, Austria). mRNA was isolated using a Poly(A) RNA Selection Kit (LEXOGEN). The isolated mRNA was used for cDNA synthesis and shearing, following the manufacturer's instructions. Indexing was performed using Illumina indexes 1–12. The enrichment step was carried out using PCR. Subsequently, libraries were checked using the TapeStation4200 System or an Agilent 2100 bioanalyzer (Agilent Technologies) to evaluate the mean fragment size. Quantification was performed using a library quantification kit and a StepOne Real-Time PCR System (Life Technologies). High-throughput sequencing was performed as paired-end 100 sequencing using NovaSeq 6000 (Illumina, San Diego, CA, USA).

A quality control of raw sequencing data was performed using FastQC [29]. Adapter and low-quality reads were removed using Fastp [30]. Then, trimmed reads were mapped to the reference genome using STAR [31]. The quantification of reads was processed using Salmon [32]. The read counts were processed based on the trimmed Mean of M-values, counts per million

normalization method using the Python “conorm” package. Data mining and graphic visualization were performed using ExDEGA (Ebiogen Inc., Seoul, Korea) or R (version 4.4.1, R project, Vienna, Austria) and RStudio (version 2024.12.1+563, Posit, Boston, MA, USA) software. For graphical visualization, ggplot2 (version 3.5.3) packages were mainly used.

Basic differentially expressed gene (DEG) analysis was conducted using DESeq2 packages (version 1.44.0). Functional analysis of transcriptomes and core analyses of gene networks, biofunctions, and canonical pathways were performed using MetaCore (Clarivate Analytics, Philadelphia, PA, USA) or gene set enrichment analysis (GSEA) software (Broad Institute, Cambridge, MA, USA). For GSEA, gene sets were obtained from published literature or filtered from a curated functional gene set (C2) and gene ontology (GO) gene set (C5) database from MSigDB [33]. Significant differences were determined based on a false discovery rate <0.25. A detailed list of gene sets with the corresponding genes and references used for GSEA is described in **Source datasets**. GO analysis was performed using clusterProfiler (version 4.12.6) packages [34].

***In silico prediction of Dnmt3L-interacting E3 ligases***

Protein–protein interaction candidates for Dnmt3L were predicted using two independent public databases, BioGRID and UbiBrowser. BioGRID analysis was performed using the BioGRID database (https://thebiogrid.org; version: 5.0), which curates experimentally validated protein–protein interactions from published literature. The human Dnmt3L gene (DNMT3L) was queried, and all searches were conducted using the default search parameters and filtering settings provided by the database at the time of analysis.

In parallel, ubiquitination-related interaction predictions were analyzed using UbiBrowser (http://ubibrowser.bio-it.cn/ubibrowser_v3; version: 2.0), an integrated web-based platform for predicting E3 ligase–substrate interactions based on multi-omics features

and network-based algorithms. DNMT3L was used as the input substrate, and all predictions were generated using the default algorithm settings and confidence thresholds provided by the platform at the time of analysis.

## Key resources table

| Antibodies | | |
|---|---|---|
| **Reagent or Resource** | **Source** | **Identifier** |
| 5-Hydroxymethylcytosine (5-hmC) | Active motif | 39769<br>PRID: AB_10013602 |
| 5-Methylcytosine (5-mC) | Active motif | 61479<br>PRID: AB_2793653 |
| Acetylated-Lysine (Ac-K-103) | Cell Signaling Technology | 9681 |
| Alexa Fluor™ 488 | Invitrogen™ | A11001 / A11008 |
| Alexa Fluor™ 546 | Invitrogen™ | A11060 |
| ANTI-FLAG® M2 | Sigma-Aldrich | F3165 |
| Anti-GFP | Abcam | ab290 |
| Anti-Klf2 | Sigma-Aldrich | 09-820 |
| Anti-Phosphoserine, clone 4A4 | Sigma-Aldrich | 05-1000 |
| Anti-SOX2 antibody [EPR3131] | Abcam | ab92494 |
| Anti-Stra8 [EPR27083-56] | Abcam | ab308124 |
| Anti-β-Actin (ACTB) | Sigma | A5441 |
| Anti-β-Tubulin III | Sigma | MAB1637 |
| BMP4 | ABclonal | A11315<br>PRID: AB_2758503 |
| Cardiac Troponin I (TNNI3) | ABclonal | A6995<br>PRID: AB_2767551 |
| Cardiac troponin T (TNNT2) | ABclonal | A4914<br>PRID: AB_2863388 |
| DAPI ready made solution | Sigma-Aldrich | MBD0015 |
| DNMT1 (D63A6) XP® | Cell Signaling Technology | 5032 |
| DNMT3A (D23G1) | Cell Signaling Technology | 3598 |
| DNMT3A (E9P2F) | Cell Signaling Technology | 49768 |
| DNMT3B (E9X7R) | Cell Signaling Technology | 44145 |
| DNMT3B Rabbit pAb | ABclonal | A11079 |
| DNMT3L (E1Y7Q) | Cell Signaling Technology | 13451 |
| DNMT3L Rabbit pAb | ABclonal | A2342 |
| G9a/EHMT2 (C6H3) | Cell Signaling Technology | 3306 |
| HA-probe (Y-11) | Santa Cruz | sc-805 |
| K48-linkage Specific Polyubiquitin (D9D5) Rabbit mAb | Cell signaling Technology | 8081 |
| K63-linkage Specific Polyubiquitin (D7A11) Rabbit mAb | Cell signaling Technology | 5621 |
| KLF4 | Cell Signaling Technology | 4038 |
| NeuN | ABclonal | A19086<br>PRID: AB_2862578 |
| Normal mouse IgG | Santa Cruz | sc-2025 |
| Oct-3/4 (C-10) | Santa Cruz | sc-5279 |
| Peroxidase AffiniPure® Goat Anti-Rabbit IgG (H+L) | Jackson ImmunoResearch | 111-035-045<br>PRID: AB_2337938 |
| Peroxidase AffiniPure® Rabbit Anti-Mouse IgG (H+L) | Jackson ImmunoResearch | 315-035-045<br>PRID: AB_2340066 |
| Phospho-Threonine/Tyrosine | Cell Signaling Technology | 9381 |
| PRDM14 | R&D systems | MAB8097 |
| SIRT1 (H-300) | Santa Cruz | sc-15404 |
| TFCP2L1 | Aviva Systems Biology | OAAB09732 |
| UHRF1 (D6G8E) | Cell Signaling Technology | 12387 |

| Oligonucleotides_qRT primers for murine | | |
|---|---|---|
| **Genes** | **Forward (5' to 3')** | **Reverse (5' to 3')** |
| Gapdh | AGGTCGGTGTGAACGGATTTG | AGGTCGGTGTGAACGGATTTG |
| *Apela* | ATGCGATTCCAGCCCCTTT | GAAGGGCACTCGAGAATGAAGT |
| *Ascl* | AAGCACACCTTGACTGGTACG | TGAAGGTGCAAACGTCCACTT |
| *Atoh1* | ATCCCGTCCTTCAACAACGAC | ATCCCGTCCTTCAACAACGAC |
| *Cer1* | CAACCACGAGGAGGCAGAAG | ATCGCTTTCCACATCCCTTGG |
| *C-Myc* | ACCAGCAGCGACTCTGAAGAAG | GTTTGCCTCTTCTCCACAGACA |
| *Corin* | CCAATGAAGATGTGGAAGAATGC | AGCCTGGCCGTCACATCTC |
| *Dazl* | CAACTGTTAACTACCACTGCAG | CAAGAGACCACTGTCTGTATGC |
| *Dlk1* | GATTCTGCGAGGCTGACAATG | CCAGGGGCAGTTACACACTTG |
| *Dnah11* | GAACATGCCTGAAGTGGATTTG | GATCGTACCAGTGCCCATAGTCA |
| *Dnmt3B* | CTTCGAGTTTTACCACTTGCTGAA | CCACAACATTCTCGAACATCCA |
| *Dnmt1* | CTGCAAGGACATGAGCCCAC | CCTGTATGTTGGGCAGGTCAC |
| *Dnmt3A* | TTCTTGAGTCTAACCCCGTGATG | CCAGGAAGGTTACCCCAGAAG |
| *Dnmt3L* | CACCAGAGTTACTGCACCATCTG | ACACTCGAAACAGTAGCATCTGGTA |
| *Efnb3* | CCTGCCCCAAACCTTCTTCT | GGGCTGTATTCCTGGAACTTGA |
| *Esrrb* | TGGGCCTAGCAGGGTCAGA | TGCCACCTGTTTCTCATGAGTAG |
| *Fgf8* | TGGCCAACAAGCGCATCA | TCCAAAAGTATCGGTCTCCACAAT |
| FLAG-*Dnmt3L* | CGGGCTGCAGGAATTCATG | GGTCCAAGGTTTCAAGGGTCTT |
| *Folr1* | GATGGCCGAATGTGCTCAGT | GTTTTTCTTTGTGGTGTTTGGCAT |
| *Foxa1* | TGGACTTCAAGGCATACGAGC | GCACGGGTCTGGAATACACA |
| *G9a* | CGGAAAACCATGTCCAAACC | ATGCGGAAATGCTGGACTTC |
| *Gfap* | CTGGAGGTGGAGAGGGACAA | CAGCCTCAGGTTGGTTTCATC |
| *Kitl* | GAAGACTCGGGCCTACAATGG | AAAGCCAATTACAAGCGAAATGA |
| *Klf2* | GAAGTTCGCGCGCTCTGA | TCGCACAAGTGGCACTGAA |
| *Klf4* | GGCGAGTCTGACATGGCTG | GCTGGACGCAGTGTCTTCTC |
| *Meg3* | TCCTCACCTCCAATTTCCCCT | GAGCGAGAGCCGTTCGATG |
| *Mvh* | GGCAAAGAAAAGATTGGCCT | GGGTTTGGCGTTGTTCCTT |
| *Nanog* | TTCTTGCTTACAAGGGTCTGC | AGAGGAAGGGCGAGGAGA |
| *Nectin3* | TTCGTGGGAAGAAAAGGTGTTAA | AACCTGCTCCACACGGACTT |
| *Nestin* | CCCTGAAGTCGAGGAGCTGG | AGCTGCTGCACCTCTAAGCGA |
| *Neurog1* | GGCTTCAGAAGACTTCACCTATGG | TCGTGTGGAGCAGGTCTTTG |
| *Nkx2.2* | ACAACCCCTACACTCGCTGG | GTCATTGTCCGGTGACTCGTC |
| *Ntn1* | AGAACGAAGACGACTCGGAGCT | GCCGTGTTGTGCCTACAGTCA |
| *Oct4* | ACATCGCCAATCAGCTTGG | AGAACCATACTCGAACCACATCC |
| *Pkd1l1* | TGTCAGCTCCACTACCCTAAGAAA | TTCGCAGGCCCCATGTT |
| *Pla2g1b* | GTGTGGCAGTTCCGCAATATG | CCTGTCTAAGTCGTCCACTGG |
| *Prdm14* | AGTCCCAGGACAAGGCGAAC | GGTGGTGGCGAGGTTCCTAA |
| *Rbp4* | CCACTGGATCATCGACACGG | GCCATTGGGGTCACGAGAA |
| *S100b* | TGGTTGCCCTCATTGATGTCT | CCCATCCCCATCTTCGTCC |
| *Serpinb6c* | TGGTCATGGTCTTATTGGGGG | AGACAGTGCCTGAGTTATCTGG |
| *Sirt1* | GTCATGGTTCCTTTGCAACAGC | GGCACCGAGGAACTACCTGATT |
| *Slit2* | AGCGGATTATCTCCACACCAA | CGGAATTTCTTGCTTTTGATCTG |
| *Snrpn* | TGCAAAACAGCCAGAACGTGA | ACACGAGCAATGCCAGTATCTTT |
| *Sohlh2* | GGGCAGGGCAGAGTAAATCTT | CAAACGAGTTAGCAGCCAAAAG |
| *Sox2* | GCTCGCAGACCTACATGAACG | GCCTCGGACTTGACCACAGA |
| *Stra8* | ACAACCTAAGGAAGGCAGTTTAC | GACCTCCTCTAAGCTGTTGGG |
| *Sycp3* | TTGGAGCTGACATCAACAAAG | CCACTGCTGCAACACATTCAT |
| *Tfcp2l1* | GGCCTCCGTGAAGGTAACAG | GCTGATGGGAGCAGGTGATC |

| | | |
|---|---|---|
| *Uhrf1* | GCTCCAGTGCCGTTAAGACC | CACGAGCACGGACATTCTTG |
| *Vtn* | CGGAATTTCTTGCTTTTGATCTG | GGCTTGCACTGCTCCATGTA |
| **Oligonucleotides_meDIP, ChIP and BSS sequencing primers for murine** | | |
| **Genes** | **Forward (5' to 3')** | **Reverse (5' to 3')** |
| *Cer1* | TGCCTCCCCATGCTACCA | GCAAGAGAACAAGCAGCTGAAC |
| *Foxa1* | GCTCGGCTGACTCAGATGACT | TCCGCTCTTGGTCCACTTG |
| *Snrpn* | GCAACCCTATAGGTGAAACAACAA | TAGCCCTGGCGTTACCCTGTA |
| *Stra8* | GGCAGTGACAGGGCTGTGAT | ACGCAGGAAACTGCACGAA |
| *Tfcp2l1* | TGACTAACCTCAGGCCTCCA | GGAGAGGAAAGGCGACGAAA |
| *Stra8*_BSS | ATGTATTTTTATTGGTTATGGTGGTAGT | ATATCCACTAACAAAACATACATTCAC A |

| **Sequences of guide RNAs and donor template for gene editing** | |
|---|---|
| Tevopreq1 linker for K238R | TCAAATAT |
| Spacer for K238R | CCTTCCCCCCAGTACTAAAG |
| Reverse transcription template for K238R | AAGCCCAAACTCCT |
| Primer binding site for K238R | TAGTACTGGGGGA |
| Nicking guide RNA spacer for K238R | CCCCAGTACTAAGGAGTTTG |
| Spacer for K412R | ACTTGAAGTACTCTCTCAGC |
| Scaffold | GTTTTAGAGCTAGAAATAGCAAGTTAAAATAAGGC TAGTCCGTTATCAACTTGAAAAAGTGGCACCGAGT CGGTGC |
| ssODN | CAAGTCAGAAGCAGGAGCAAGCTGGACGCCCCGA AAGTTGACCTCCTGGTGAAGAACTGCCTTCTGCCGC TGAGAGAGTACTTCAGGTATTTTTCTCAAAACTC*A *C*T<br>* indicates phosphorothioate linkage |

| **Bacterial and virus strain** | | |
|---|---|---|
| **Reagent or Resource** | **Source** | **Identifier** |
| pLKO.1-shPrdm14 (TRC2) | Sigma-Aldrich (MISSION® TRC2) | TRCN0000238859 (TTAAGTCGTCCCAGTCAATAT) |
| **Biological sample** | | |
| **Reagent or Resource** | **Source** | **Identifier** |
| Teratoma tissue from R1 mESCs | This page | IACUC 2021-12-271 |

| **Compounds** | | |
|---|---|---|
| **Reagent or Resource** | **Source** | **Identifier** |
| (R)-MG 132 | TOCRIS | 6033 |
| 1,4-Dithiothreitol | Roche | DTT-RO |
| 2-Mercaptoethanol | Sigma-Aldrich | M6250 |
| 3xFLAG™ Peptide | Sigma-Aldrich | F4799 |
| 4% Paraformaldehyde Solution | Biosesang | P2031-100-00 |
| Actinomycin D | Sigma-Aldrich | A9415 |
| Anti-FLAG® M2 Magnetic Beads | Millipore | M8823 |
| Anti-HA−Agarose antibody | Millipore | A2095 |
| ART-558 | MedChemExpress | HY-141520 |
| AZD-7648 | MedChemExpress | HY-11178 |
| BIX01294 | Selleckchem | S8006 |
| Characterized Fetal Bovine Serum (FBS) | HyClone™ | SV30207.02 |
| CHIR 99021 | Biogems | 2520691 |
| cOmplete™ ULTRA Tablets, Mini, EDTA-free, EASYpack Protease Inhibitor Cocktail | Roche | 05892791001 |
| Cycloheximide | Sigma-Aldrich | C7698 |

| | | |
|---|---|---|
| DMEM/F-12 | Gibco™ | 11320-033 |
| DMEM/F-12, GlutaMAX™ supplement | Gibco™ | 10565-018 |
| Doxycycline hyclate | Sigma-Aldrich | D9891 |
| DMEM with high glucose | HyClone™ | SH30243.01 |
| ESGRO® Recombinant Mouse LIF Protein | Sigma-Aldrich | ESG1107 |
| GlutaMAX™ Supplement | Gibco™ | 35050061 |
| Go 6983 | TOCRIS | 2285 |
| HEPES (1M) | Gibco™ | 15630-080 |
| Hexadimethrine bromide (Polybrene) | Sigma-Aldrich | H9268 |
| HyClone™ HyPure Water, Cell Culture Grade | HyClone™ | SH30529.02 |
| HyClone™ HyPure Water, Molecular Biology Grade | HyClone™ | SH30538.02 |
| IGEPAL® CA-630 | Sigma-Aldrich | I3021 |
| KnockOut™ Serum Replacement | Gibco™ | 10828-028 |
| L-Glutamine (200 mM) | Gibco™ | 25030-081 |
| Lipofectamine™ 2000 Transfection Reagent | Invitrogen™ | 11668500 |
| MEM Non-Essential Amino Acids Solution (100X) | Gibco™ | 11140-050 |
| $MgCl_2$ (1M) | Invitrogen™ | AM9530G |
| Monoclonal Anti-HA-Agarose antibody bead produced in mouse (clone HA-7) | Millipore | A2095 |
| MseI | New England Biolabs | R0525 |
| Opti-MEM™ I Reduced Serum Medium | Gibco™ | 31985-070 |
| PD 0325901 | Biogems | 3911091 |
| Penicillin-Streptomycin (10,000 U/mL) | Gibco™ | 15140-122 |
| PhosSTOP | Roche | 04906837001 |
| Proteinase K Solution, ChIP grade | Thermo Scientific™ | 26160 |
| Puromycin Dihydrochloride | Gibco™ | A11138-03 |
| QIAGEN Proteinase K (10 mL) | QIAGEN | 19134 |
| Retinoic acid | Sigma | R2625 |
| RNase-Free DNase Set (50) | QIAGEN | 79254 |
| Sodium Butyrate | Sigma-Aldrich | 19-137 |
| Triton™ X-100 | Sigma-Aldrich | T8787 |
| TrypLE™ Express Enzyme (1X), phenol red | Gibco™ | 12605-010 |
| UltraPure™ 0.5M EDTA, pH 8.0 | Invitrogen™ | 15575020 |
| **Critical commercial assays** | | |
| **Reagent or Resource** | **Source** | **Identifier** |
| ChIP DNA Clean & Concentrator | ZYMO RESEARCH | D5205 |
| DNeasy Blood & Tissue Kit (250) | QIAGEN | 69506 |
| Exprep™ Plasmid SV | GeneAll | 101-102 |
| EZ-cytox Cell Viability Assay Kit | DoGENBio | EZ-3000 |
| EZ DNA Methylation-Gold Kit | ZYMO RESEARCH | D5005 |
| Magna ChIP G (2-8C) | Sigma-Aldrich | MAGNA0002 |
| MinElute PCR Purification Kit (50) | QIAGEN | 28004 |
| Muta-Direct™ Site Directed Mutagenesis Kit | iNtRON | 15071 |
| NaveniFlex Cell MR Red [kit/100rxn] | Navinci | NVC_60025 |
| Plasmid Midi Kit (100) | QIAGEN | 12145 |
| pLUG-Prime® TA-cloning Vector Kit II | iNtRON | 11063 |
| QIAquick Gel Extraction Kit (250) | QIAGEN | 28706 |
| QIAquick PCR Purification Kit (50) | QIAGEN | 28104 |
| RNeasy Mini Kit (250) | QIAGEN | 74106 |
| StemAb™ Alkaline Phosphatase Staining Kit II | Reprocell | 00-0055 |
| ZymoTaq DNA Polymerase | ZYMO RESEARCH | E2002 |

| **Deposited data** | | |
|---|---|---|
| **Reagent or Resource** | **Source** | **Identifier** |
| Bulk RNA-seq transcriptome data | This page | GSE298928 |
| ChIP-seq datasets of Dnmt3L and H3K27me3 | Baubec, T. et al.[43], Subramanian, V. et al.[54] | GSE57413, GSE40065 |
| Grayscale Converter v1.0 | N.A. | https://github.com/chfhrqlc/GrayScale_Converter.git |
| Mass spectrometry proteomics data | This page | PXD032953 https://www.ebi.ac.uk/pride/archive/projects/PXD032953 |
| **Experimental models: Cell lines** | | |
| **Reagent or Resource** | **Source** | **Identifier** |
| 293FT Cell Line | Invitrogen™ | R70007 |
| iOSKM MEFs | Koo, K. M. et al.[27] | N.A. |
| R1 | ATCC | SCRC-1011™ |
| **Experimental models: Organisms/strains** | | |
| **Reagent or Resource** | **Source** | **Identifier** |
| Mouse: eight-week-old male NOD-Prkdc$^{EM1}$ mice | JA BIO | N.A. |
| **Recombinant DNA** | | |
| **Reagent or Resource** | **Source** | **Identifier** |
| BLOCK-iT™ Lentiviral RNAi Expression System | Invitrogen™ | K494400 |
| pcDNA3-CKCHA | Kyung Chul, Choi | N.A. |
| pCMV_3Tag-1 vector | Agilent | 240195 |
| pENTR4 plasmid | Invitrogen™ | A10465 |
| pEZ-Lv235 expression vector for LxR recombination cloning (lentiviral) | GeneCopoeia | EZ016 |
| pRK5-HA-Ubiquitin-WT | addgene | 17608 |
| pRK5-HA-Ubiquitin-K48R | addgene | 17604 |
| **Software and algorithms** | | |
| **Reagent or Resource** | **Source** | **Identifier** |
| Adobe Photoshop | Adobe Systems | RRID:SCR_014199 |
| BioRender | BioRender | RRID:SCR_018361 |
| clusterProfiler (version 4.12.6) | Xu, S. et al. | https://bioconductor.org/packages/release/bioc/html/clusterProfiler.html |
| DESeq2 (version 1.44.0) | Love MI, Huber W, Anders | https://bioconductor.org/packages/release/bioc/html/DESeq2.html |
| ggplot2 (version 3.5.3) | H. Wickham | https://ggplot2.tidyverse.org/ |
| GraphPad Prism 7 | GraphPad Software | RRID:SCR_002798 |
| ImageJ | Schneider et al. | https://imagej.nih.gov/ij/ |
| QUMA | RIKEN | http://quma.cdb.riken.jp/ |
| R (version version 4.4.1) | R Foundation for Statistical Computing | RRID:SCR_001905 |
| RStudio (version 2024.12.1+563) | Posit team | http://www.posit.co/ |

## Uncropped WB

**Fig. 1**

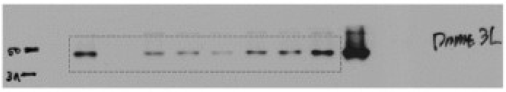

**Fig. 1b**
**(2i-LIF , Dnmt3L WB)**

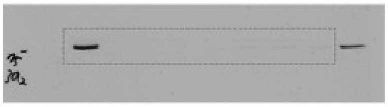

**Fig. 1b**
**(2i-LIF , Dnmt3A2 WB)**

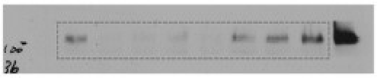

**Fig. 1b**
**(2i-LIF , Dnmt3B WB)**

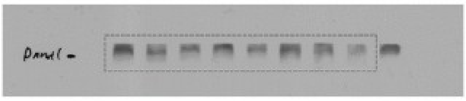

**Fig. 1b**
**(2i-LIF , Dnmt1 WB)**

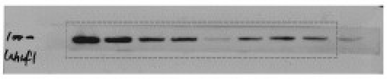

**Fig. 1b**
**(2i-LIF , Uhrf1 WB)**

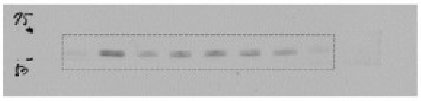

**Fig. 1b**
**(2i-LIF , Tfcp2l1 WB)**

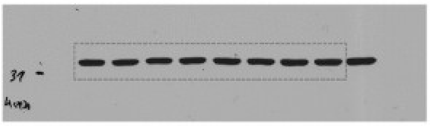

**Fig. 1b**
**(2i-LIF , β-Actin WB)**

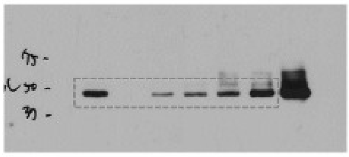

**Fig. 1b**
**(2i-LIF-KSR , Dnmt3L WB)**

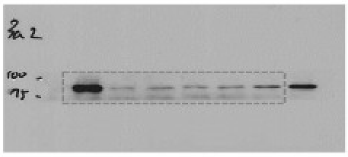

**Fig. 1b**
**(2i-LIF-KSR , Dnmt3A2 WB)**

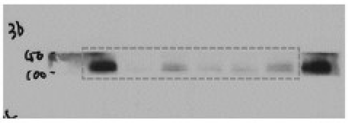

**Fig. 1b**
**(2i-LIF-KSR , Dnmt3B WB)**

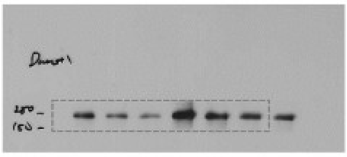

**Fig. 1b**
**(2i-LIF-KSR , Dnmt1 WB)**

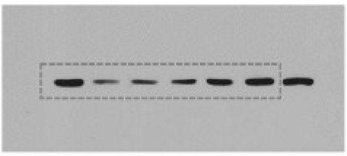

**Fig. 1b**
**(2i-LIF-KSR , Uhrf1 WB)**

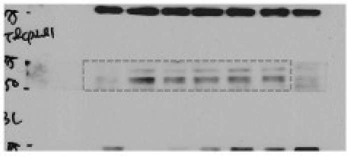

**Fig. 1b**
**(2i-LIF-KSR , Tfcp2l1 WB)**

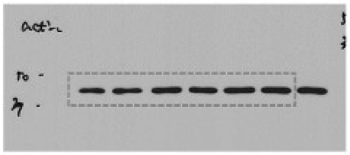

**Fig. 1b**
**(2i-LIF-KSR , β-Actin WB)**

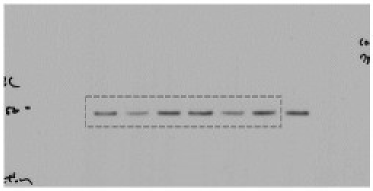

**Fig. 1b**
**(FBS-LIF , Dnmt3L WB)**

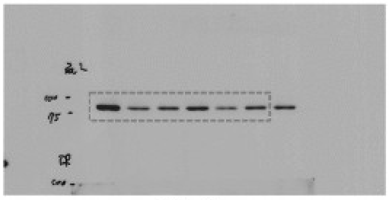

**Fig. 1b**
**(FBS-LIF , Dnmt3A2 WB)**

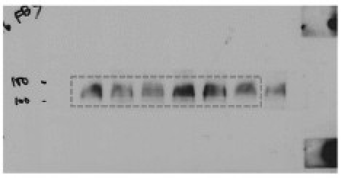

**Fig. 1b**
**(FBS-LIF , Dnmt3B WB)**

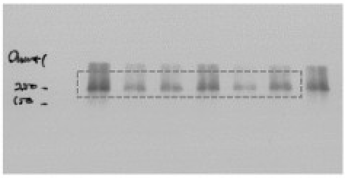

**Fig. 1b**
**(FBS-LIF , Dnmt1 WB)**

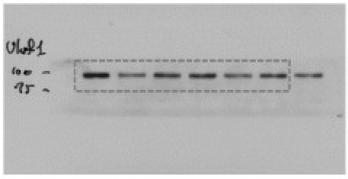

**Fig. 1b**
**(FBS-LIF , Uhrf1 WB)**

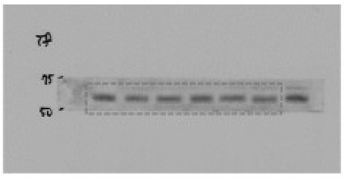

**Fig. 1b**
**(FBS-LIF , Tfcp2l1 WB)**

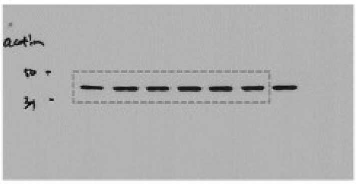

**Fig. 1b**
**(FBS-LIF , β-Actin WB)**

Fig. 1

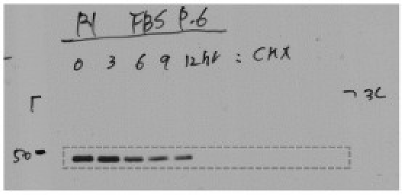

Fig. 1f
(Dnmt3L (s.e) WB)

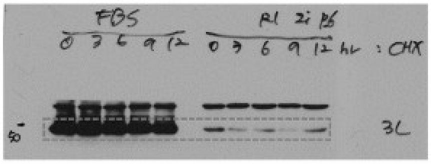

Fig. 1f
(Dnmt3L (l.e) WB)

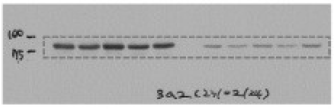

Fig. 1f
(Dnmt3A2 (s.e) WB)

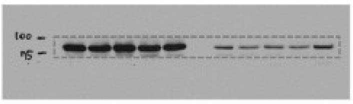

Fig. 1f
(Dnmt3A2 (l.e) WB)

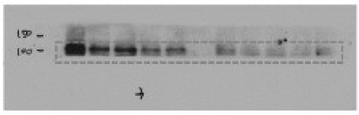

Fig. 1f
(Dnmt3B WB)

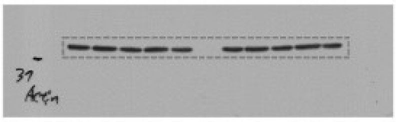

Fig. 1f
(β-Actin WB)

Fig.2

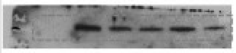

Fig. 2d
(Top panel)
(FLAG-IP, Ac-K WB)

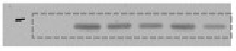

Fig. 2d
(Top panel)
(FLAG-IP, FLAG WB)

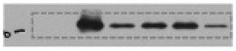

Fig. 2d
(Top panel)
(Input, FLAG WB)

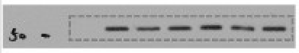

Fig. 2d
(Bottom panel)
(FLAG-IP, Ac-K WB)

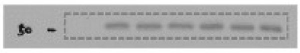

Fig. 2d
(Bottom panel)
(FLAG-IP, FLAG WB)

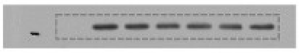

Fig. 2d
(Bottom panel)
(Input, FLAG WB)

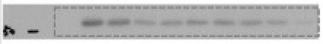

Fig. 2f
(FLAG (s.e) WB)

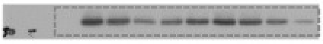

Fig. 2f
(FLAG (l.e) WB)

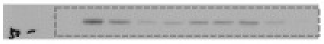

Fig. 2f
(Dnmt3L (s.e) WB)

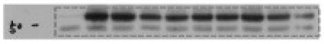

Fig. 2f
(Dnmt3L (l.e) WB)

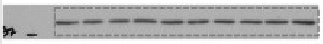

Fig. 2f
(β-Actin WB)

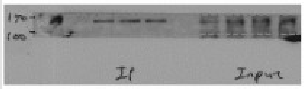

Fig. 2g
(Dnmt3A1 WB)

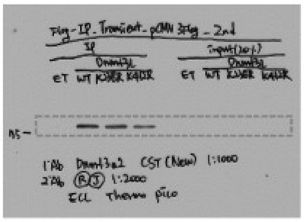

Fig. 2g
(Dnmt3A2 (s.e) WB)

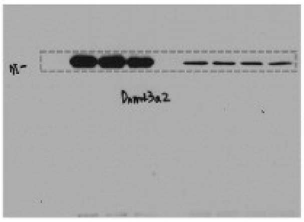

Fig. 2g
(Dnmt3A2 (l.e) WB)

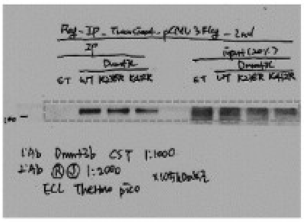

Fig. 2g
(Dnmt3B WB)

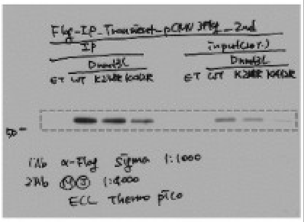

Fig. 2g
(FLAG WB)

Fig. 4

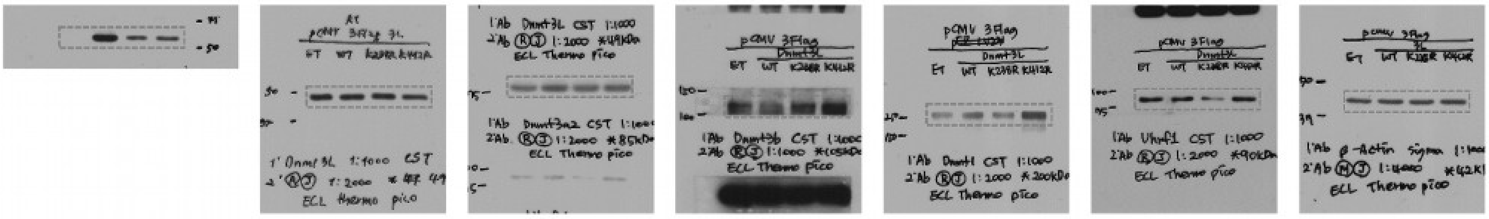

Fig. 4a (FLAG WB) | Fig. 4a (Dnmt3L WB) | Fig. 4a (Dnmt3A2 WB) | Fig. 4a (Dnmt3B WB) | Fig. 4a (Dnmt1 WB) | Fig. 4a (Uhrf1 WB) | Fig. 4a (β-Actin WB)

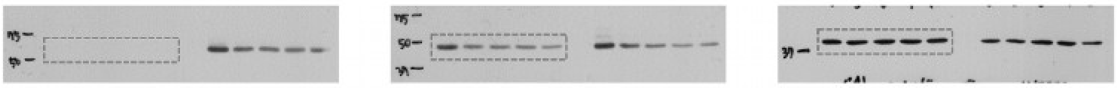

Fig. 4b (Top panel-ET, FLAG WB) | Fig. 4b (Top panel-ET, Dnmt3L WB) | Fig. 4b (Top panel-ET, β-Actin WB)

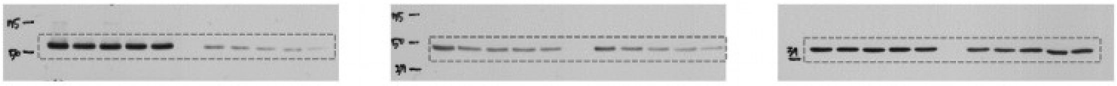

Fig. 4b (Top panel-WT/K238R, FLAG WB) | Fig. 4b (Top panel-WT/K238R, Dnmt3L WB) | Fig. 4b (Top panel-WT/K238R, β-Actin WB)

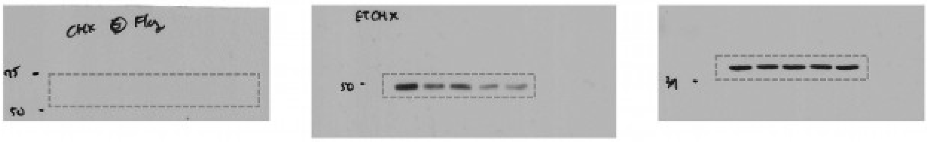

Fig. 4b (Bottom panel-ET, FLAG WB) | Fig. 4b (Bottom panel-ET, Dnmt3L WB) | Fig. 4b (Bottom panel-ET, β-Actin WB)

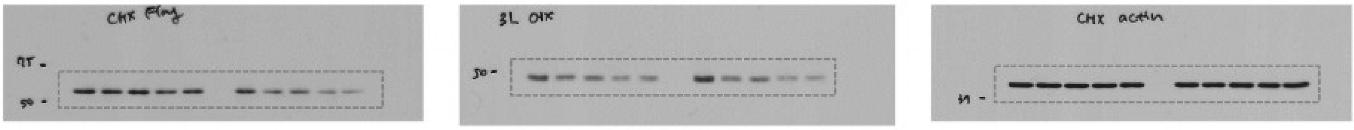


Fig. 4b (Bottom panel-WT/K412R, FLAG WB) | Fig. 4b (Bottom panel-WT/K412R, Dnmt3L WB) | Fig. 4b (Bottom panel-WT/K412R, β-Actin WB)

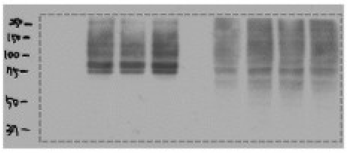

Fig. 4c (HA WB)

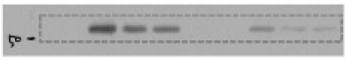

Fig. 4c (FLAG WB)

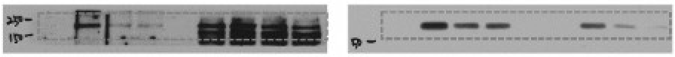

Fig. 4d (Upper panel) (FLAG-IP, HA WB) | Fig. 4d (Upper panel) (FLAG-IP, FLAG WB)

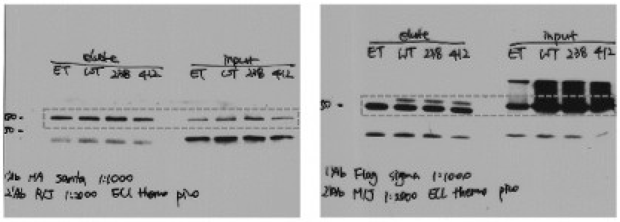


Fig. 4d (Bottom panel) (HA-IP, HA WB) | Fig. 4d (Bottom panel) (HA-IP, FLAG WB)

**Fig. 4**

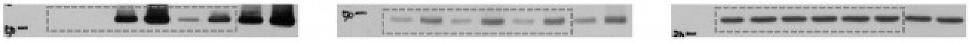

**Fig. 4e (Top panel-FLAG WB)**

**Fig. 4e (Top panel-Dnmt3L WB)**

**Fig. 4e (Top panel- β-Actin WB)**

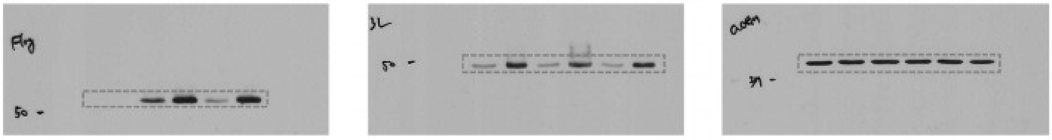

**Fig. 4e (Bottom panel-FLAG WB)**

**Fig. 4e (Bottom panel-Dnmt3L WB)**

**Fig. 4e (Bottm panel- β-Actin WB)**

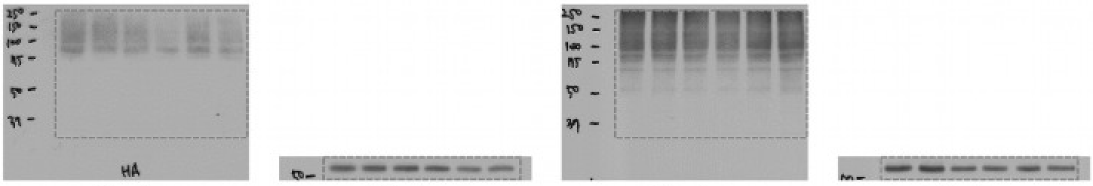

**Fig. 4g (FLAG-IP, HA WB)**

**Fig. 4g (FLAG-IP, FLAG WB)**

**Fig. 4g (Input, HA WB)**

**Fig. 4g (Input, FLAG WB)**

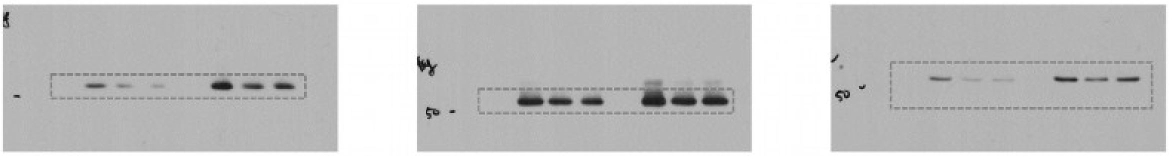

**Fig. 4i (FLAG (s.e) WB)**

**Fig. 4i (FLAG (l.e) WB)**

**Fig. 4i (Dnmt3L (s.e) WB)**

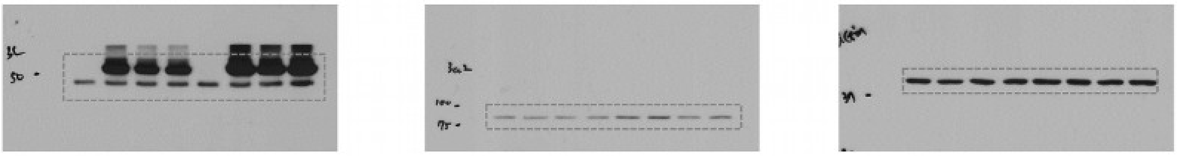

**Fig. 4i (Dnmt3L (l.e) WB)**

**Fig. 4i (Dnmt3A2 WB)**

**Fig. 4i (β-Actin WB)**

**Fig. 5**

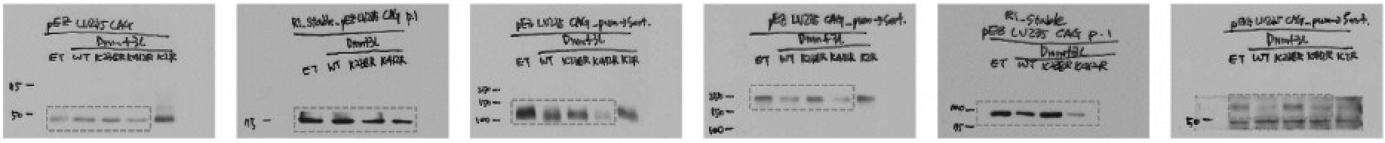

**Fig. 5a (p.1, Dnmt3L WB)**

**Fig. 5a (p.1, Dnmt3A2 WB)**

**Fig. 5a (p.1, Dnmt3B WB)**

**Fig. 5a (p.1, Dnmt1 WB)**

**Fig. 5a (p.1, Uhrf1 WB)**

**Fig. 5a (p.1, Tfcp2l1 WB)**

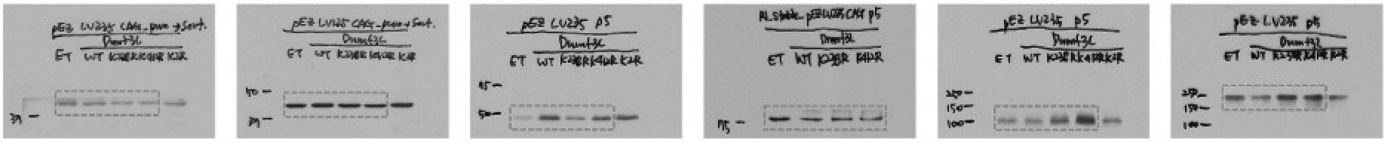

**Fig. 5a (p.1, Sox2 WB)**

**Fig. 5a (p.1, β-Actin WB)**

**Fig. 5a (p.5, Dnmt3L WB)**

**Fig. 5a (p.5, Dnmt3A2 WB)**

**Fig. 5a (p.5, Dnmt3B WB)**

**Fig. 5a (p.5, Dnmt1 WB)**

**Fig. 5**

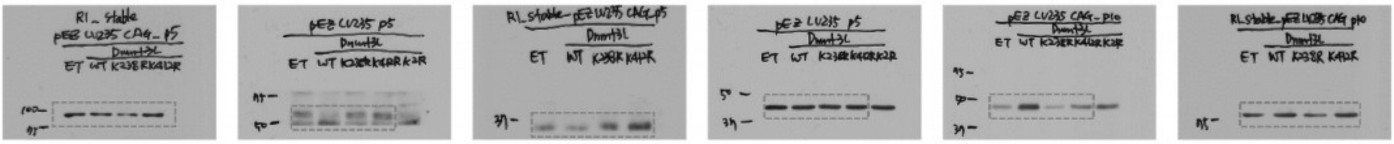

Fig. 5a (p.5, Uhrf1 WB) | Fig. 5a (p.5, Tfcp2l1 WB) | Fig. 5a (p.5, Sox2 WB) | Fig. 5a (p.5, β-Actin WB) | Fig. 5a (p.10, Dnmt3L WB) | Fig. 5a (p.10, Dnmt3A2 WB)

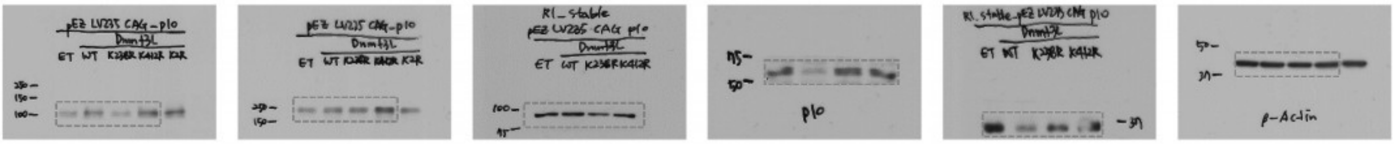

Fig. 5a (p.10, Dnmt3B WB) | Fig. 5a (p.10, Dnmt1 WB) | Fig. 5a (p.10, Uhrf1 WB) | Fig. 5a (p.10, Tfcp2l1 WB) | Fig. 5a (p.10, Sox2 WB) | Fig. 5a (p.10, β-Actin WB)

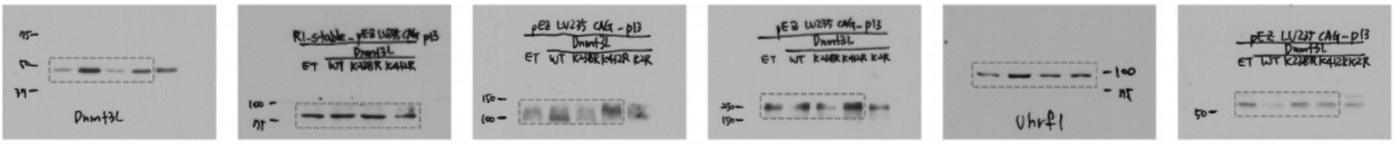

Fig. 5a (p.13, Dnmt3L WB) | Fig. 5a (p.13, Dnmt3A2 WB) | Fig. 5a (p.13, Dnmt3B WB) | Fig. 5a (p.13, Dnmt1 WB) | Fig. 5a (p.13, Uhrf1 WB) | Fig. 5a (p.13, Tfcp2l1 WB)

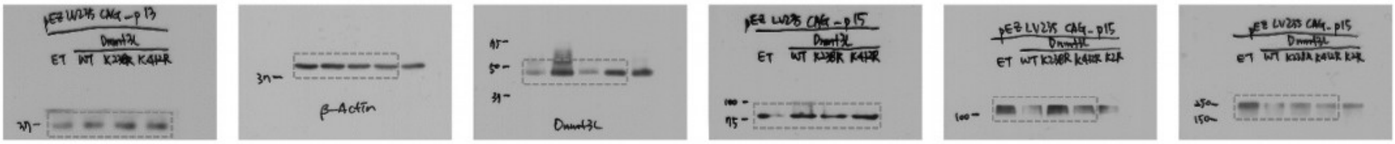

Fig. 5a (p.13, Sox2 WB) | Fig. 5a (p.13, β-Actin WB) | Fig. 5a (p.15, Dnmt3L WB) | Fig. 5a (p.15, Dnmt3A2 WB) | Fig. 5a (p.15, Dnmt3B WB) | Fig. 5a (p.15, Dnmt1 WB)

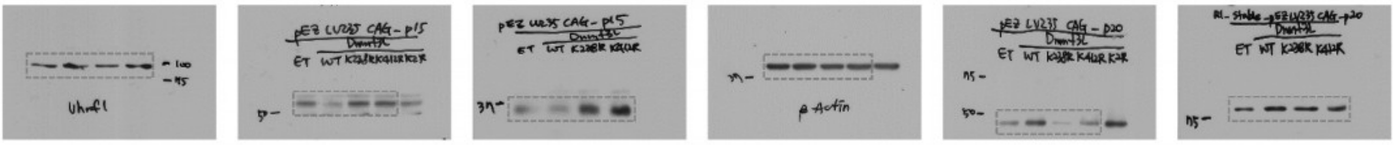

Fig. 5a (p.15, Uhrf1 WB) | Fig. 5a (p.15, Tfcp2l1 WB) | Fig. 5a (p.15, Sox2 WB) | Fig. 5a (p.15, β-Actin WB) | Fig. 5a (p.20, Dnmt3L WB) | Fig. 5a (p.20, Dnmt3A2 WB)

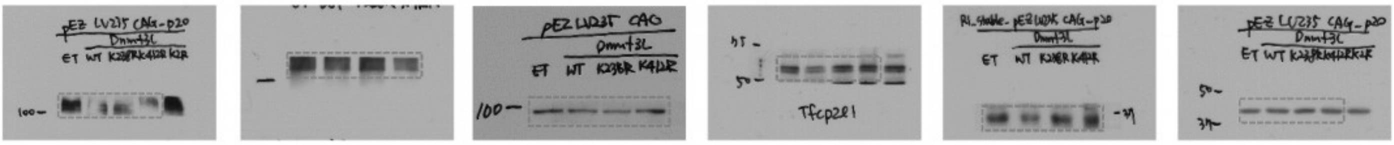

Fig. 5a (p.20, Dnmt3B WB) | Fig. 5a (p.20, Dnmt1 WB) | Fig. 5a (p.20, Uhrf1 WB) | Fig. 5a (p.20, Tfcp2l1 WB) | Fig. 5a (p.20, Sox2 WB) | Fig. 5a (p.20, β-Actin WB)

**Supplementary Fig. 1**

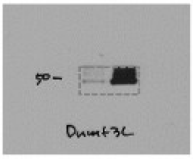


Supplementary Fig. 1b
(2i-LIF, Dnmt3L WB)

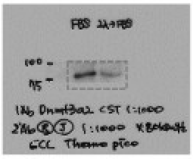

Supplementary Fig. 1b
(2i-LIF, Dnmt3A2 WB)

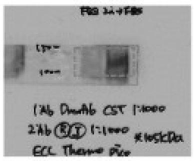

Supplementary Fig. 1b
(2i-LIF, Dnmt3B WB)

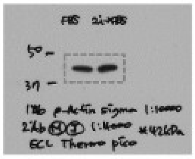

Supplementary Fig. 1b
(2i-LIF, β-Actin WB)

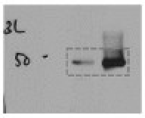

Supplementary Fig. 1b
(2i-LIF-KSR, Dnmt3L WB)

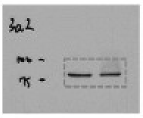

Supplementary Fig. 1b
(2i-LIF-KSR, Dnmt3A2 WB)

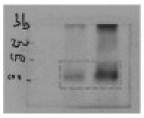

Supplementary Fig. 1b
(2i-LIF-KSR, Dnmt3B WB)

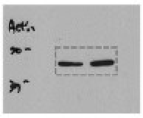

Supplementary Fig. 1b
(2i-LIF-KSR, β-Actin WB)

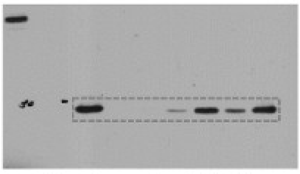

Supplementary Fig. 1d
(Dnmt3L WB)

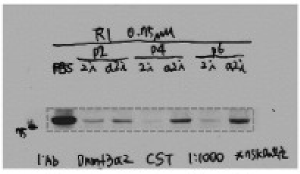

Supplementary Fig. 1d
(Dnmt3A2 WB)

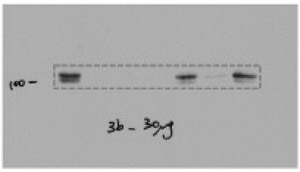

Supplementary Fig. 1d
(Dnmt3B WB)

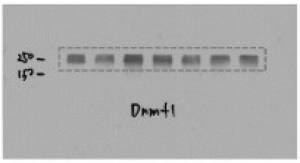


Supplementary Fig. 1d
(Dnmt1 WB)

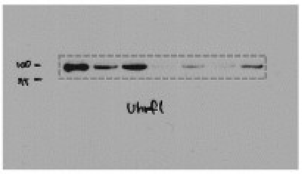

Supplementary Fig. 1d
(Uhrf1 WB)

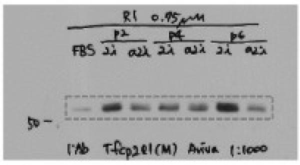

Supplementary Fig. 1d
(Tfcp2l1 WB)

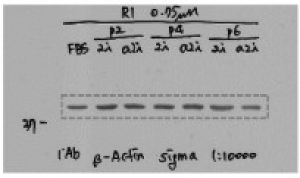

Supplementary Fig. 1d
(β-Actin WB)

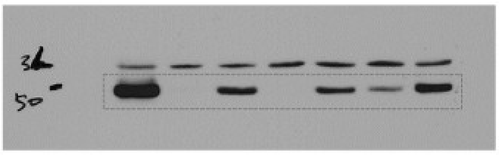

Supplementary Fig. 1e
(Dnmt3L WB)

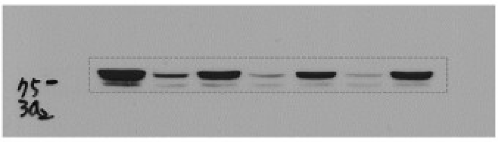

Supplementary Fig. 1e
(Dnmt3A2 WB)

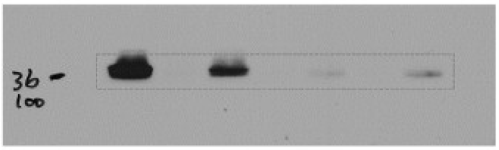

Supplementary Fig. 1e
(Dnmt3B WB)

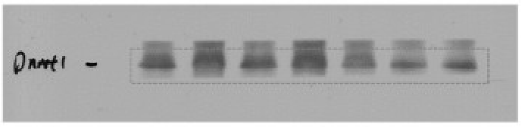


Supplementary Fig. 1e
(Dnmt1 WB)

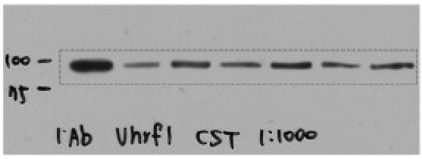


Supplementary Fig. 1e
(Uhrf1 WB)

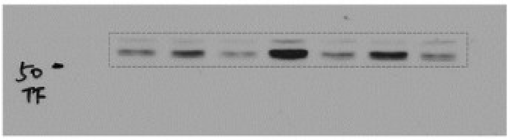

Supplementary Fig. 1e
(Tfcp2l1 WB)

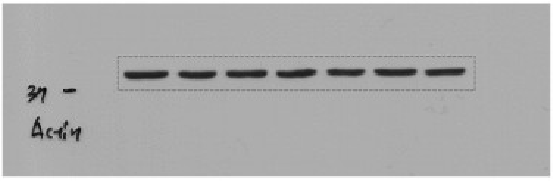


Supplementary Fig. 1e
(β-Actin WB)

**Supplementary Fig. 1**

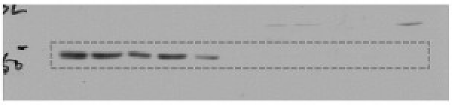

**Supplementary Fig. 1g**
**(Dnmt3L (s.e) WB)**

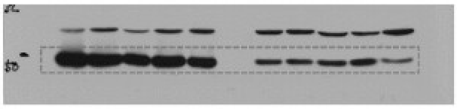

**Supplementary Fig. 1g**
**(Dnmt3L (l.e) WB)**

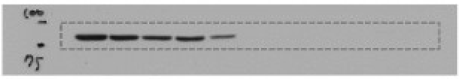

**Supplementary Fig. 1g**
**(Dnmt3A2 (s.e) WB)**

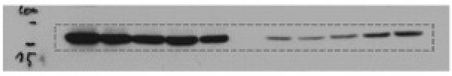

**Supplementary Fig. 1g**
**(Dnmt3A2 (l.e) WB)**

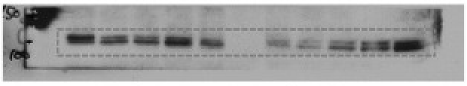

**Supplementary Fig. 1g**
**(Dnmt3B WB)**

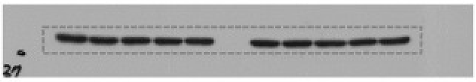

**Supplementary Fig. 1g**
**(β-Actin WB)**

**Supplementary Fig. 2**

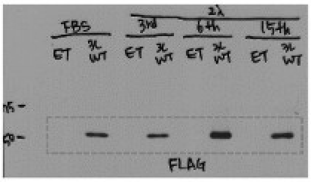

**Supplementary Fig. 2d**
**(FLAG WB)**

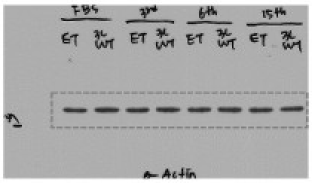

**Supplementary Fig. 2d**
**(β-Actin WB)**

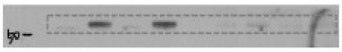

**Supplementary Fig. 2f**
**(FLAG-IP, p.3 Ac-K WB)**

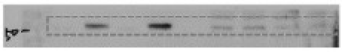

**Supplementary Fig. 2f**
**(FLAG-IP, p.6 Ac-K WB)**

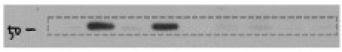

**Supplementary Fig. 2f**
**(FLAG-IP, p.15 Ac-K WB)**

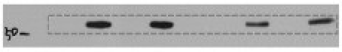

**Supplementary Fig. 2f**
**(FLAG-IP, p.3 FLAG WB)**

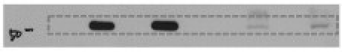

**Supplementary Fig. 2f**
**(FLAG-IP, p.6 FLAG WB)**

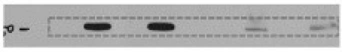

**Supplementary Fig. 2f**
**(FLAG-IP, p.15 FLAG WB)**

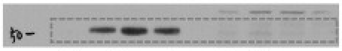

**Supplementary Fig. 2h**
**(FLAG-IP, a2i p.4 Ac-K WB)**

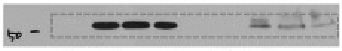

**Supplementary Fig. 2h**
**(FLAG-IP, a2i p.4 FLAG WB)**

**Supplementary Fig. 3**

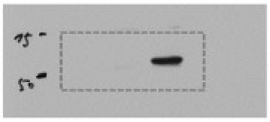

**Supplementary Fig. 3a**
**(FLAG WB)**

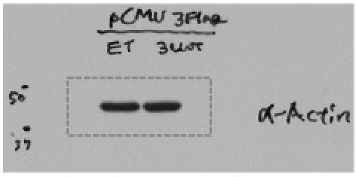

**Supplementary Fig. 3a**
**(β-Actin WB)**

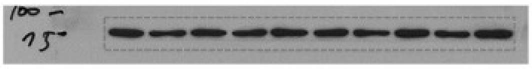

**Supplementary Fig. 3d**
**(Dnmt3A2 WB)**

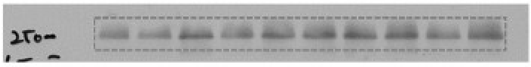

**Supplementary Fig. 3d**
**(Dnmt1 WB)**

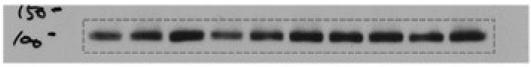

**Supplementary Fig. 3d**
**(Dnmt3B WB)**

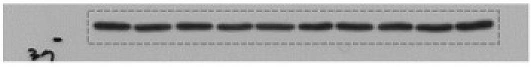

**Supplementary Fig. 3d**
**(β-Actin WB)**

**Supplementary Fig. 3**

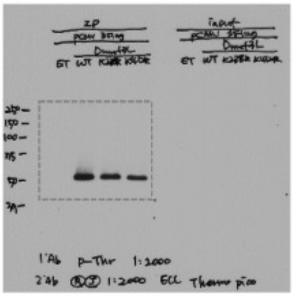

**Supplementary Fig. 3f**
**(FLAG-IP, p-Thr/Tyr WB)**

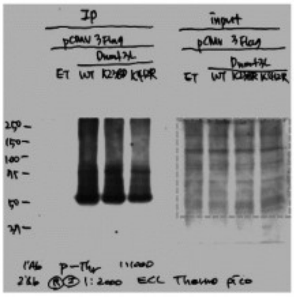

**Supplementary Fig. 3f**
**(Input, p-Thr/Tyr WB)**

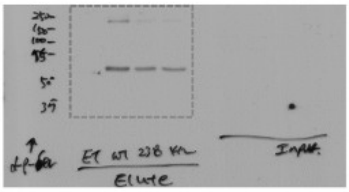

**Supplementary Fig. 3f**
**(FLAG-IP, p-Ser WB)**

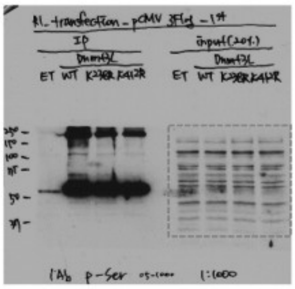

**Supplementary Fig. 3f**
**(Input, p-Ser WB)**

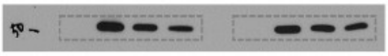

**Supplementary Fig. 3f**
**(FLAG-IP, FLAG WB)**

**Supplementary Fig. 5**

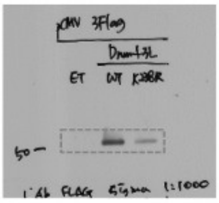

**Supplementary Fig. 5d**
**(p.1, K238R FLAG WB)**

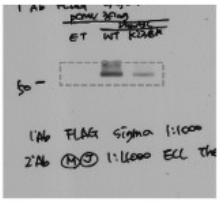

**Supplementary Fig. 5d**
**(p.4, K238R FLAG WB)**

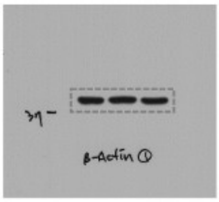

**Supplementary Fig. 5d**
**(p.1, K238R β-Actin WB)**

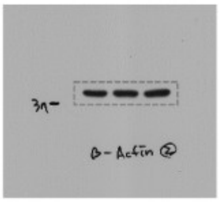

**Supplementary Fig. 5d**
**(p.4, K238R β-Actin WB)**

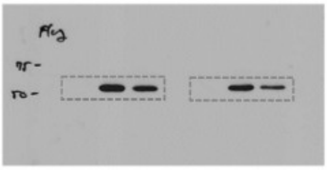

**Supplementary Fig. 5e**
**(p.1_p.4, K412R FLAG WB)**

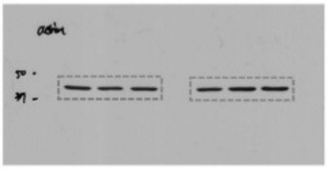

**Supplementary Fig. 5e**
**(p.1_p.4, K412R β-Actin WB)**

**Supplementary Fig. 6**

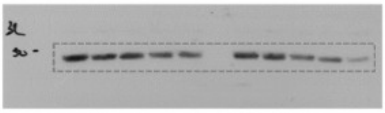

**Supplementary Fig. 6f**
**(K238R, Dnmt3L WB)**

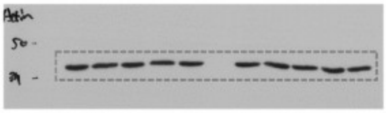

**Supplementary Fig. 6f**
**(K238R, β-Actin WB)**

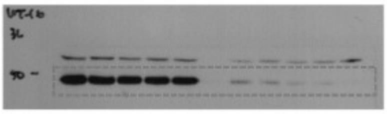

**Supplementary Fig. 6g**
**(K412R, Dnmt3L WB)**

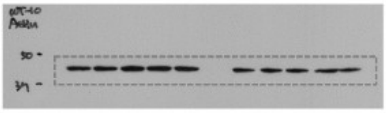

**Supplementary Fig. 6g**
**(K412R, β-Actin WB)**

**Supplementary Fig. 7**

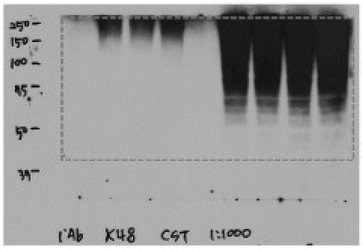


Supplementary Fig. 7a
(FLAG-IP, K48 (s.e) WB)

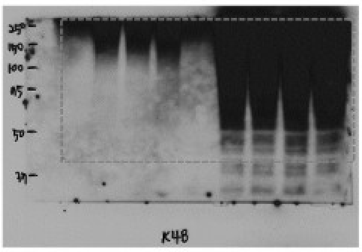


Supplementary Fig. 7a
(FLAG-IP, K48 (l.e) WB)

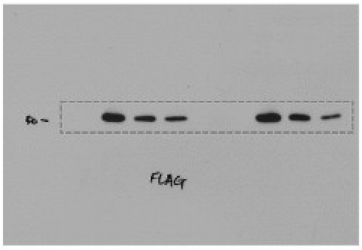


Supplementary Fig. 7a
(FLAG-IP, FLAG (K48) WB)

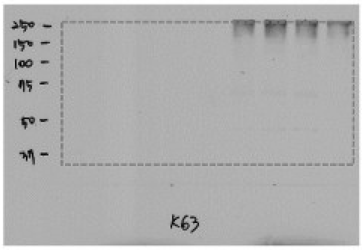


Supplementary Fig. 7b
(FLAG-IP, K63 (s.e) WB)

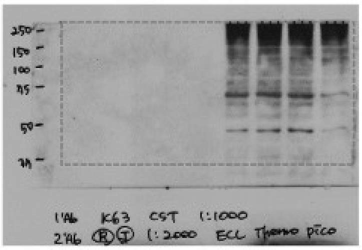

Supplementary Fig. 7b
(FLAG-IP, K63 (l.e) WB)

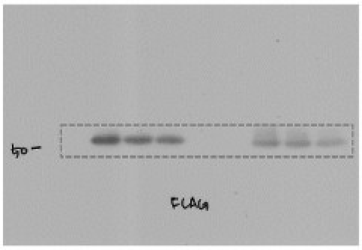


Supplementary Fig. 7b
(FLAG-IP, FLAG (K63) WB)

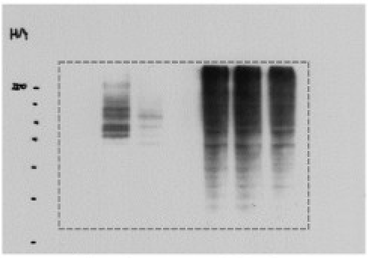

Supplementary Fig. 7c
(FLAG-IP, HA WB)

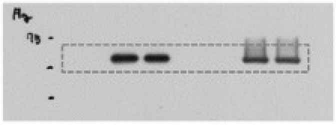

Supplementary Fig. 7c
(FLAG-IP, FLAG WB)

**Supplementary Fig. 8**

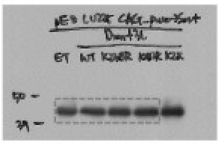

Supplementary Fig. 8c
(p.1, Oct4 WB)

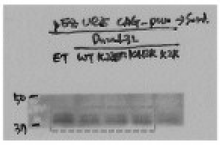

Supplementary Fig. 8c
(p.1, Klf2 WB)

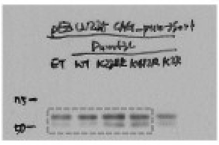

Supplementary Fig. 8c
(p.1, Klf4 WB)

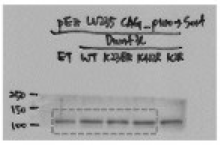

Supplementary Fig. 8c
(p.1, Sirt1 WB)

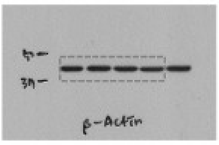


Supplementary Fig. 8c
(p.1, β-Actin WB)

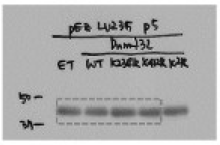

Supplementary Fig. 8c
(p.5, Oct4 WB)

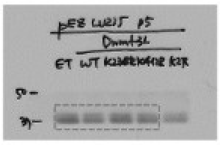

Supplementary Fig. 8c
(p.5, Klf2 WB)

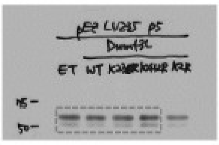

Supplementary Fig. 8c
(p.5, Klf4 WB)

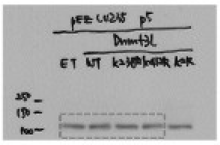

Supplementary Fig. 8c
(p.5, Sirt1 WB)

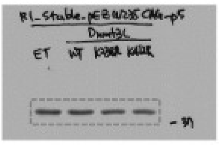

Supplementary Fig. 8c
(p.5, β-Actin WB)

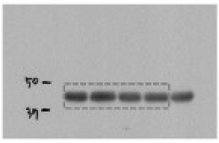

Supplementary Fig. 8c
(p.10 Oct4 WB)

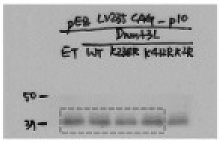

Supplementary Fig. 8c
(p.10, Klf2 WB)

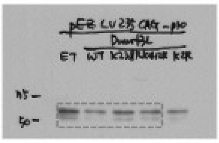

Supplementary Fig. 8c
(p.10, Klf4 WB)

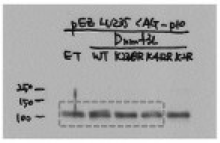

Supplementary Fig. 8c
(p.10, Sirt1 WB)

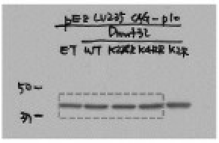

Supplementary Fig. 8c
(p.10, β-Actin WB)

**Supplementary Fig. 8**

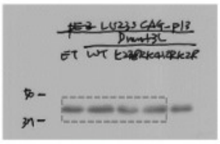

**Supplementary Fig. 8c (p.13, Oct4 WB)**

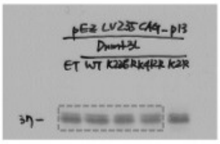

**Supplementary Fig. 8c (p.13, Klf2 WB)**

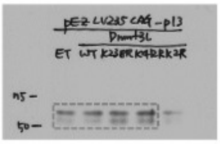

**Supplementary Fig. 8c (p.13, Klf4 WB)**

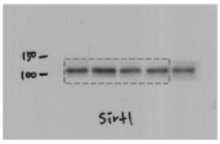

**Supplementary Fig. 8c (p.13, Sirt1 WB)**

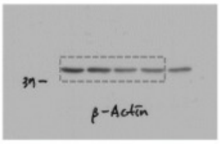

**Supplementary Fig. 8c (p.13, β-Actin WB)**

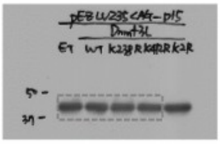

**Supplementary Fig. 8c (p.15, Oct4 WB)**

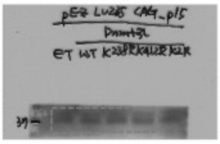

**Supplementary Fig. 8c (p.15, Klf2 WB)**

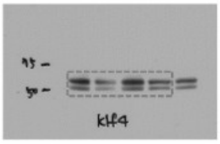

**Supplementary Fig. 8c (p.15, Klf4 WB)**

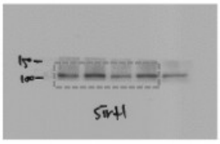

**Supplementary Fig. 8c (p.15, Sirt1 WB)**

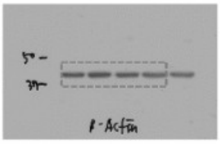

**Supplementary Fig. 8c (p.15, β-Actin WB)**

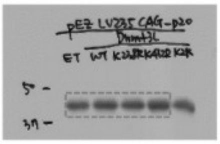

**Supplementary Fig. 8c (p.20 Oct4 WB)**

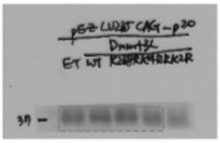

**Supplementary Fig. 8c (p.20, Klf2 WB)**

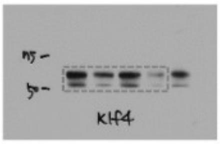

**Supplementary Fig. 8c (p.20, Klf4 WB)**

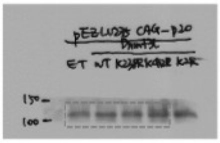

**Supplementary Fig. 8c (p.20, Sirt1 WB)**

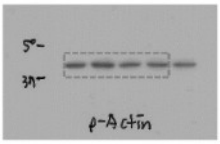

**Supplementary Fig. 8c (p.20, β-Actin WB)**

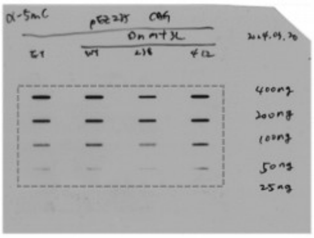

**Supplementary Fig. 8e (5mC Dotblot)**

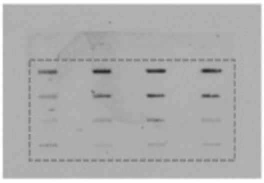

**Supplementary Fig. 8e (5hmC Dotblot)**

## SUPPLEMENTARY REFERENCES


1. Baubec, T. *et al.* Genomic profiling of DNA methyltransferases reveals a role for DNMT3B in genic methylation. *Nature* **520**, 243-247 (2015).
2. Subramanian, V. *et al.* H2A.Z acidic patch couples chromatin dynamics to regulation of gene expression programs during ESC differentiation. *PLoS Genet* **9**, e1003725 (2013).
3. Jumper, J. *et al.* Highly accurate protein structure prediction with AlphaFold. *Nature* **596**, 583-589 (2021).
4. Jo, S., Kim, T., Iyer, V. G. & Im, W. CHARMM-GUI: a web-based graphical user interface for CHARMM. *J Comput Chem* **29**, 1859-1865 (2008).
5. Lee, J. *et al.* CHARMM-GUI supports the Amber force fields. *J Chem Phys* **153**, 035103 (2020).
6. Lee, J. *et al.* CHARMM-GUI Input Generator for NAMD, GROMACS, AMBER, OpenMM, and CHARMM/OpenMM Simulations Using the CHARMM36 Additive Force Field. *J Chem Theory Comput* **12**, 405-413 (2016).
7. Lindorff-Larsen, K. *et al.* Improved side-chain torsion potentials for the Amber ff99SB protein force field. *Proteins* **78**, 1950-1958 (2010).
8. Abraham, M. J. *et al.* GROMACS: High performance molecular simulations through multi-level parallelism from laptops to supercomputers. *SoftwareX* **1-2**, 19-25 (2015).
9. Essmann, U. *et al.* A smooth particle mesh Ewald method. *The Journal of Chemical Physics* **103**, 8577-8593 (1995).
10. Hess, B., Bekker, H., Berendsen, H. J. C. & Fraaije, J. G. E. M. LINCS: A linear constraint solver for molecular simulations. *Journal of Computational Chemistry* **18**, 1463-1472 (1997).

11. Chen, P. J. *et al.* Enhanced prime editing systems by manipulating cellular determinants of editing outcomes. *Cell* **184**, 5635-5652.e5629 (2021).

12. Anzalone, A. V. *et al.* Search-and-replace genome editing without double-strand breaks or donor DNA. *Nature* **576**, 149-157 (2019).

13. Kim, S., Kim, D., Cho, S. W., Kim, J. & Kim, J. S. Highly efficient RNA-guided genome editing in human cells via delivery of purified Cas9 ribonucleoproteins. *Genome Res* **24**, 1012-1019 (2014).

14. Richardson, C. D., Ray, G. J., DeWitt, M. A., Curie, G. L. & Corn, J. E. Enhancing homology-directed genome editing by catalytically active and inactive CRISPR-Cas9 using asymmetric donor DNA. *Nature Biotechnology* **34**, 339-344 (2016).

15. Cullot, G. *et al.* Genome editing with the HDR-enhancing DNA-PKcs inhibitor AZD7648 causes large-scale genomic alterations. *Nature Biotechnology* https://doi.org/10.1038/s41587-024-02488-6 (2024).

16. Hwang, G.-H. *et al.* Large DNA deletions occur during DNA repair at 20-fold lower frequency for base editors and prime editors than for Cas9 nucleases. *Nature Biomedical Engineering* **9**, 79-92 (2025).

17. Park, J., Bae, S. & Kim, J.-S. Cas-Designer: a web-based tool for choice of CRISPR-Cas9 target sites. *Bioinformatics* **31**, 4014-4016 (2015).

18. Bae, S., Park, J. & Kim, J.-S. Cas-OFFinder: a fast and versatile algorithm that searches for potential off-target sites of Cas9 RNA-guided endonucleases. *Bioinformatics* **30**, 1473-1475 (2014).

19. Lim, J. *et al.* Small-sized mesenchymal stem cells with high glutathione dynamics show improved therapeutic potency in graft-versus-host disease. *Clin Transl Med* **11**, e476 (2021).

20. Yu, H. Y. *et al.* Intravital imaging and single cell transcriptomic analysis for

engraftment of mesenchymal stem cells in an animal model of interstitial cystitis/bladder pain syndrome. *Biomaterials* **280**, 121277 (2022).

21. Kim, Y. *et al.* Small hypoxia-primed mesenchymal stem cells attenuate graft-versus-host disease. *Leukemia* **32**, 2672-2684 (2018).

22. Lee, S. *et al.* Ascorbic Acid 2-Glucoside Stably Promotes the Primitiveness of Embryonic and Mesenchymal Stem Cells Through Ten-Eleven Translocation- and cAMP-Responsive Element-Binding Protein-1-Dependent Mechanisms. *Antioxid Redox Signal* **32**, 35-59 (2020).

23. Koo, K. M. *et al.* Label-free and non-destructive identification of naive and primed embryonic stem cells based on differences in cellular metabolism. *Biomaterials* **293**, 121939 (2023).

24. Keller, G. M. In vitro differentiation of embryonic stem cells. *Curr. Opin. Cell Biol.* **7**, 862-869 (1995).

25. Mao, X. & Zhao, S. Neuronal Differentiation from Mouse Embryonic Stem Cells In vitro. *J Vis Exp* https://doi.org/10.3791/61190, e61190 (2020).

26. Heo, J. *et al.* Sirt1 Regulates DNA Methylation and Differentiation Potential of Embryonic Stem Cells by Antagonizing Dnmt3l. *Cell Rep* **18**, 1930-1945 (2017).

27. Heo, J. *et al.* Phosphorylation of TFCP2L1 by CDK1 is required for stem cell pluripotency and bladder carcinogenesis. *EMBO Mol Med* **12**, e10880 (2020).

28. Lim, J. *et al.* Glutathione dynamics determine the therapeutic efficacy of mesenchymal stem cells for graft-versus-host disease via CREB1-NRF2 pathway. *Science Advances* **6**, eaba1334 (2020).

29. FastQCed^eds), 2015

30. Chen, S., Zhou, Y., Chen, Y. & Gu, J. fastp: an ultra-fast all-in-one FASTQ preprocessor. *Bioinformatics (Oxford, England)* **34**, i884-i890 (2018).

31. Dobin, A. *et al.* STAR: ultrafast universal RNA-seq aligner. *Bioinformatics (Oxford, England)* **29**, 15-21 (2013).

32. Patro, R., Duggal, G., Love, M. I., Irizarry, R. A. & Kingsford, C. Salmon provides fast and bias-aware quantification of transcript expression. *Nat Methods* **14**, 417-419 (2017).

33. Liberzon, A. *et al.* Molecular signatures database (MSigDB) 3.0. *Bioinformatics (Oxford, England)* **27**, 1739-1740 (2011).

34. Xu, S. *et al.* Using clusterProfiler to characterize multiomics data. *Nature protocols* **19**, 3292-3320 (2024).
